# Wide-scope targeted analysis of bioactive lipids in human plasma by LC/MS/MS

**DOI:** 10.1016/j.jlr.2023.100492

**Published:** 2023-12-20

**Authors:** Kohta Nakatani, Yoshihiro Izumi, Hironobu Umakoshi, Maki Yokomoto-Umakoshi, Tomoko Nakaji, Hiroki Kaneko, Hiroshi Nakao, Yoshihiro Ogawa, Kazutaka Ikeda, Takeshi Bamba

**Affiliations:** 1 Division of Metabolomics/Mass Spectrometry Center, Medical Research Center for High Depth Omics, Medical Institute of Bioregulation, Kyushu University, Fukuoka, Japan; 2 Department of Medicine and Bioregulatory Science, Graduate School of Medical Sciences, Kyushu University, Fukuoka, Japan; 3 Laboratory of Biomolecule Analysis, Department of Applied Genomics, Kazusa DNA Research Institute, Kisarazu, Chiba, Japan

**Keywords:** steroid, bile acid, polyunsaturated fatty acid, liquid chromatography, tandem mass spectrometry, solid-phase extraction, comprehensive analysis

## Abstract

Quantitative information on blood metabolites can be used in developing advanced medical strategies such as early detection and prevention of disease. Monitoring bioactive lipids such as steroids, bile acids, and PUFA metabolites could be a valuable indicator of health status. However, a method for simultaneously measuring these bioactive lipids has not yet been developed. Here, we report a LC/MS/MS method that can simultaneously measure 144 bioactive lipids, including steroids, bile acids, and PUFA metabolites, from human plasma, and a sample preparation method for these targets. Protein removal by methanol precipitation and purification of bioactive lipids by solid-phase extraction improved the recovery of the targeted compounds in human plasma samples, demonstrating the importance of sample preparation methods for a wide range of bioactive lipid analyses. Using the developed method, we studied the plasma from healthy human volunteers and confirmed the presence of bioactive lipid molecules associated with sex differences and circadian rhythms. The developed method of bioactive lipid analysis can be applied to health monitoring and disease biomarker discovery in precision medicine.

## Graphical abstract

**Figure:**
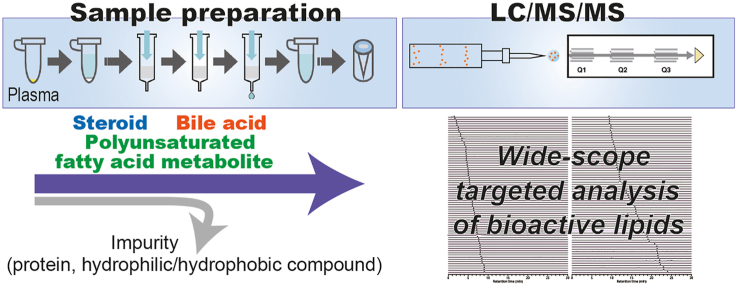

The objective assessment of health status using information from blood metabolites can be applied to medical strategies such as early detection and prevention of disease (1). Metabolomics, which mainly focuses on the comprehensive monitoring of hydrophilic metabolites, has been widely applied in biomarker discovery (2), and the number of reports is increasing (3). However, the number of clinical applications is stagnating (3) despite the increase in biomarker research. Ongoing cohort studies have reported that the concentrations of some amino acids increase with age and BMI (4), while other studies have reported no significant differences in amino acid levels with age or BMI and a decrease in amino acid levels due to smoking (5). In these studies, despite the differences in sample size and race, several cross-reactions were present in the hydrophilic primary metabolites owing to various factors such as age, BMI, and smoking status. Consequently, their success in clinical practice may be limited (6).

Steroids are a group of molecules that are biosynthesized from cholesterol, mainly in the adrenal glands, transported through the blood to various organs, and exert their physiological activity by binding to membrane and nuclear receptors (7). Measurements of aldosterone [primary aldosteronism (8, 9)], cortisol [Cushing's syndrome (10, 11), adrenal cortical carcinoma (12)], and progesterone [ovulation status (13)] are widely used in clinical practice. Bile acids are biosynthesized from cholesterol primarily in the liver, concentrated in the gallbladder, and excreted in the intestine. Excreted primary bile acids are either absorbed or circulated in the enterohepatic circulation after being converted into secondary bile acids by intestinal bacteria (14). Recently, bile acids have received considerable attention because they stimulate various immune systems (15). PUFA metabolites, such as eicosanoids and docosanoids, are biosynthesized from fatty acids cleaved by phospholipase A2 from phospholipids that constitute cell membranes (16, 17). The recent characterization of individual eicosanoid receptors has enabled a better understanding of their regulation of inflammation and anti-inflammatory effects on key players in the immune system (16). Therefore, information on the in vivo profile of these bioactive lipids (such as steroids, bile acids, and PUFA metabolites) may be a promising indicator of health status.

When analyzing targeted endogenous metabolites in biological samples, appropriate sample preparation and analytical methods are crucial (18). LC/MS is often used in metabolomics to simultaneously analyze multiple compounds (19, 20). However, analyzing a wide range of biological compounds, from hydrophilic [such as ATP, the *n*-octanol/water partition coefficient, log *P*_ow_ = −5.5] to highly hydrophobic [such as triacylglycerol (TG) 18:0_18:0_18:0, log *P*_ow_ = 17.41] compounds, is difficult using a single LC/MS technique. Therefore, a comprehensive analysis of a wide range of metabolome is underway by combining multiple measurement techniques and sample preparation methods (21). To date, LC/MS analytical methods have been developed and individually monitored to measure metabolites in steroids (22), bile acids (23), and PUFA metabolites (24) for research purposes. However, no method has been reported to measure these groups of compounds across the board. The log *P*_ow_ values for cortisol, cholic acid, and arachidonic acid, which are representative examples of steroids, bile acids, and PUFA metabolites, were 1.61, 2.02, and 6.98, respectively. Thus, these three groups exhibited similar physicochemical properties from the perspective of the entire metabolome (25). Therefore, a detailed evaluation of the analytical system, using a set of authentic standards, may allow the development of a simultaneous assay for these bioactive lipids.

Furthermore, the importance of sample preparation methods has recently been reevaluated, in particular by the lipidomics standard initiative and the international lipidomics society (26). The role of sample preparation in the analysis of trace metabolites such as bioactive lipids is to purify the component of interest and remove impurities. Failure to understand the role of sample preparation can lead to unexpected problems. For example, without proper pretreatment, contaminants, such as denatured proteins or precipitated highly hydrophobic lipids, can accumulate in LC columns. Consequently, pump pressure may increase as the number of analyses increases, which may reduce the reproducibility and accuracy of the analysis (27). In addition, unwanted hydrophilic compounds can cause ion suppression in the targeted compounds and reduce the sensitivity of LC/MS analysis, thus rendering proper sample preparation an essential step in obtaining high-quality data (28).

The purpose of this study is to propose a LC/MS/MS in multiple reaction monitoring (MRM) or selected ion monitoring modes and sample preparation method for comprehensive, simultaneous, and quantitative analysis of bioactive lipids, such as steroids, bile acids, and PUFA metabolites, for monitoring human health status using blood samples. First, we optimized MRM/selected ion monitoring conditions for 144 bioactive lipids and stable isotope-labeled internal standards (ISs) using triple-quadrupole mass spectrometry (QqQMS). The LC column and mobile phase conditions were investigated to determine the optimal LC separation conditions for bioactive lipids. An optimized LC/MS/MS method was established for the simultaneous analysis of 144 bioactive lipids. The sample preparation method was evaluated based on the removal of contaminants from human plasma and the recovery rate of the target compound. The optimized sample preparation and LC/MS/MS methods were used to measure bioactive lipids in human plasma from healthy volunteers and analyze sex and intraday variations. To the best of our knowledge, this is the first report of the cross-sectional quantitative measurement of a group of compounds including steroids, bile acids, and PUFA metabolites.

## Materials and methods

### Chemicals and reagents

LC/MS-grade water, acetonitrile, methanol, and 2-propanol were purchased from Kanto Chemical Co, Inc (Tokyo, Japan). LC/MS-grade ammonium acetate was purchased from Merck (Darmstadt, Germany). LC/MS-grade acetic acid was purchased from Fujifilm Wako Pure Chemical Co (Osaka, Japan). Standards were obtained from Nacalai Tesque, Inc (Kyoto, Japan), Fujifilm Wako Pure Chemical Co, and Merck. Stable isotope-labeled standards were obtained from Cayman Chemical Co (Ann Arbor, MI), Cambridge Isotope Laboratories, Inc (Tewksbury, MA), Steraloids Inc (Newport, RI), Avanti Polar Lipids Inc (Alabaster, AL), and Merck. Compound names and abbreviations of the 144 bioactive lipid standards used in this study are listed in supplemental Table S1.

### Calculation of the structural properties

Calculations of log *P*_ow_, strongest acidic p*K*_a_, and strongest basic p*K*_b_ for the compounds evaluate in this study were performed by uploading simplified molecular input line entry system notations extracted from the PubChem database (25) to the Online Chemistry Database web platform (29). All log *P*_ow_ values were calculated using the AlvaDesc model and p*K*_a_ and p*K*_b_ were calculated using the ChemaxonDescriptors model (supplemental Table S1).

### Collection of human plasma

This study was approved by the Ethics Committee of the Kyushu University (No. 21025-00) and was performed in accordance with the Declaration of Helsinki between April 2021 and March 2022. Plasma samples were collected from eight healthy adult volunteers; five males, and three premenopausal females, all of whom were below the age of 40 (supplemental Table S2). Blood samples were collected from males in the morning (8:00–9:00), noon (12:00–13:00), and evening (17:00–18:00). Blood was collected from the females in the morning (8:00–9:00). All the plasma samples were collected before meals and centrifuged. The resulting supernatants were stored in a −80°C freezer until analysis. To evaluate the pretreatment method, equal amounts of mixed plasma from healthy volunteers were used as reference samples.

### Human plasma sample preparation

The plasma was thawed at 4°C for approximately (approx.) 12 h. A mixture of 50 μl of plasma, 500 μl of methanol, and 10 μl of IS containing 28 stable isotope-labeled compounds in methanol (supplemental Table S3) was vortexed for 1 min, followed by sonication for 5 min. The samples were incubated on ice for 5 min, followed by centrifugation at 4°C, 16,000 *g* for 5 min to precipitate proteins. The protein concentrations in the pellets were determined using a Pierce™ BCA Protein Assay Kit (Thermo Fisher Scientific, MA). The collected supernatant (500 μl) was transferred to a 2 ml Eppendorf tube and mixed with 1,500 μl of water/formic acid (FA) (100/0.1, vol/vol) to obtain a sample for solid-phase extraction (SPE) after protein removal by methanol precipitation. Another sample for SPE without protein removal by methanol precipitation was prepared by mixing 50 μl of plasma with 950 μl of water/methanol (75/25, vol/vol) and 10 μl of ISs in methanol, followed by vortexing for 1 min and then subjected to sonication for 5 min.

For SPE, an OASIS HLB 1 cc (30 mg) cartridge (Waters, Milford, MA) was placed in a vacuum manifold and equilibrated by serially passing 1 ml of methanol/FA (100/0.1, vol/vol) and 1 ml of water/FA (100/0.1, vol/vol). The preprepared SPE samples were loaded onto a cartridge. Different solvent combinations were used, including water/FA (100/0.1, vol/vol), ethanol/water/FA (15/85/0.1, vol/vol/vol), methanol/water/FA (10/90/0.1, vol/vol/vol), methanol/water/FA (20/80/0.1, vol/vol/vol), methanol/water/FA (40/60/0.1, vol/vol/vol), and hexane. The abbreviations for each SPE method [methanol precipitation (MeP)-SPE1 to MeP-SPE6] and the detailed procedure are described in supplemental Table S4. To investigate protein removal, the SPE cartridge was washed with water/FA (100/0.1, vol/vol), ethanol/water/FA (15/85/0.1, vol/vol/vol), and hexane [described as methanol precipitation, followed by the SPE method (MeP-SPE1) in supplemental Table S4]. The optimized sample preparation procedure was MeP-SPE1, followed by the recovery of the targeted bioactive lipids with 1 ml of methanol. The 1 ml of methanol in a 1.5-ml Eppendorf tube was dried using a centrifugal evaporator and then reconstituted by vortexing with 60 μl of water/methanol (1/1, vol/vol). After incubation on ice for 5 min, the samples were centrifuged at 4°C, 16,000 *g* for 5 min, and the resulting 50 μl supernatant was transferred to a 0.3 ml Low Adsorption Vial QsertVial (Merck) for LC/MS/MS analysis.

### Analytical conditions for LC/MS/MS

The LC/MS/MS analyses were performed using a Nexera X2 UHPLC system (Shimadzu Co, Kyoto, Japan) coupled to an LCMS-8060 QqQMS (Shimadzu Co) equipped with a heated electrospray ionization source. The LC mobile phase conditions used to evaluate the separation behavior were as follows: mobile phase (A), 5 mM ammonium acetate or 0.1% (vol/vol) acetic acid in water/acetonitrile (75/25, vol/vol); and mobile phase (B), 5 mM ammonium acetate or 0.1% (vol/vol) acetic acid in 2-propanol. The four LC columns used to compare the separation of bioactive lipids were Inertsil ODS-4, InertSustain C18, Inertsil ODS-HL, and Inertsil ODS-P [each, 2.1 mm inner diameter × 150 mm, 3 μm particle size, GL Sciences Inc, Tokyo, Japan]. The simplified gradient conditions used to compare the separation behaviors were as follows: 1% B, 0 min; 1–99% B, 0–20 min; 99% B, 20–35 min; 99–1% B, 35–35.1 min; and 1% B, 35.1–45 min. Other LC conditions used for condition screening were as follows: injection volume, 10 μl; flow rate, 0.3 ml min^−1^; and column temperature, 40°C.

The optimized final LC analysis conditions used for the plasma analyses were as follows: column, InertSustain C18; injection volume, 10 μl; flow rate, 0.3 ml min^−1^; column temperature, 50°C; mobile phase (A), 5 mM ammonium acetate in water/acetonitrile 75/25 (vol/vol); and mobile phase (B), 5 mM ammonium acetate in 2-propanol. The optimized gradient conditions were as follows: 1% B, 0 min; 1–38% B, 0–17 min; 38–99% B, 17–25 min; 99% B, 25–35 min; 99–1% B, 35–36 min; and 1% B, 36–46 min.

The MS conditions used for all analyses were as follows: nebulizer gas flow, 2 l min^−1^; heating gas flow, 10 l min^−1^; drying gas flow, 10 l min^−1^; heat block temperature, 400°C; desolvation line temperature, 250°C; and spray voltage, 4 kV for positive-ion mode and −3 kV for negative-ion mode. The MRM/SIM mode was applied to all targeted LC/MS/MS analyses. The MRM/SIM parameters were as follows: dwell time, 2 ms; pause time, 2 ms; and polarity switching time, 5 ms. Other MRM conditions, including Q1 prebias, collision energy, and Q3 prebias of each metabolite, were optimized by flow injection analysis with standard solutions (1–100 μM) using LabSolution ver. 5.91 (Shimadzu Co). The details of the optimized MRM/SIM parameters for the 144 targeted metabolites and 28 IS compounds are presented in supplemental Table S5. To construct calibration curves for each metabolite, standard solutions were prepared using water/methanol (50/50, vol/vol) at concentrations of 0, 0.1, 1, 4, 10, 40, 100, 400, 1,000, 4,000, and 10,000 nM. In the LC/MS full-scan mode, a scan range of *m/z* 100–1,000 was used to estimate the off-target matrix components for each positive and negative ionizations. The analytical platform for bioactive lipid analysis was controlled using LabSolutions (version 5.99 SP2, Shimadzu Co).

### Data analysis

LC/MRM and LC/SIM data analysis was performed using Multi-ChromatoAnalysT (BeForce Co, Fukuoka, Japan). Principal component analysis was performed using MetaboAnalyst 5.0 (30). Statistical significance was determined using paired *t*-test.

## Results

### Calculation of physicochemical properties of targeted compounds and optimization of MRM/SIM conditions

In this study, we developed a simultaneous and quantitative analytical method for 144 unconjugated and conjugated forms of bioactive lipids (steroids, bile acids, and PUFA metabolites), including glucuronide, sulfate, glycine, and taurine (supplemental Table S1). Physicochemical properties such as hydrophobicity and charge are the major factors that affect the separation behavior of LC or the extraction and recovery efficiency of biological samples. Considering the physicochemical properties of the targeted metabolites, reasonable sample preparation and analytical methods can be proposed. Therefore, we obtained the log *P*_ow_, p*K*_a_, and p*K*_b_ of the targeted metabolites using Online Chemistry Database, a web platform for calculating various chemical parameters (29) (supplemental Table S1). The ranges of log *P*_ow_, p*K*_a_, and p*K*_b_ values for the targeted steroids, bile acids, and PUFA metabolites were 0.58–6.70, −1.75 to 19.78, and −7.48 to 9.31, respectively. These values were used to review and optimize the analytical and sample preparation methods. LC/MS/MS in MRM mode has attracted attention for widely targeted metabolome analysis because of its selectivity, high sensitivity, and good quantitative performance (19, 20). The MRM or SIM transitions (precursor ion, collision energy, product ion, and prequadrupole focusing voltage) of the 144 metabolites and 28 stable isotope-labeled compounds were optimized by flow injection analysis of each authentic standard, with up to two MRM transitions for each metabolite (supplemental Table S5). MRM/SIM transition 1 was used for quantification.

### Optimization of LC conditions

Reversed-phase LC is suitable for the separation of hydrophobic compounds. In this study, we defined the characteristics of four ODS columns with different hydrophobicities and stereoselectivities using the Tanaka method of silica ODS column characterization (31) (supplemental Table S6). The gradient mode from a water–acetonitrile mixture to 2-propanol, which has excellent elution power in reversed-phase LC, was used. However, the high viscosity of 2-propanol resulted in an increase in column back pressure. Under screening LC conditions, Inertsil ODS-4, InertSustain C18, and Inertsil ODS-HL exhibited no pressure problems, with maximum pressures of 42, 37, and 45 MPa, respectively, whereas Inertsil ODS-P exceeded the pressure limit (>50 MPa). Therefore, Inertsil ODS-P was excluded from further analyses.

**Fig. 1:**
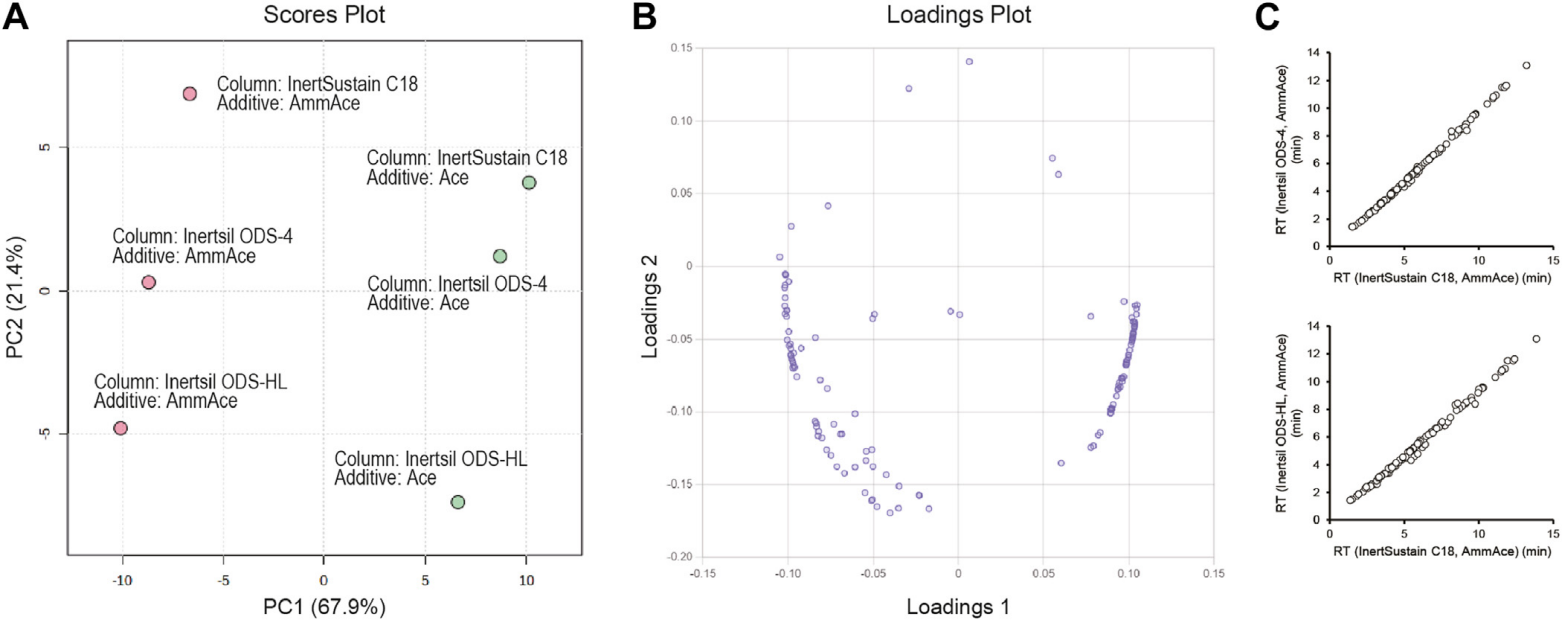
Characterization of the retention behavior of bioactive lipid standards on three different columns and two different LC conditions using PCA analysis. The RTs of bioactive lipid compounds that were consistently detected under all screening conditions were used. A: Score plots. Red and green dots indicate ammonium acetate and acetic acid addition conditions, respectively. B: Loading plots. C: Comparison of the RT between Inertsil ODS-4, InertSustain C18, and Inertsil ODS-HL columns under ammonium acetate addition conditions. PCA, principal component analysis; RT, retention time.

After analyzing the targeted bioactive lipid standards under six different LC conditions (using three columns and two sets of mobile phases, ammonium acetate, and acetic acid additives) with common simplified gradient conditions (see Materials and methods), 128 compounds were detected as single peaks. Peak information, such as retention time (RT) and peak width, for each detected compound, is summarized in supplemental Table S7. To evaluate the effect of six different LC analytical conditions with three different columns and two additives on the retention behavior of the bioactive lipids, principal component analysis was performed using the RTs for each compound. The score plots showed that each LC condition could be represented by PC1 (67.9%) and PC2 (21.4%), and the differences in RTs were clearly reflected in these conditions (Fig. 1A). Among the ODS columns studied, the type of additive had the most significant effect. Of the 66 compounds with a loading (1) value of <0, only 14 (21.2%) exhibited a p*K*_a_ <5, whereas of the 62 compounds with a loading (1) value of >0, 58 (93.5%) exhibited a p*K*_a_ <5 (Fig. 1B and supplemental Table S8). It was assumed that the difference in additives between ammonium acetate and acetic acid would significantly affect the retention of acidic compounds. As the acetic acid additive produces broad tailing peaks for several compounds, ammonium acetate was selected as the additive in this study. Additives are important for chromatographic performance, especially for compounds with a wide range of physicochemical properties such as steroids, bile acids, and PUFA metabolites. However, no differences in the elution pattern of bioactive lipid molecules or the separation performance of isomer pairs were observed between Inertsil ODS-4, InertSustain C18, and Inertsil ODS-HL (Fig. 1C and supplemental Fig. S1). Therefore, InertSustain C18 was selected for its ability to provide stable measurements at the lowest column back pressure.

**Fig. 2:**
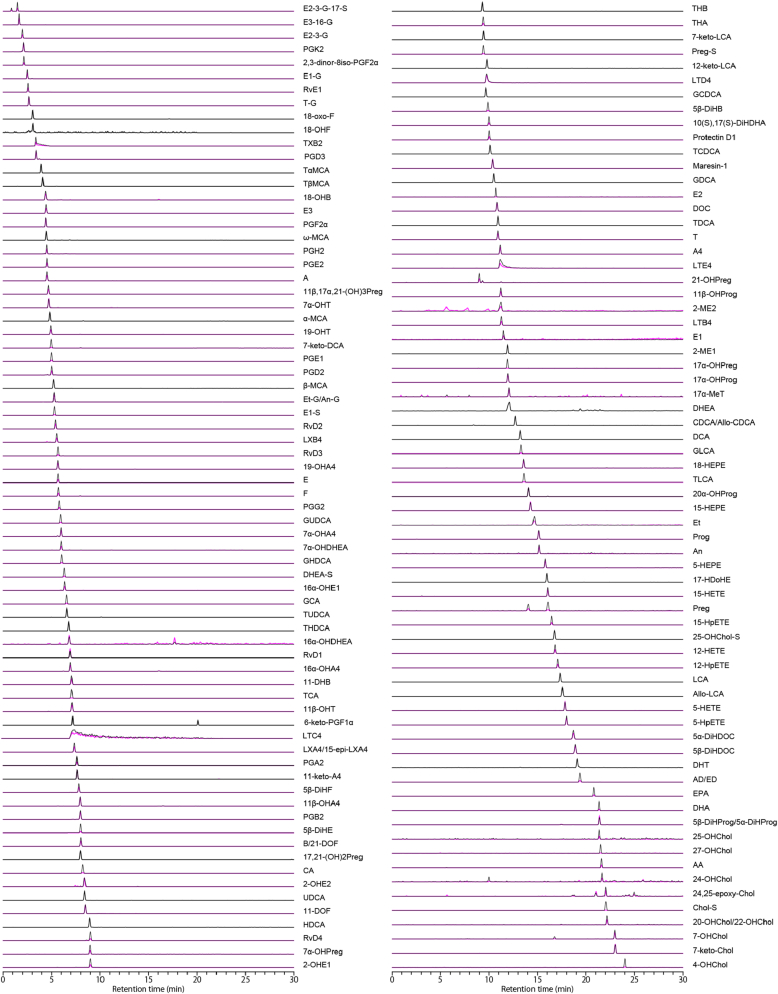
Separation overview of an optimized LC/MS/MS method. Black chromatograms represent MRM/SIM transition 1 for quantification, whereas the chromatograms in pink represent MRM transition 2 for qualification. All compound abbreviations and MRM/SIM conditions are listed in supplemental Table S5. MRM, multiple reaction monitoring; SIM, selected ion monitoring.

Finally, we optimized the LC conditions to improve chromatographic separation and increase the stability of the analysis. Specifically, to improve the separation of weakly retained compounds, the gradient slope in the first half of the analysis was adjusted to be more gradual, and the column temperature was increased from 40°C to 50°C to reduce column back pressure. The LC/MRM or LC/SIM chromatograms obtained using an InertSustain C18 column under optimized conditions are shown in Fig. 2. The analytical validation results are shown in supplemental Table S5. Dilution series of each standard were prepared by dissolution in water/methanol (50/50, vol/vol) without matrix, and the lower and upper limits of quantification of the LC/MS/MS analytical system were calculated. The developed analytical method enabled the measurement of a wide range of steroids, bile acids, and PUFA metabolites.

### Evaluation of a sample preparation method for wide-scope targeted bioactive lipids analysis

We investigated an optimal sample preparation method for human plasma, assuming that proteins, hydrophilic metabolites, and highly hydrophobic metabolites, such as phospholipids and neutral lipids, interfere with the comprehensive, sensitive, and quantitative LC/MS/MS measurements of bioactive lipids.

Proteins can be removed as precipitates by mixing them with organic solvents, trapping them in SPE cartridges, and washing them with solvents during the SPE process. Three methods were evaluated for protein removal using reference human plasma samplesMeP, SPE, and MeP followed by SPE (MeP-SPE1) (supplemental Table S4). The SPE method used in this study is based on a previously reported method for eicosanoids (32). All three methods (MeP, SPE, and MeP-SPE1) showed removal efficiencies >97% for proteins (97.7%, 98.5%, and 99.6%, respectively). These methods are effective in adequately removing proteins from human plasma.

**Fig. 3:**
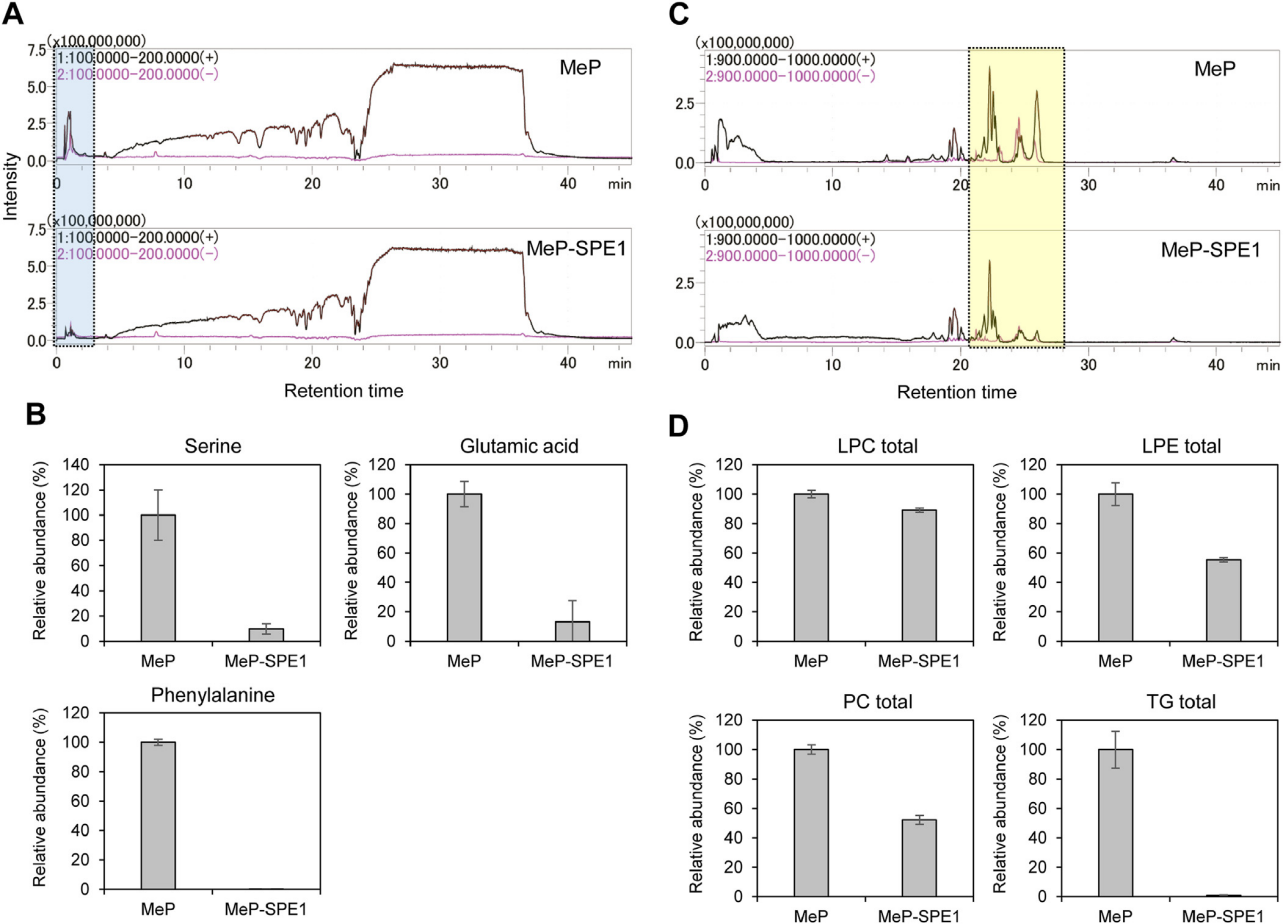
Effect of removal of hydrophilic compounds and highly hydrophobic compounds from plasma extracts on bioactive lipids analysis. A, B: Evaluation of the removal of low-molecular-weight hydrophilic compounds. C, D: Evaluation of highly hydrophobic compound removal. LC/MS TIC chromatograms of MeP-treated supernatants and MeP-SPE elution fractions of human plasma extracts measured at *m/z* 100–200 (A) or *m/z* 900‒1,000 (C) in positive-ion mode (black) and negative-ion mode (pink). Comparison of relative abundance of three amino acids (B) and four lipid classes (D) in human plasma extracts after treatment with MeP and MeP-SPE1 methods. Values are mean ± SD (n = 3). MeP, methanol precipitation; SPE, solid-phase extraction; TIC, total ion current.

Hydrophilic compounds with molecular weights <200, such as amino acids, should be removed by SPE in the washing step with aqueous solvents. In LC/MS using an ODS column, hydrophilic compounds are typically not retained and are eluted immediately after sample injection. Therefore, to confirm the removal of hydrophilic compounds from the plasma methanol extract, SPE was performed by changing the set of washing solvents (supplemental Table S4). The MeP-treated supernatant and eluted fraction after MeP-SPE were measured using optimized LC/MS in full-scan mode (supplemental Fig. S2). Comparison of the MeP-treated supernatant with the MeP-SPE elution fraction showed that the ionic intensity of the total ion current in the *m/z* 100–200 range was reduced in the MeP-SPE elution fraction with a RT of <2.5 min (Fig. 3A). In addition, the representative amino acids, including serine, glutamic acid, and phenylalanine, were measured by adding these MRM transitions to the LC/MS/MS method for bioactive lipid analysis (supplemental Table S9). The removal efficiencies by MeP-SPE1 for serine, glutamic acid, and phenylalanine detected and quantified by MeP method were 90.0, 86.8, and 99.8%, respectively (Fig. 3B). These results indicated that SPE effectively removed hydrophilic compounds from human plasma methanol extracts.

We then tested whether abundant highly hydrophobic compounds, including phospholipids [e.g., lysophosphatidylcholine (LPC); lysophosphatidylethanolamine (LPE); and phosphatidylcholine (PC)] and neutral lipids (e.g., TG), are removed from human plasma by the SPE washing process or by trapping in the SPE cartridge. Comparing the MeP-treated supernatant with the MeP-SPE elution fraction, the MeP-SPE elution fraction showed a decrease in the ionic intensity of total ion current in the *m/z* 900–1,000 range at RTs of 20–28 min (Fig. 3C), suggesting the removal of highly hydrophobic compounds from human plasma by SPE. Therefore, LC/MRM measurements of LPC, LPE, PC, and TG lipid classes were performed to evaluate in detail the effects of abundant phospholipid and neutral lipid residues in plasma samples on bioactive lipid analysis. Lipid molecules with six fatty acyl side chains, including FA16:0, FA18:0, FA18:1, FA18:2, FA20:4, and FA22:6, for four lipid classes were used to set up MRM transitions for LC/MS/MS measurements (supplemental Table S9). The RT ranges for LPCs, LPEs, PCs, and TGs were 19.3–21.7, 19.7–22.0, 23.6–25.0, and 26.7–27.3 min, respectively. The removal efficiencies by MeP-SPE1 for the quantified values of LPC, LPE, PC, and TG lipid classes detected by MeP method were 11, 44.5, 47.8, and 99.0%, respectively (Fig. 3D). The MeP-SPE procedure is capable of removing most or some of the proteins, hydrophilic compounds, and highly hydrophobic compounds that interfere with the analysis of trace amounts of bioactive lipids from human plasma.

Next, the recovery rates of the target bioactive lipids from human plasma samples were investigated. Twenty-eight stable isotope-labeled ISs of steroids, bile acids, and PUFA metabolites were prepared and the recovery rates were calculated when MeP-SPE was performed alone or with plasma (Table 1). When evaluating ISs without plasma, MeP-SPE1 yielded the highest number of 26 compounds with IS recoveries >70%. Therefore, we used MeP-SPE1 for plasma analysis in this study.

**Table 1:** Recovery rate of steroids, bile acids, and PUFA metabolites from human plasma using stable isotope-labeled ISs

Sample		IS							Human plasma with IS					
Method		MeP (control)	MeP-SPE1	MeP-SPE2	MeP-SPE3	MeP-SPE4	MeP-SPE5	MeP-SPE6	MeP-SPE1	MeP-SPE2	MeP-SPE3	MeP-SPE4	MeP-SPE5	MeP-SPE6
Compound	Group	Recovery rate (%)							Recovery rate (%)					
B-d_8_	Steroid	100 ± 2	81 ± 3	77 ± 4	83 ± 5	84 ± 2	86 ± 3	86 ± 5	88 ± 2	79 ± 11	78 ± 1	56 ± 15	81 ± 5	94 ± 1
18-OHB-d_4_	Steroid	100 ± 2	76 ± 2	78 ± 2	80 ± 0	84 ± 1	85 ± 1	82 ± 2	38 ± 3	39 ± 3	39 ± 6	39 ± 7	36 ± 4	38 ± 2
DOC-d_8_	Steroid	100 ± 4	90 ± 4	85 ± 3	94 ± 3	97 ± 3	98 ± 4	94 ± 6	91 ± 0	75 ± 10	84 ± 8	58 ± 18	83 ± 9	97 ± 1
11-DOF-d_5_	Steroid	100 ± 5	87 ± 3	85 ± 1	90 ± 9	93 ± 5	91 ± 0	89 ± 3	82 ± 3	70 ± 9	74 ± 2	54 ± 14	77 ± 5	85 ± 1
F-d_4_	Steroid	100 ± 3	72 ± 4	72 ± 3	76 ± 2	84 ± 3	83 ± 7	85 ± 4	44 ± 5	36 ± 3	35 ± 4	29 ± 5	34 ± 6	45 ± 9
E-d_8_	Steroid	100 ± 5	85 ± 4	84 ± 1	90 ± 4	89 ± 2	88 ± 1	87 ± 3	58 ± 10	49 ± 5	45 ± 3	35 ± 8	45 ± 3	53 ± 3
T-d_3_	Steroid	100 ± 4	89 ± 1	77 ± 2	91 ± 6	88 ± 2	96 ± 5	92 ± 5	97 ± 4	80 ± 12	90 ± 6	60 ± 20	87 ± 5	105 ± 5
Chol-S-d_7_	Steroid	100 ± 24	19 ± 11	9 ± 9	4 ± 4	2 ± 1	4 ± 4	10 ± 12	24 ± 4	45 ± 13	32 ± 26	26 ± 10	46 ± 11	11 ± 6
CA-d_4_	Bile acid	100 ± 7	78 ± 1	76 ± 4	77 ± 4	72 ± 1	77 ± 3	78 ± 3	73 ± 3	68 ± 11	65 ± 1	40 ± 14	55 ± 8	55 ± 0
CDCA-d_4_	Bile acid	100 ±4	100 ± 4	95 ± 4	93 ± 6	88 ± 6	89 ± 3	92 ± 5	86 ± 3	76 ± 11	73 ± 3	44 ± 16	67 ± 6	66 ± 3
DCA-d_4_	Bile acid	100 ± 3	104 ± 1	98 ± 1	97 ± 4	87 ± 2	91 ± 4	92 ± 4	98 ± 9	91 ± 16	85 ± 9	56 ± 20	89 ± 2	74 ± 3
LCA-d_4_	Bile acid	100 ± 10	141 ± 10	141 ± 15	128 ± 9	110 ± 9	120 ± 1	120 ± 11	124 ± 14	137 ± 32	98 ± 38	69 ± 27	107 ± 9	66 ± 7
GCA-d_4_	Bile acid	100 ± 8	82 ± 2	89 ± 12	76 ± 4	71 ± 1	73 ± 3	74 ± 5	61 ± 4	58 ± 8	51 ± 4	34 ± 10	44 ± 8	44 ± 2
GDCA-d_4_	Bile acid	100 ± 8	96 ± 2	97 ± 6	91 ± 2	84 ± 4	87 ± 1	90 ± 6	89 ± 11	88 ± 12	69 ± 4	43 ± 16	63 ± 9	57 ± 1
GLCA-d_4_	Bile acid	100 ± 8	108 ± 4	134 ± 22	95 ± 7	86 ± 6	89 ± 3	88 ± 3	88 ± 13	85 ± 14	65 ± 10	43 ± 15	64 ± 5	53 ± 2
TCA-d_5_	Bile acid	100 ± 11	87 ± 47	62 ± 46	35 ± 34	12 ± 5	34 ± 46	55 ± 42	32 ± 4	35 ± 7	16 ± 9	6 ± 6	21 ± 6	20 ± 14
TLCA-d_5_	Bile acid	100 ± 5	76 ± 53	54 ± 47	17 ± 22	2 ± 1	20 ± 33	34 ± 48	27 ± 6	27 ± 5	15 ± 1	8 ± 2	16 ± 1	11 ± 1
PGE2-d_4_	PUFA	100 ± 5	72 ± 2	76 ± 5	69 ± 4	68 ± 1	74 ± 1	74 ± 7	62 ± 7	51 ± 6	50 ± 5	29 ± 12	44 ± 13	40 ± 3
PGD2-d_4_	PUFA	100 ± 5	71 ± 2	77 ± 7	72 ± 2	70 ± 2	72 ± 1	76 ± 5	122 ± 18	146 ± 26	126 ± 12	70 ± 28	119 ± 1	93 ± 5
PGF2α-d_4_	PUFA	100 ± 4	65 ± 3	69 ± 5	67 ± 1	68 ± 3	68 ± 2	72 ± 5	49 ± 4	41 ± 9	38 ± 3	21 ± 8	32 ± 11	34 ± 0
TXB2-d_4_	PUFA	100 ± 4	75 ± 1	76 ± 2	75 ± 2	73 ± 1	78 ± 0	79 ± 6	71 ± 6	64 ± 10	38 ± 3	36 ± 13	53 ± 12	55 ± 2
LTD4-d_5_	PUFA	100 ± 9	81 ± 2	82 ± 6	79 ± 4	77 ± 4	68 ± 2	77 ± 8	67 ± 12	67 ± 14	51 ± 10	36 ± 14	51 ± 14	40 ± 0
LTB4-d_4_	PUFA	100 ± 5	84 ± 2	84 ± 3	80 ± 2	81 ± 3	83 ± 3	87 ± 3	79 ± 7	67 ± 14	63 ± 6	40 ± 15	63 ± 8	61 ± 0
15-HETE-d_8_	PUFA	100 ± 12	123 ± 7	109 ± 10	104 ± 2	98 ± 6	100 ± 6	105 ± 5	108 ± 15	105 ± 29	87 ± 19	49 ± 21	84 ± 3	69 ± 3
15-HEPE-d_5_	PUFA	100 ± 6	124 ± 10	114 ± 14	115 ± 7	106 ± 3	108 ± 2	111 ± 4	119 ± 13	98 ± 24	96 ± 8	50 ± 19	87 ± 1	85 ± 3
DHA-d_5_	PUFA	100 ± 17	150 ± 21	110 ± 25	126 ± 12	120 ± 10	124 ± 10	137 ± 2	56 ± 1	86 ± 4	51 ± 33	60 ± 16	59 ± 14	31 ± 6
AA-d_8_	PUFA	100 ± 15	177 ± 16	138 ± 26	160 ± 10	155 ± 3	165 ± 3	169 ± 2	86 ± 6	120 ± 10	76 ± 47	75 ± 22	92 ± 10	48 ± 8
EPA-d_5_	PUFA	100 ± 10	149 ± 12	121 ± 25	126 ± 12	125 ± 6	133 ± 4	140 ± 7	76 ± 5	107 ± 10	64 ± 38	72 ± 23	88 ± 8	49 ± 7
Recovery > 70%		28	26	24	23	23	23	25	17	15	11	2	11	7
RSD < 30%		28	26	26	27	28	26	26	28	27	24	28	28	28

IS, internal standard; MeP, methanol precipitation; RSD, relative standard deviation; SPE, solid-phase extraction.

### Investigation of the effect of dwell time on the sensitivity of LC/MRM measurements

Finally, the relationship between sensitivity and dwell time in LC/MRM measurements was investigated. The sensitivity of IS (testosterone-d_3_) in reference human plasma sample extracts was evaluated by varying the dwell time from 2 to 100 ms while keeping the cycle time fixed at 1 s (supplemental Fig. S4). Comparing dwell times of 2 ms and 100 ms, signal-to-noise ratio improved approx. 2.3 times with the longer dwell time, but had little effect on ion intensity. In this study, a dwell time of 2 ms was used for a large number of compounds to be measured.

### Application to plasma bioactive lipid profiles in healthy human volunteers

Using the optimized method, we analyzed human plasma samples. Samples were obtained from eight healthy volunteers, including five males and three females. All blood samples were collected before meals; female samples were collected in the morning, whereas male samples were collected at three time points: morning, noon, and evening. Detailed sample information is provided in supplemental Table S2.

**Fig. 4:**
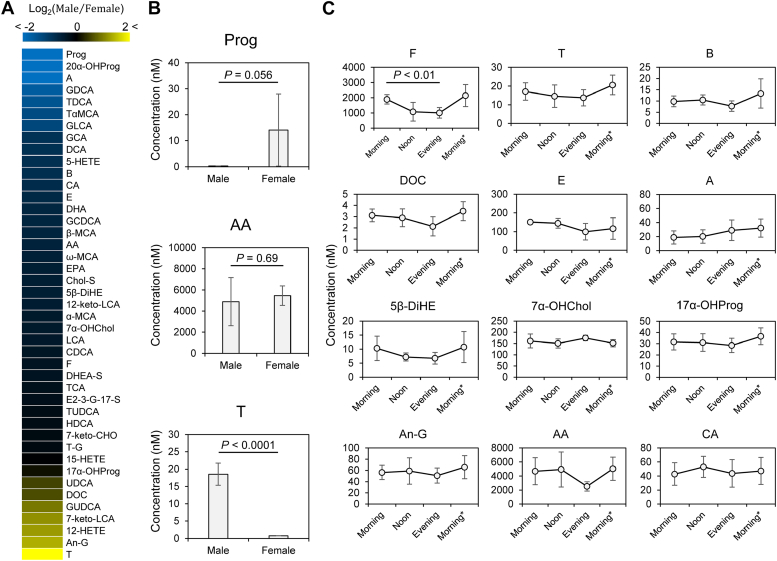
Investigation of sex differences and circadian rhythms using plasma analysis from healthy human volunteers. A: Analysis of sex-related differences. The ratios of detected bioactive lipids in males (n = 5) and females (n = 3) were calculated. Those with a ratio of 1 are shown in black, those with a ratio of 0 (indicating more females) are shown in blue, and those with a ratio of 5 or more (indicating more males) are shown in yellow. B: Bar graph of representative bioactive lipids in the sex-difference analysis. C: Circadian rhythm analysis. Male plasma was collected in the morning (n = 5), noon (n = 5), and evening (n = 4) on certain days, and in the morning (n = 7) on other days. Values are mean ± SD. *P* values were calculated using paired *t* test. 5-HETE, 5S-hydroxy-6E,8Z,11Z,14Z-eicosatetraenoic acid; 5β-DiHE, 5β-dihydrocortisone; 7α-OHChol, 7α-hydroxycholesterol; 7-keto-Chol, 7-ketocholesterol; 7-keto-LCA, 7-ketolithocholic acid; 12-HETE, 12S-hydroxy-5Z,8Z,10E,14Z-eicosatetraenoic acid; 12-keto-LCA, 12-ketolithocholic acid; 15-HETE, 15S-hydroxy-5Z,8Z,11Z,13E-eicosatetraenoic acid; 17α-OHProg, 17α-hydroxyprogesterone; 20α-OHProg, 20α-hydroxyprogesterone; α-MCA, α-muricholic acid; β-MCA, β-muricholic acid; ω-MCA, ω-muricholic acid; A, aldosterone; AA, arachidonic acid 20:4 (n-6); An-G, androsterone glucuronide; and T, testosterone; B, corticosterone; CA, cholic acid; CDCA, chenodeoxycholic acid; Chol-S, cholesterol sulfate; DCA, deoxycholic acid; DHA, docosahexaenoic acid 22:6 (n-3); DHEA-S, dehydroepiandrosterone sulfate; DOC, 11-deoxycorticosterone; E, cortisone; E2-3-G-17-S, β-estradiol 3-(β-D-glucuronide) 17-sulfate; EPA, eicosapentaenoic acid 20:5 (n-3); F, cortisol; GCA, glycocholic acid; GCDCA, glycochenodeoxycholic acid; GDCA, glycodeoxycholic acid; GLCA, glycolithocholic acid; GUDCA, glycoursodeoxycholic acid; HDCA, hyodeoxycholic acid; LCA, lithocholic acid; Prog, progesterone; TαMCA, tauro-α-muricholic acid; TCA, taurocholic acid; TDCA, taurodeoxychloic acid; T-G, testosterone glucuronide; TUDCA, tauroursodeoxycholic acid; UDCA, ursodeoxycholic acid.

A total of 46 bioactive lipids (19 steroids, 20 bile acids, and seven PUFA metabolites) were successfully measured in the human plasma (supplemental Table S10). Sex differences in the measured bioactive lipids were investigated using morning samples. Although metabolites such as arachidonic acid did not show sex differences, the presence of sex-dependent metabolites, including sex hormones such as progesterone in women and testosterone in men, was observed (Fig. 4A, B). The diurnal variation was also examined using male samples collected in the morning, noon, and evening. In addition to cortisol, which has a circadian rhythm, a similar diurnal variation was observed for several steroid hormones (Fig. 4C).

## Discussion

To achieve simultaneous quantitative analysis of bioactive lipids, including steroids, bile acids, and PUFA metabolites in human plasma, it is necessary to develop LC/MS/MS analytical and sample preparation methods using the physicochemical properties of these metabolites as indicators (supplemental Table S1). The MRM mode using QqQMS was used in this study because of its sensitive and selective quantitative performance and reliable monitoring of low concentrations of lipids (19, 20). The MRM/SIM transition settings for 144 bioactive lipids, including 76 steroids, 27 bile acids, and 41 PUFA metabolites, and 28 stable isotopes were performed by optimizing collision energy and other parameters for each compound (supplemental Table S5). The LC separation of bioactive lipids was investigated using four different reversed-phase columns (supplemental Table S6). The log *P*_ow_ values for cholesterol-related metabolites such as hydroxycholesterol, epoxycholesterol, and cholesterol sulfate were >6 (supplemental Table S1). Therefore, a mixture of water–acetonitrile and 2-propanol, a solvent with high elution power, had to be used to elute these compounds retained on the C18 particles. However, due to the high viscosity of 2-propanol, the Inertsil ODS-P column exceeded the usable pressure limit (>50 MPa) and was eliminated as a candidate. The effect of LC analytical conditions on the retention behavior of bioactive lipids was evaluated using three different columns (i.e., Inertsil ODS-4, InertSustain C18, and Inertsil ODS-HL) and two different additives (ammonium acetate and acetic acid). Ammonium acetate was selected as the additive in this study because acetic acid typically produces broad tailing peaks for some compounds under LC/MS/MS analysis conditions using three different columns. Comparison of the three columns showed no significant differences in elution patterns of bioactive lipid molecules or separation performance of isomer pairs (Fig. 1C and supplemental Fig. S1). Consequently, we selected the InertSustain C18 column, which provides stable measurements at the lowest column backpressure, and determined the optimal LC gradient conditions for the analysis of comprehensive bioactive lipids (Fig. 2). Optimization of LC/MS/MS analytical conditions has led to the development of highly sensitive and repeatable analytical method for a wide range of steroids, bile acids, and PUFA metabolites (supplemental Table S5).

To measure numerous trace metabolites in biological samples simultaneously and reproducibly, the evaluation of the sample preparation methods is important. The role of the sample pretreatment methods is to remove impurities from the extracted solution and recover and purify the targeted components with high efficiency. We hypothesized that plasma proteins, hydrophilic metabolites, and highly hydrophobic metabolites such as phospholipids and neutral lipids would interfere with the comprehensive LC/MS/MS measurements of bioactive lipids in human plasma. The sample preparation method was optimized using the removal efficiency of these compounds as an indicator. For protein removal, all three methods tested (MeP, SPE, and MeP-SPE1) showed high removal efficiencies of >97%. Hydrophilic compounds were shown to be effectively removed from human plasma methanol extracts by a washing process with aqueous solvents in SPE (supplemental Fig. S2 and Fig. 3A). In particular, MeP-SPE1 method was found to remove the representative amino acids (serine, glutamic acid, and phenylalanine) with more than 87% efficiency compared to MeP method (Fig. 3B). Similarly, we tested the removal of highly hydrophobic compounds such as phospholipids and neutral lipids from human plasma by MeP-SPE1 (Fig. 3C). When comparing MeP and MeP-SPE1 using the full-scan mode of QqQMS, a decrease in background ions was observed, but the result was not clear enough to discuss. Because the full-scan mode was limited in capturing detailed changes in each purification method of human plasma, LPC, LPE, PC, and TG lipid species, which are the most abundant phospholipids and neutral lipids, were further measured in the MRM mode. The results showed that MeP-SPE1 method removed only approx. 10% of LPCs, but approx. 45% of LPEs and PCs, and >99% of TGs compared to MeP. At least half or most of the lipid classes of LPE, PC, and TG, in addition to proteins and hydrophilic compounds, were removed by MeP-SPE1, suggesting that MeP-SPE1 contributes to reduced ionization suppression and improved analytical stability. However, since MeP-SPE1 method has low removal efficiency for the LPC lipid class, the improvement of pretreatment methods that can selectively remove LPC molecules is a future challenge for the comprehensive observation of bioactive lipids. On the other hand, when SPE cleaning procedures were examined, no significant differences were observed in the removal of hydrophilic compounds or highly hydrophobic compounds among the six SPE procedures studied (supplemental Table S4 and supplemental Fig. S3). Surprisingly, the lack of significant effect of hexane washing, which is conventionally used in solid-phase purification of lipid mediators (32), may suggest that the effect of hexane washing is limited.

Next, the recovery of target bioactive lipids from human plasma samples was evaluated using 28 stable isotope-labeled ISs, including eight steroids, nine bile acids, and 11 PUFA metabolites. Because MeP-SPE1 yielded the highest number of 26 compounds with IS recoveries >70% in the evaluation of plasma-free ISs (Table 1), this protocol was used in this study. Compounds with poor overall recovery were taurocholic acid-d_5_, taurolithocholic acid-d_5_, and cholesterol sulfate-d_7_, which are taurine and sulfate conjugates with sulfo groups in their substructures that are highly acidic (p*K*_a_ of −1.06, −0.84, and −1.36, respectively). In addition, taurocholic acid-d_5_ and taurolithocholic acid-d_5_ showed high relative standard deviations (RSDs), with recovery rates >30%. When evaluating the recovery of the IS without plasma, compounds with sulfo groups as substructures showed poor recovery and repeatability. However, when ISs were added to plasma, the recovery rates tended to be lower than those obtained from ISs without plasma. For example, the recovery rate of cortisol-d_4_ for MeP-SPE1 was 72% for ISs alone but 44% for ISs with plasma. This was attributed to ion suppression caused by the plasma matrix. In addition, when the plasma and IS were extracted together, compounds with poor repeatability without plasma tended to exhibit improved repeatability. For example, the repeatability of taurocholic acid-d_5_ and taurolithocholic acid-d_5_ in MeP-SPE1 was 47% and 53%, respectively, with ISs alone but improved to 4% and 6%, respectively, when extracted with plasma. The phenomenon of changes in elution behavior due to the loose binding of some matrix components to the analytes, as previously suggested by chromatography results (33), may partially explain the observed difference in repeatability between SPE with and without the plasma matrix. The RSDs of IS recovery with the plasma matrix was <30% for all ISs in MeP-SPE1 with a maximum RSD of 18% for prostaglandin D_2_. Therefore, we used MeP-SPE1 for plasma analysis in this study. Although further investigations on extraction methods are needed to improve the recovery rate, the combination of the developed LC/MS/MS and sample preparation methods can provide quantitative values, even for compounds with low recovery rates, by using appropriate ISs for normalization.

We report the accuracy levels of the quantitative values obtained using the proposed method. In LC/MS, the use of the stable isotope dilution method enables the correction of factors affecting quantification, such as extraction efficiency and ionization efficiency, resulting in more accurate quantification (34). However, when the number of targets to be measured exceeds 140, as was the case in this study, preparing stable isotope-labeled ISs for all targets is impractical because of limitations in running costs and commercial availability. The analytical method includes quantification values corrected for stable isotope-labeled ISs corresponding to the target compounds as well as those with similar chemical properties but not necessarily identical to the target compounds. Improving the quantification of all targets will be a future challenge. Here, we reinvestigated the extraction method after expanding the scope of measurement to include steroids, bile acids, and PUFA metabolites. The removal of nontargeted proteins and hydrophilic and highly hydrophobic compounds and the recovery of the targeted compounds were investigated. Consequently, the LC/MS/MS method combined with MeP-SPE1 enabled the measurement of a wide variety of bioactive lipids, including steroids, bile acids, and PUFA metabolites.

Recent improvements in QqQMS scan speed [maximum scan speed: previous QqQMS, approx. 10,000 u/s; and LCMS-8060 QqQMS, 30,000 u/s] have reduced the time required for each MRM event to a few ms. In MRM, dwell time is the time required for the MS detector to count ions. Theoretically, increasing the dwell time can reduce random noise without affecting the intensity of the target ion. In the present study, we confirmed that increasing the dwell time from 2 to 100 ms increased the signal-to-noise ratio by a factor of approx. 2.3, while the ion intensity value remained almost unchanged (supplemental Fig. S4). However, there is a trade-off between increasing dwell time and increasing the number of targets. While a 2.3-fold increase in sensitivity is attractive, the loop time must be kept short to ensure data points. In addition, changing the dwell time for each compound makes normalization by IS more complex because normalization is performed on MRM events with different dwell times. Therefore, the dwell time was set to 2 ms for all compounds in this study. Increasing the dwell time of all targeted compounds using tightly scheduled MRMs is a further study.

Finally, plasma samples from eight healthy volunteers, five males and three females, were analyzed using the optimized method (supplemental Table S9). We observed the presence of sex-dependent metabolites, including progesterone in women and testosterone in men (Fig. 4A, B). This highlights the importance of investigating the presence of sex-differentiating metabolites beyond sex hormones for biomarker discovery. Progesterone showed a large SD, which may be owing to the influence of the menstrual cycle on female hormones. We also examined the diurnal variation using male samples collected in the morning, noon, and evening. Cortisol a metabolite with a circadian rhythm, with high levels in the morning and low levels in the evening (35). Our results captured the circadian rhythm of cortisol, which gradually decreased from morning to evening and returned to high levels the following morning (Fig. 4C). Furthermore, a similar diurnal variation was observed for the other steroid hormones except for aldosterone (Fig. 4C). In addition, arachidonic acid, which is grouped as a PUFA metabolite, tended to have high levels in the morning and at noon, whereas cholic acid, which is grouped as a bile acid, showed high levels at noon (Fig. 4C). In this study, we demonstrated the presence of circadian rhythms in several bioactive lipids, in addition to well-known circadian rhythms such as cortisol. This finding also suggests the importance of considering circadian rhythms in biomarker discovery. These results will provide useful information for future biomarker discovery to improve analysis resolution.

In conclusion, we successfully developed an LC/MS/MS method for the simultaneous quantitative determination of a wide range of bioactive lipids, including steroids, bile acids, and PUFA metabolites. In addition, by effectively combining protein precipitation with methanol and purification with SPE, a sample preparation method suitable for the simultaneous analysis of numerous active lipids in human plasma was developed. Using the developed sample preparation and analysis method, we measured bioactive lipids in the plasma of healthy human volunteers and identified sex differences and circadian rhythms of various bioactive lipids. The newly developed method for measuring bioactive lipids and knowledge gained from the measurement of bioactive lipids in healthy plasma will improve the resolution of biomarker discovery in the future.

## Data availability

All data are presented within the article and Supporting information.

## Supplemental data

This article contains supplemental data.

## Conflict of interest

The author declares that they have no conflicts of interest with the contents of this article.
