# Supplementary material for: Wide-scope targeted analysis of bioactive lipids in human plasma by LC/MS/MS
Source: J Lipid Res. 2023 Dec 20;65(1):100492. doi: 10.1016/j.jlr.2023.100492 (PMC10821590; doi:10.1016/j.jlr.2023.100492)
Supplement: Supplemental Figures [file mmc1.docx]

**Supplemental FIGURES**

**Wide-scope targeted analysis of bioactive lipids in human plasma by LC/MS/MS**

Kohta Nakatani,* Yoshihiro Izumi,^1,^* Hironobu Umakoshi,^†^ Maki Yokomoto-Umakoshi,^†^ Tomoko Nakaji,* Hiroki Kaneko,^†^ Hiroshi Nakao,^†^ Yoshihiro Ogawa,^†^ Kazutaka Ikeda,^§^ Takeshi Bamba^1,^*


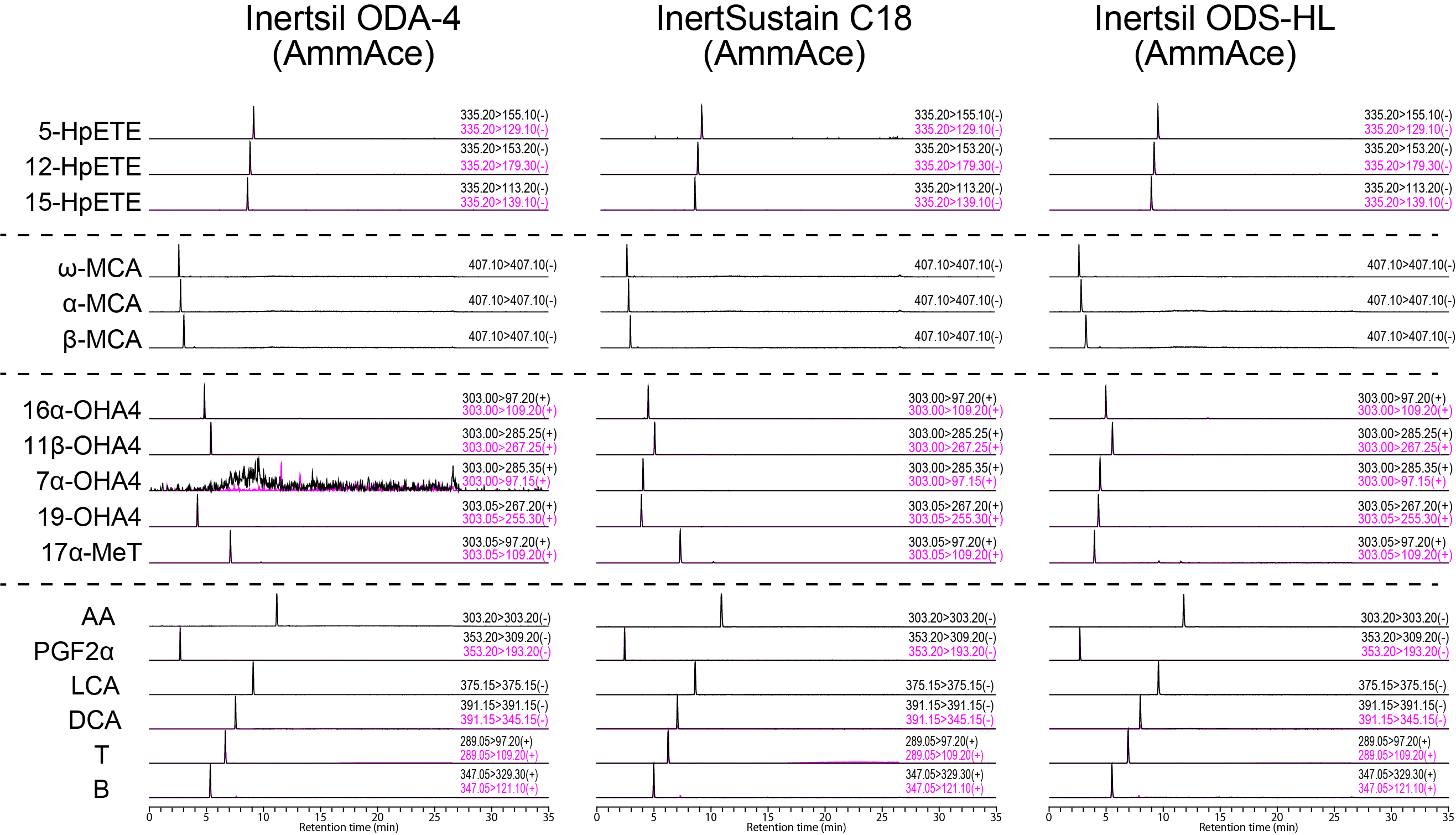


**Supplemental Fig. S1**. LC/MRM chromatograms of representative bioactive lipid molecules, including isomer pairs, using a column of either Inertsil ODS-4, InertSustain C18 or Inertsil ODS-HL under ammonium acetate addition conditions.


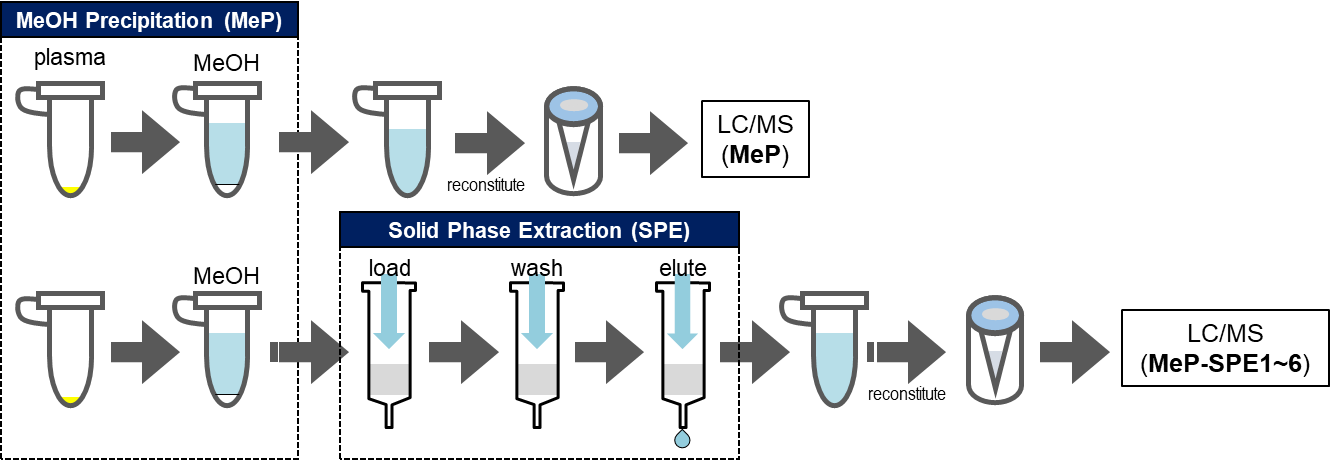


**Supplemental Fig. S2**. Overview of sample preparation procedures to evaluate removal of proteins, hydrophilic compounds, and highly hydrophobic compounds and recovery of targeted bioactive lipids.


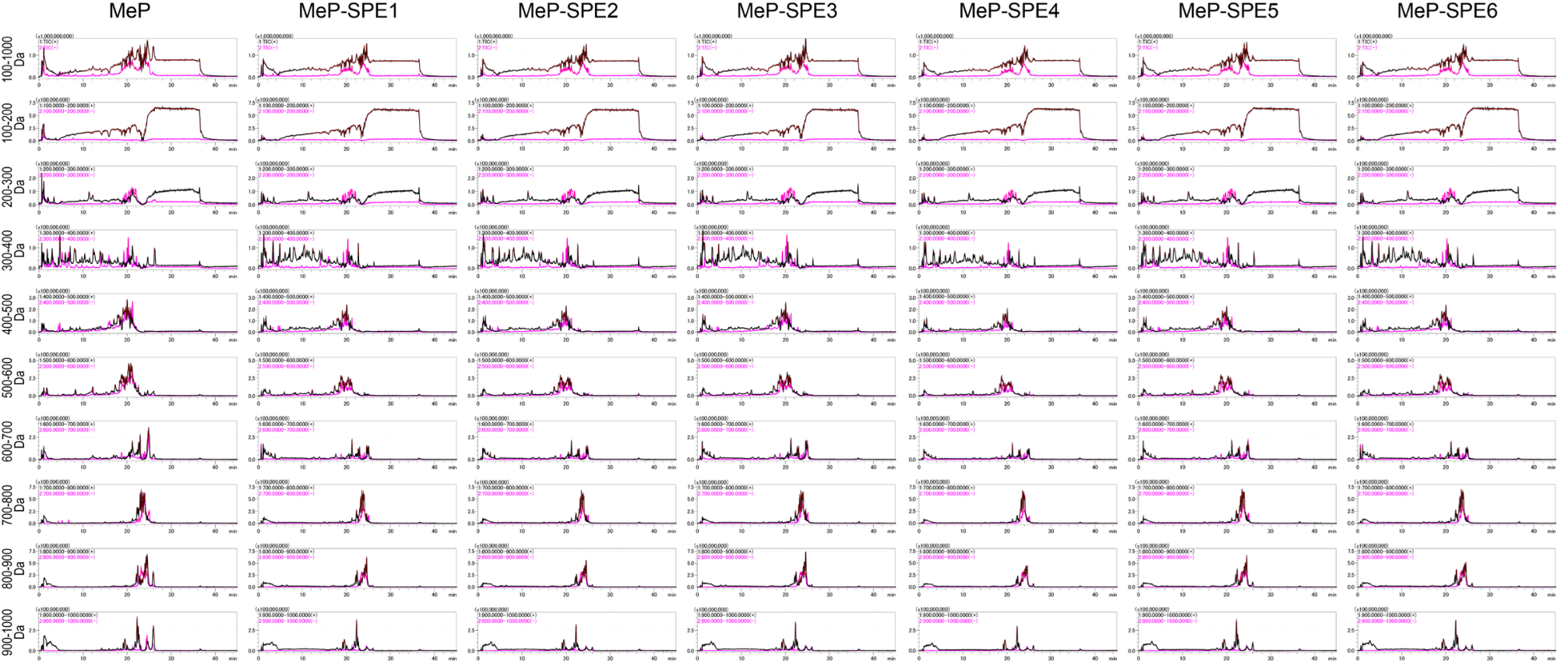


**Supplemental Fig. S3**. LC/MS total ion current chromatograms of human plasma extracts in the positive-ion full scan mode (black) and in the negative-ion full scan mode (pink) using different SPE sample preparation methods.

**
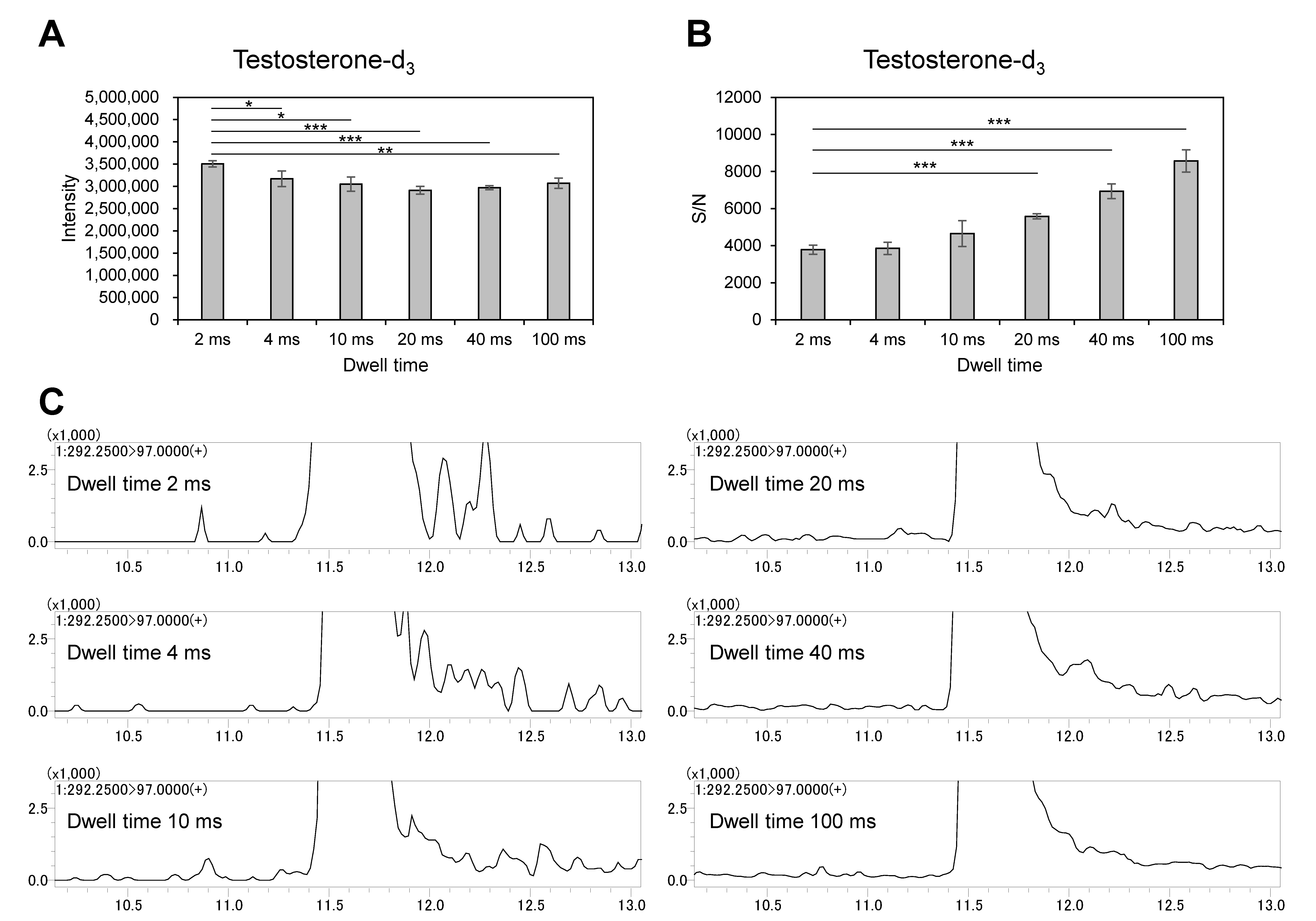
**

**Supplemental Fig. S4**. Evaluation of dwell time on LC/MRM sensitivity in this study.

A: Effect of dwell time on peak intensity. B: Effect of dwell time on S/N. Values are mean ± standard deviation (*n* = 3). *P* values were calculated using Paired *t*-test (**P* < 0.05, ***P* < 0.01, ****P* < 0.001). C: Baseline noise variation of MRM peak of testosterone-d_3_ in human plasma extracts at different dwell times.
