# Supplementary material for: Wide-scope targeted analysis of bioactive lipids in human plasma by LC/MS/MS
Source: J Lipid Res. 2023 Dec 20;65(1):100492. doi: 10.1016/j.jlr.2023.100492 (PMC10821590; doi:10.1016/j.jlr.2023.100492)
Supplement: Supplemental Tables [file mmc2.docx]

**Supplemental TABLES**

**Wide-scope targeted analysis of bioactive lipids in human plasma by LC/MS/MS**

Kohta Nakatani,* Yoshihiro Izumi,^1,^* Hironobu Umakoshi,^†^ Maki Yokomoto-Umakoshi,^†^ Tomoko Nakaji,* Hiroki Kaneko,^†^ Hiroshi Nakao,^†^ Yoshihiro Ogawa,^†^ Kazutaka Ikeda,^§^ Takeshi Bamba^1,^*

**Supplemental Table S1.** Physicochemical properties of steroids, bile acids and PUFA metabolites in the library.

|  | Compound name | Abbreviation | Group | CAS number | HMDB ID | KEGG ID | SMILES | log *P_ow_*(consensus) | p*K*_a_ | p*K*_b_ |
| --- | --- | --- | --- | --- | --- | --- | --- | --- | --- | --- |
|  |  |  |  |  |  |  |  |  |  |  |
| 1 | Estriol-16-glucuronide | E3-16-G | Steroid | 99156-45-3 | - | - | OC(=O)C1OC(OC2CC3C(C2O)(C)CCC2C3CCc3c2ccc(c3)O)C(C(C1O)O)O | 0.81 | 3.46 | -3.35 |
| 2 | 18-Oxocortisol | 18-oxo-F | Steroid | 275816-51-8 | - | - | OCC(=O)C1(O)CCC2C1(C=O)CC(O)C1C2CCC2=CC(=O)CCC12C | 0.70 | 12.42 | -2.85 |
| 3 | β-Estradiol 3-(β-D-glucuronide) 17-sulfate | E2-3-G-17-S | Steroid | 1852-50-2 | HMDB06766 | C05504 | OC(=O)C1OC(Oc2ccc3c(c2)CCC2C3CCC3(C2CCC3OS(=O)(=O)O)C)C(C(C1O)O)O | 1.33 | -1.38 | -3.69 |
| 4 | 18-Hydroxycortisol | 18-OHF | Steroid | - | HMDB06767 | C05503 | OCC(=O)C1(O)CCC2C1(CO)CC(O)C1C2CCC2=CC(=O)CCC12C | 0.58 | 12.51 | -2.80 |
| 5 | 17β-Estradiol-3-glucuronide | E2-3-G | Steroid | 221664-05-7 | - | - | OC1C(O)C(Oc2ccc3c(c2)CCC2C3CCC3(C2CCC3O)C)OC(C1O)C(=O)O | 1.56 | 3.30 | -0.88 |
| 6 | Estrone glucuronide | E1-G | Steroid | 58962-34-8 | HMDB0002886 | C05961 | OC1C(O)C(Oc2ccc3c(c2)CCC2C3CCC3(C2CCC3=O)C)OC(C1O)C(=O)O | 1.59 | 3.30 | -3.69 |
| 7 | 18-Hydroxycorticosterone | 18-OHB | Steroid | 552830-51-0 | HMDB0010410 | - | OCC(=O)C1CCC2C1(CO)CC(O)C1C2CCC2=CC(=O)CCC12C | 1.37 | 13.80 | -2.77 |
| 8 | Testosterone glucuronide | T-G | Steroid | 2479-90-5 | HMDB04483 | C11133 | O=C1CCC2(C(=C1)CCC1C2CCC2(C1CCC2OC1OC(C(=O)O)C(C(C1O)O)O)C)C | 1.67 | 3.63 | -3.69 |
| 9 | Taurocholic acid | TCA | Bile acid | 1180-25-2 | HMDB03193 | C11134 | OC1CCC2(C(C1)CC(C1C2CC(O)C2(C1CCC2C(CCC(=O)NCCS(=O)(=O)O)C)C)O)C | 1.97 | -1.06 | 0.28 |
| 10 | 11β,17α,21-Trihydroxypregnenolone | 11β,17α,21-(OH)3Preg | Steroid | 2410-60-8 | HMDB0000332 | - | OCC(=O)C1(O)CCC2C1(C)CC(O)C1C2CC=C2C1(C)CCC(C2)O | 1.47 | 12.58 | -1.40 |
| 11 | Aldosterone | A | Steroid | 86002-90-6 | HMDB0000418 | - | OCC(=O)C1CCC2C1(C=O)CC(O)C1C2CCC2=CC(=O)CCC12C | 1.50 | 13.82 | -2.85 |
| 12 | Androsterone glucuronide | An-G | Steroid | 54397-85-2 | HMDB0003252 | C05963 | OC(=O)C1OC(OC2CCC3(C(C2)CCC2C3CCC3(C2CCC3=O)C)C)C(C(C1O)O)O | 1.76 | 3.47 | -3.69 |
| 13 | Etiocholanolone glucuronide | Et-G | Steroid | 652-69-7 | HMDB11653 | C04518 | OC(=O)C1OC(OC2CCC3(C(C2)CCC2C3CCC3(C2CCC3=O)C)C)C(C(C1O)O)O | 1.76 | 3.47 | -3.69 |
| 14 | Cortisol | F | Steroid | 71902-47-1 | - | - | OCC(=O)C1(O)CCC2C1(C)CC(O)C1C2CCC2=CC(=O)CCC12C | 1.54 | 12.58 | -2.85 |
| 15 | Cortisone | E | Steroid | 2260905-08-4 | - | - | OCC(=O)C1(O)CCC2C1(C)CC(=O)C1C2CCC2=CC(=O)CCC12C | 1.52 | 12.58 | -3.33 |
| 16 | 5β-Dihydrocortisol | 5β-DiHF | Steroid | 25696-60-0 | HMDB0000932 | - | OCC(=O)C1(O)CCC2C1(C)CC(O)C1C2CCC2C1(C)CCC(=O)C2 | 1.54 | 12.58 | -2.84 |
| 17 | Tauro-β-muricholic acid | TβMCA | Bile acid | 551-11-1 | HMDB0001139 | C00639 | OC1CCC2(C(C1)C(O)C(C1C2CCC2(C1CCC2C(CCC(=O)NCCS(=O)(=O)O)C)C)O)C | 2.04 | -0.98 | 0.08 |
| 18 | Tauro-α-muricholic acid | TαMCA | Bile acid | 6830-03-1 | HMDB0000364 | C17727 | OC1CCC2(C(C1)C(O)C(C1C2CCC2(C1CCC2C(CCC(=O)NCCS(=O)(=O)O)C)C)O)C | 2.04 | -0.98 | 0.08 |
| 19 | 17α,21-Dihydroxy-5β-pregnane-3,11,20-trione | 5β-DiHE | Steroid | 561-65-9 | HMDB00319 | C01124 | OCC(=O)C1(O)CCC2C1(C)CC(=O)C1C2CCC2C1(C)CCC(=O)C2 | 1.52 | 12.58 | -3.33 |
| 20 | 6-keto-Prostaglandin F1α | 6-keto-PGF1α | PUFA metabolite | 363-24-6 | HMDB0001220 | C00584 | CCCCCC(C=CC1C(O)CC(C1CC(=O)CCCCC(=O)O)O)O | 1.91 | 4.14 | -1.63 |
| 21 | (3Z)-5-[(1S,2R,3R,5S)-3,5-Dihydroxy-2-[(1E,3S)-3-hydroxy-1-octen-1-yl]cyclopentyl]-3-pentenoic acid | 2,3-dinor-8iso-PGF2α | PUFA metabolite | 42935-17-1 | HMDB0001381 | C00427 | CCCCCC(C=CC1C(O)CC(C1CC=CCC(=O)O)O)O | 2.02 | 4.41 | -1.63 |
| 22 | Leukotriene C4 | LTC4 | PUFA metabolite | 50-27-1 | HMDB00153 | C05141 | CCCCCC=CCC=CC=CC=CC(C(CCCC(=O)O)O)SCC(C(=O)NCC(=O)O)NC(=O)CCC(C(=O)O)N | 1.65 | 1.80 | 9.31 |
| 23 | Glycocholic acid | GCA | Bile acid | 35121-78-9 | HMDB0001335 | C01312 | OC1CCC2(C(C1)CC(C1C2CC(O)C2(C1CCC2C(CCC(=O)NCC(=O)O)C)C)O)C | 2.40 | 3.77 | -0.04 |
| 24 | Corticosterone | B | Steroid | 52-39-1 | HMDB00037 | C01780 | OCC(=O)C1CCC2C1(C)CC(O)C1C2CCC2=CC(=O)CCC12C | 2.34 | 13.86 | -0.26 |
| 25 | Taurodeoxychloic acid | TDCA | Bile acid | 2393-58-0 | HMDB0000506 | - | OC1CCC2(C(C1)CCC1C2CC(O)C2(C1CCC2C(CCC(=O)NCCS(=O)(=O)O)C)C)C | 2.95 | -0.94 | 0.22 |
| 26 | Estriol | E3 | Steroid | - | HMDB06760 | C05489 | Oc1ccc2c(c1)CCC1C2CCC2(C1CC(C2O)O)C | 2.74 | 10.33 | -3.16 |
| 27 | 21-Deoxycortisol | 21-DOF | Steroid | 2141-17-5 | HMDB03956 | C05291 | O=C1CCC2(C(=C1)CCC1C2C(O)CC2(C1CCC2(O)C(=O)C)C)C | 2.30 | 12.69 | -2.85 |
| 28 | 11-Dehydrocorticosterone | 11-DHB | Steroid | 745-65-3 | HMDB0001442 | C04741 | OCC(=O)C1CCC2C1(C)CC(=O)C1C2CCC2=CC(=O)CCC12C | 2.32 | 13.86 | -3.30 |
| 29 | Thromboxane B2 | TXB2 | PUFA metabolite | 41598-07-6 | HMDB0001403 | C00696 | CCCCCC(C=CC1OC(O)CC(C1CC=CCCCC(=O)O)O)O | 2.54 | 4.27 | -2.85 |
| 30 | Tetrahydrocorticosterone | THB | Steroid | 2126-37-6 | HMDB06769 | C05294 | OCC(=O)C1CCC2C1(C)CC(O)C1C2CCC2C1(C)CCC(C2)O | 2.42 | 13.86 | -0.25 |
| 31 | 7α-Hydroxyandrostenedione | 7α-OHA4 | Steroid | 2393-59-1 | HMDB0000415 | C17726 | O=C1CCC2(C(=C1)CC(C1C2CCC2(C1CCC2=O)C)O)C | 2.65 | 17.14 | -0.61 |
| 32 | 11β-Hydroxyandrostenedione | 11β-OHA4 | Steroid | 3602--09-3 | HMDB04484 | C11136 | O=C1CCC2(C(=C1)CCC1C2C(O)CC2(C1CCC2=O)C)C | 2.65 | 14.88 | -2.85 |
| 33 | 17α,21-Dihydroxypregnenolone | 17,21-(OH)2Preg | Steroid | 1852-43-3 | HMDB02829 | C11135 | OCC(=O)C1(O)CCC2C1(C)CCC1C2CC=C2C1(C)CCC(C2)O | 2.47 | 12.59 | -1.40 |
| 34 | 7α-Hydroxydehydroepiandrosterone | 7α-OHDHEA | Steroid | 481-97-0 | HMDB01425 | C02538 | OC1CCC2(C(=CC(C3C2CCC2(C3CCC2=O)C)O)C1)C | 2.63 | 18.20 | -0.81 |
| 35 | 3α,21-Dihydroxy-5β-pregnane-11,20-dione | THA | Steroid | 82864-77-5 | HMDB0002294 | - | OCC(=O)C1CCC2C1(C)CC(=O)C1C2CCC2C1(C)CCC(C2)O | 2.39 | 13.86 | -1.36 |
| 36 | 16α-Hydroxyestrone | 16α-OHE1 | Steroid | 98049-69-5 | HMDB0005082 | C06315 | Oc1ccc2c(c1)CCC1C2CCC2(C1CC(C2=O)O)C | 2.86 | 10.33 | -3.51 |
| 37 | 19-Hydroxyandrostenedione | 19-OHA4 | Steroid | 916888-47-6 | - | - | OCC12CCC(=O)C=C1CCC1C2CCC2(C1CCC2=O)C | 2.68 | 18.71 | -0.97 |
| 38 | Taurohyodeoxycholic acid | THDCA | Bile acid | 51982-36-6 | HMDB0003235 | C05956 | OC1CCC2(C(C1)C(O)CC1C2CCC2(C1CCC2C(CCC(=O)NCCS(=O)(=O)O)C)C)C | 2.93 | -0.98 | 0.08 |
| 39 | 7α-Hydroxytestosterone | 7α-OHT | Steroid | 510-64-5 | HMDB03955 | C05290 | O=C1CCC2(C(=C1)CC(C1C2CCC2(C1CCC2O)C)O)C | 2.63 | 17.14 | -0.44 |
| 40 | 11β-Hydroxytestosterone | 11β-OHT | Steroid | 53-06-5 | HMDB02802 | C00762 | O=C1CCC2(C(=C1)CCC1C2C(O)CC2(C1CCC2O)C)C | 2.63 | 14.48 | -2.85 |
| 41 | 11β,21-Dihydroxy-5β-pregnane-3,20-dione | 5β-DiHB | Steroid | 50-23-7 | HMDB00063 | C00735 | OCC(=O)C1CCC2C1(C)CC(O)C1C2CCC2C1(C)CCC(=O)C2 | 2.34 | 13.86 | -0.29 |
| 42 | 19-Hydroxytestosterone | 19-OHT | Steroid | 64480-66-6 | HMDB0000708 | - | OCC12CCC(=O)C=C1CCC1C2CCC2(C1CCC2O)C | 2.65 | 18.74 | -0.62 |
| 43 | 11-Deoxycortisol | 11-DOF | Steroid | 53-00-9 | HMDB04611 | C18045 | OCC(=O)C1(O)CCC2C1(C)CCC1C2CCC2=CC(=O)CCC12C | 2.53 | 12.59 | -3.33 |
| 44 | Prostaglandin D3 | PGD3 | PUFA metabolite | 13042-33-6 | - | - | CCC=CCC(C=CC1C(=O)CC(C1CC=CCCCC(=O)O)O)O | 2.55 | 4.40 | -1.64 |
| 45 | 16α-Hydroxyandrostenedione | 16α-OHA4 | Steroid | 62-84-0 | HMDB06771 | C05296 | O=C1CCC2(C(=C1)CCC1C2CCC2(C1CC(C2=O)O)C)C | 2.81 | 13.38 | -3.51 |
| 46 | Adrenosterone | 11-keto-A4 | Steroid | 651-48-9 | HMDB01032 | C04555 | O=C1CCC2(C(=C1)CCC1C2C(=O)CC2(C1CCC2=O)C)C | 2.63 | 17.99 | -4.87 |
| 47 | 16α-Hydroxydehydroisoandrosterone | 16α-OHDHEA | Steroid | 966-06-3 | HMDB00313 | C05300 | OC1CCC2(C(=CCC3C2CCC2(C3CC(C2=O)O)C)C1)C | 2.74 | 13.38 | -1.40 |
| 48 | Estrone sulfate | E1-S | Steroid | 475-31-0 | HMDB0000138 | C01921 | O=C1CCC2C1(C)CCC1C2CCc2c1ccc(c2)OS(=O)(=O)O | 3.24 | -1.75 | -7.48 |
| 49 | Tauroursodeoxycholic acid | TUDCA | Bile acid | 14605-22-2 | HMDB0000874 | - | OC1CCC2(C(C1)CC(C1C2CCC2(C1CCC2C(CCC(=O)NCCS(=O)(=O)O)C)C)O)C | 2.93 | -0.99 | 0.18 |
| 50 | Taurochenodeoxycholic acid | TCDCA | Bile acid | 2958-04-5 | - | - | OC1CCC2(C(C1)CC(C1C2CCC2(C1CCC2C(CCC(=O)NCCS(=O)(=O)O)C)C)O)C | 2.93 | -0.99 | 0.18 |
| 51 | Cholic acid | CA | Bile acid | 872993-05-0 | HMDB0003733 | - | OC1CCC2(C(C1)CC(C1C2CC(O)C2(C1CCC2C(CCC(=O)O)C)C)O)C | 3.21 | 4.48 | -0.16 |
| 52 | Prostaglandin F2α | PGF2α | PUFA metabolite | 63-02-5 | HMDB06774 | C05140 | CCCCCC(C=CC1C(O)CC(C1CC=CCCCC(=O)O)O)O | 2.74 | 4.36 | -1.63 |
| 53 | ω-Muricholic acid | ω-MCA | Bile acid | 81-24-3 | HMDB0000036 | - | OC1CCC2(C(C1)C(O)C(C1C2CCC2(C1CCC2C(CCC(=O)O)C)C)O)C | 3.27 | 4.60 | -2.69 |
| 54 | α-Muricholic acid | α-MCA | Bile acid | 72-23-1 | HMDB04029 | C05490 | OC1CCC2(C(C1)C(O)C(C1C2CCC2(C1CCC2C(CCC(=O)O)C)C)O)C | 3.27 | 4.60 | -2.69 |
| 55 | β-Muricholic acid | β-MCA | Bile acid | 1816-85-9 | HMDB12533 | C18075 | OC1CCC2(C(C1)C(O)C(C1C2CCC2(C1CCC2C(CCC(=O)O)C)C)O)C | 3.27 | 4.60 | -2.69 |
| 56 | 2-Hydroxyestrone | 2-OHE1 | Steroid | 1232-73-1 | HMDB00352 | C05139 | O=C1CCC2C1(C)CCC1C2CCc2c1cc(O)c(c2)O | 3.34 | 9.67 | -6.25 |
| 57 | Dehydroepiandrosterone sulfate | DHEA-S | Steroid | 89663-86-5/171030-11-8 | HMDB0004385/HMDB0012587 | C06314 | O=C1CCC2C1(C)CCC1C2CC=C2C1(C)CCC(C2)OS(=O)(=O)O | 3.28 | -1.36 | -7.48 |
| 58 | Resolvin E1 | RvE1 | PUFA metabolite | 13345-50-1 | HMDB0002752 | C05953 | CCC(C=CC=CCC(C=CC=CC=CC(CCCC(=O)O)O)O)O | 2.64 | 4.65 | -1.11 |
| 59 | Glycohyodeoxycholic acid | GHDCA | Bile acid | 382-45-6 | HMDB06772 | C05285 | OC1CCC2(C(C1)C(O)CC1C2CCC2(C1CCC2C(CCC(=O)NCC(=O)O)C)C)C | 3.36 | 3.77 | -0.66 |
| 60 | Prostaglandin D2 | PGD2 | PUFA metabolite | 1482-50-4 | - | C05471 | CCCCCC(C=CC1C(=O)CC(C1CC=CCCCC(=O)O)O)O | 2.80 | 4.40 | -1.60 |
| 61 | Prostaglandin E2 | PGE2 | PUFA metabolite | 13367-85-6 | HMDB0004236 | C05954 | CCCCCC(C=CC1C(O)CC(=O)C1CC=CCCCC(=O)O)O | 2.80 | 4.30 | -1.63 |
| 62 | Glycodeoxycholic acid | GDCA | Bile acid | 72025-60-6 | HMDB0001198 | C02166 | OC1CCC2(C(C1)CCC1C2CC(O)C2(C1CCC2C(CCC(=O)NCC(=O)O)C)C)C | 3.38 | 3.77 | -0.18 |
| 63 | 2-Hydroxyestradiol | 2-OHE2 | Steroid | 382-44-5 | HMDB06773 | C05284 | OC1CCC2C1(C)CCC1C2CCc2c1cc(O)c(c2)O | 3.31 | 9.67 | -0.88 |
| 64 | Leukotriene D4 | LTD4 | PUFA metabolite | 81-25-4 | HMDB0000619 | C00695 | CCCCCC=CCC=CC=CC=CC(C(CCCC(=O)O)O)SCC(C(=O)NCC(=O)O)N | 3.11 | 3.29 | 8.05 |
| 65 | Prostaglandin I2 | PGI2 | PUFA metabolite | 68-54-2 | HMDB06758 | C05469 | CCCCCC(C=CC1C(O)CC2C1CC(=CCCCC(=O)O)O2)O | 2.77 | 4.43 | -1.63 |
| 66 | Glycoursodeoxycholic acid | GUDCA | Bile acid | 641-77-0 | HMDB04030 | C05497 | OC1CCC2(C(C1)CC(C1C2CCC2(C1CCC2C(CCC(=O)NCC(=O)O)C)C)O)C | 3.36 | 3.77 | -0.29 |
| 67 | Glycochenodeoxycholic acid | GCDCA | Bile acid | 911-40-0 | HMDB0000391 | - | OC1CCC2(C(C1)CC(C1C2CCC2(C1CCC2C(CCC(=O)NCC(=O)O)C)C)O)C | 3.36 | 3.77 | -0.29 |
| 68 | 11β-Hydroxyprogesterone | 11β-OHProg | Steroid | 50-22-6 | HMDB01547 | C02140 | O=C1CCC2(C(=C1)CCC1C2C(O)CC2(C1CCC2C(=O)C)C)C | 3.11 | 18.88 | -0.26 |
| 69 | Prostaglandin K2 | PGK2 | PUFA metabolite | 1167-48-2 | HMDB06762 | C05487 | CCCCCC(C=CC1C(=O)CC(=O)C1CC=CCCCC(=O)O)O | 2.88 | 4.35 | -1.60 |
| 70 | Leukotriene E4 | LTE4 | PUFA metabolite | 128-13-2 | HMDB0000946 | C07880 | CCCCCC=CCC=CC=CC=CC(C(CCCC(=O)O)O)SCC(C(=O)O)N | 3.17 | 2.39 | 9.13 |
| 71 | 7-ketodeoxycholic acid | 7-keto-DCA | Bile acid | 362-05-0 | HMDB0000338 | C05301 | OC1CCC2(C(C1)CC(=O)C1C2CC(O)C2(C1CCC2C(CCC(=O)O)C)C)C | 3.18 | 4.44 | -0.33 |
| 72 | 17α,20α-Dihydroxypregn-4-en-3-one | 17,20α-(OH)2Prog | Steroid | 152-58-9 | HMDB00015 | C05488 | O=C1CCC2(C(=C1)CCC1C2CCC2(C1CCC2(O)C(O)C)C)C | 3.32 | 13.42 | -3.13 |
| 73 | Prostaglandin E1 | PGE1 | PUFA metabolite | 83-49-8 | HMDB0000733 | - | CCCCCC(C=CC1C(O)CC(=O)C1CCCCCCC(=O)O)O | 3.05 | 4.35 | -1.63 |
| 74 | 5(S),14(R)-Lipoxin B4 | LXB4 | PUFA metabolite | 1025684-60-9 | - | - | CCCCCC(C(C=CC=CC=CC=CC(CCCC(=O)O)O)O)O | 2.93 | 4.65 | -1.55 |
| 75 | 5(S),6(R)-Lipoxin A4/15-epi-Lipoxin A4 | LXA4/15-epi-LXA4 | PUFA metabolite | - | HMDB60424 | C18038 | CCCCCC(C=CC=CC=CC=CC(C(CCCC(=O)O)O)O)O | 2.93 | 4.48 | -1.58 |
| 76 | 7α-Hydroxypregnenolone | 7α-OHPreg | Steroid | 362-06-1 | HMDB00343 | C05298 | OC1CCC2(C(=CCC3C2CCC2(C3CCC2(O)C(=O)C)C)C1)C | 3.23 | 12.70 | -1.40 |
| 77 | 17α-Hydroxypregnenolone | 17α-OHPreg | Steroid | 4651-67-6 | HMDB0000467 | - | OC1CCC2(C(=CCC3C2CCC2(C3CCC2(O)C(=O)C)C)C1)C | 3.23 | 12.70 | -1.40 |
| 78 | 21-Hydroxypregnenolone | 21-OHPreg | Steroid | 68-42-8 | HMDB00268 | C05476 | OCC(=O)C1CCC2C1(C)CCC1C2CC=C2C1(C)CCC(C2)O | 3.28 | 13.86 | -1.40 |
| 79 | 11-Deoxycorticosterone | DOC | Steroid | 566-03-0 | HMDB06755 | C05478 | OCC(=O)C1CCC2C1(C)CCC1C2CCC2=CC(=O)CCC12C | 3.34 | 13.86 | -3.30 |
| 80 | Estrone | E1 | Steroid | 1247-64-9 | HMDB00774 | C18044 | Oc1ccc2c(c1)CCC1C2CCC2(C1CCC2=O)C | 3.71 | 10.33 | -5.45 |
| 81 | 2-Methoxyestrone | 2-ME1 | Steroid | 73836-78-9 | HMDB0003080 | C05951 | COc1cc2c(cc1O)CCC1C2CCC2(C1CCC2=O)C | 3.60 | 10.29 | -4.88 |
| 82 | 17α-Hydroxyprogesterone | 17α-OHProg | Steroid | 5130-29-0 | HMDB0000328 | - | O=C1CCC2(C(=C1)CCC1C2CCC2(C1CCC2(O)C(=O)C)C)C | 3.30 | 12.70 | -3.80 |
| 83 | Estradiol | E2 | Steroid | 640-79-9 | HMDB0000637 | C05466 | Oc1ccc2c(c1)CCC1C2CCC2(C1CCC2O)C | 3.69 | 10.33 | -0.88 |
| 84 | Pregnenolone sulfate | Preg-S | Steroid | 871826-47-0 | - | - | CC(=O)C1CCC2C1(C)CCC1C2CC=C2C1(C)CCC(C2)OS(=O)(=O)O | 3.74 | -1.36 | -7.36 |
| 85 | Androstenedione | A4 | Steroid | 660430-03-5 | HMDB0003689 | - | O=C1CCC2(C(=C1)CCC1C2CCC2(C1CCC2=O)C)C | 3.66 | 19.03 | -4.82 |
| 86 | Dehydroepiandrosterone | DHEA | Steroid | 566-01-8 | HMDB06757 | C05475 | OC1CCC2(C(=CCC3C2CCC2(C3CCC2=O)C)C1)C | 3.59 | 18.20 | -1.40 |
| 87 | Resolvin D2 | RvD2 | PUFA metabolite | 516-35-8 | HMDB0000951 | C05465 | CCC=CCC(C(C=CC=CC=CC=CC(CC=CCCC(=O)O)O)O)O | 3.14 | 4.64 | -1.61 |
| 88 | Testosterone | T | Steroid | 1268720-28-0 | - | - | O=C1CCC2(C(=C1)CCC1C2CCC2(C1CCC2O)C)C | 3.64 | 19.09 | -0.88 |
| 89 | 5β-Dihydrodeoxycorticosterone | 5β-DiHDOC | Steroid | 474-74-8 | HMDB0000698 | C15557 | OCC(=O)C1CCC2C1(C)CCC1C2CCC2C1(C)CCC(=O)C2 | 3.35 | 13.86 | -3.30 |
| 90 | 5α-Dihydrodeoxycorticosterone | 5α-DiHDOC | Steroid | 50-28-2 | HMDB00151 | C00951 | OCC(=O)C1CCC2C1(C)CCC1C2CCC2C1(C)CCC(=O)C2 | 3.35 | 13.86 | -3.30 |
| 91 | Resolvin D4 | RvD4 | PUFA metabolite | 64-85-7 | HMDB00016 | C03205 | CCC=CCC(C=CC=CCC=CC=CC=CC(C(CCC(=O)O)O)O)O | 3.14 | 4.45 | -1.61 |
| 92 | Resolvin D3 | RvD3 | PUFA metabolite | 516-50-7 | HMDB0000896 | - | CCC=CCC(C=CC=CCC(C=CC=CC=CC(CCC(=O)O)O)O)O | 3.10 | 4.61 | -1.29 |
| 93 | Prostaglandin G2 | PGG2 | PUFA metabolite | 58-22-0 | HMDB00234 | C00535 | CCCCCC(C=CC1C2OOC(C1CC=CCCCC(=O)O)C2)OO | 4.24 | 4.36 | -4.24 |
| 94 | Resolvin D1 | RvD1 | PUFA metabolite | 75715-89-8 | HMDB0002200 | C05952 | CCC=CCC(C=CC=CC=CC=CC(C(CC=CCCC(=O)O)O)O)O | 3.14 | 4.47 | -1.61 |
| 95 | Hyodeoxycholic acid | HDCA | Bile acid | 71160-24-2 | HMDB0001085 | - | OC1CCC2(C(C1)C(O)CC1C2CCC2(C1CCC2C(CCC(=O)O)C)C)C | 4.18 | 4.79 | -2.69 |
| 96 | Etiocholanolone | Et | Steroid | 63-05-8 | HMDB00053 | C00280 | OC1CCC2(C(C1)CCC1C2CCC2(C1CCC2=O)C)C | 3.74 | 18.30 | -1.36 |
| 97 | Androsterone | An | Steroid | 600-57-7 | HMDB04031 | C05498 | OC1CCC2(C(C1)CCC1C2CCC2(C1CCC2=O)C)C | 3.74 | 18.30 | -1.36 |
| 98 | Prostaglandin H2 | PGH2 | PUFA metabolite | 1164-98-3 | HMDB04026 | C05485 | CCCCCC(C=CC1C2OOC(C1CC=CCCCC(=O)O)C2)O | 3.92 | 4.36 | -1.63 |
| 99 | 5β-Dihydrotestosterone/Dihydrotestosterone | 5β-DHT/DHT | Steroid | 53-16-7 | HMDB04449 | C00468 | O=C1CCC2(C(C1)CCC1C2CCC2(C1CCC2O)C)C | 3.64 | 19.38 | -0.88 |
| 100 | Ursodeoxycholic acid | UDCA | Bile acid | 362-08-3 | HMDB00010 | C05299 | OC1CCC2(C(C1)CC(C1C2CCC2(C1CCC2C(CCC(=O)O)C)C)O)C | 4.18 | 4.60 | -0.54 |
| 101 | Chenodeoxycholic acid | CDCA | Bile acid | 387-79-1 | HMDB00363 | C05138 | OC1CCC2(C(C1)CC(C1C2CCC2(C1CCC2C(CCC(=O)O)C)C)O)C | 4.18 | 4.60 | -0.54 |
| 102 | Prostaglandin B2 | PGB2 | PUFA metabolite | 68-96-2 | HMDB00374 | C01176 | CCCCCC(C=CC1=C(CC=CCCCC(=O)O)C(=O)CC1)O | 3.80 | 4.25 | -1.59 |
| 103 | Prostaglandin A2 | PGA2 | PUFA metabolite | 58-18-4 | HMDB15655 | - | CCCCCC(C=CC1C=CC(=O)C1CC=CCCCC(=O)O)O | 3.64 | 4.40 | -1.60 |
| 104 | Taurolithocholic acid | TLCA | Bile acid | 53-43-0 | HMDB00077 | C01227 | OC1CCC2(C(C1)CCC1C2CCC2(C1CCC2C(CCC(=O)NCCS(=O)(=O)O)C)C)C | 3.92 | -0.84 | 0.10 |
| 105 | Etiocholanedione/5α-Androstanedione | ED/AD | Steroid | 474-25-9/15357-34-3 | HMDB0000518/HMDB0000514 | C02528 | O=C1CCC2(C(C1)CCC1C2CCC2(C1CCC2=O)C)C | 3.66 | 19.78 | -7.11 |
| 106 | 7-Ketolithocholic acid | 7-keto-LCA | Bile acid | 83-44-3 | HMDB0000626 | C04483 | OC1CCC2(C(C1)CC(=O)C1C2CCC2(C1CCC2C(CCC(=O)O)C)C)C | 4.15 | 4.56 | -1.33 |
| 107 | Deoxycholic acid | DCA | Bile acid | 141110-17-0 | - | - | OC1CCC2(C(C1)CCC1C2CC(O)C2(C1CCC2C(CCC(=O)O)C)C)C | 4.20 | 4.65 | -0.35 |
| 108 | Methyltestosterone | 17α-MeT | Steroid | 474-74-8 | HMDB0000698 | C15557 | O=C1CCC2(C(=C1)CCC1C2CCC2(C1CCC2(C)O)C)C | 3.91 | 19.09 | -0.53 |
| 109 | Glycolithocholic acid | GLCA | Bile acid | 516-90-5 | HMDB0000722 | C02592 | OC1CCC2(C(C1)CCC1C2CCC2(C1CCC2C(CCC(=O)NCC(=O)O)C)C)C | 4.35 | 3.77 | -0.58 |
| 110 | Pregnenolone | Preg | Steroid | 88852-33-9 | - | - | OC1CCC2(C(=CCC3C2CCC2(C3CCC2C(=O)C)C)C1)C | 4.05 | 18.20 | -1.40 |
| 111 | Leukotriene B4 | LTB4 | PUFA metabolite | 145-14-2 | HMDB03069 | C04042 | CCCCCC=CCC(C=CC=CC=CC(CCCC(=O)O)O)O | 3.86 | 4.65 | -1.27 |
| 112 | 12-Ketolithocholic acid | 12-keto-LCA | Bile acid | 53-42-9 | HMDB00490 | C04373 | OC1CCC2(C(C1)CCC1C2CC(=O)C2(C1CCC2C(CCC(=O)O)C)C)C | 4.22 | 4.56 | -1.36 |
| 113 | Progesterone | Prog | Steroid | 57-83-0 | HMDB01830 | C00410 | O=C1CCC2(C(=C1)CCC1C2CCC2(C1CCC2C(=O)C)C)C | 4.12 | 18.92 | -4.82 |
| 114 | 20α-Hydroxyprogesterone | 20α-OHProg | Steroid | 53-41-8 | HMDB00031 | C00523 | O=C1CCC2(C(=C1)CCC1C2CCC2(C1CCC2C(O)C)C)C | 4.19 | 19.00 | -1.05 |
| 115 | 5β-Dihydroprogesterone/5α-Dihydroprogesterone | 5β-DiHProg/5α-DiHProg | Steroid | 83952-40-3 | - | - | O=C1CCC2(C(C1)CCC1C2CCC2(C1CCC2C(=O)C)C)C | 4.12 | 19.34 | -7.05 |
| 116 | Maresin-1 | Maresin-1 | PUFA metabolite | 90780-52-2 | HMDB0010213 | - | CCC=CCC=CCC(C=CC=CC=CC(CC=CCCC(=O)O)O)O | 4.07 | 4.64 | -1.31 |
| 117 | 10(S),17(S)-dihydroxy-4Z,7Z,11E,13Z,15E,19Z-docosahexaenoic acid | 10(S),17(S)-DiHDHA | PUFA metabolite | 54845-95-3 | HMDB0003876 | C04742 | CCC=CCC(C=CC=CC=CC(CC=CCC=CCCC(=O)O)O)O | 4.07 | 4.82 | -1.31 |
| 118 | 10,17-DiHDoHE | Protectin D1 | PUFA metabolite | 145-13-1 | HMDB00253 | C01953 | CCC=CCC(C=CC=CC=CC(CC=CCC=CCCC(=O)O)O)O | 4.07 | 4.82 | -1.31 |
| 119 | (±)-18-Hydroxy-5Z,8Z,11Z,14Z,16E-eicosapentaenoic acid | 18-HEPE | PUFA metabolite | 70981-96-3 | HMDB0004244 | C05966 | CCC(C=CC=CCC=CCC=CCC=CCCCC(=O)O)O | 4.59 | 4.82 | -1.66 |
| 120 | Lithocholic acid | LCA | Bile acid | 71030-37-0 | HMDB0006111 | C14777 | OC1CCC2(C(C1)CCC1C2CCC2(C1CCC2C(CCC(=O)O)C)C)C | 5.18 | 4.79 | -1.36 |
| 121 | Allolithocholic acid | Allo-LCA | Bile acid | 71774-10-2 | HMDB0004243 | C05965 | OC1CCC2(C(C1)CCC1C2CCC2(C1CCC2C(CCC(=O)O)C)C)C | 5.18 | 4.79 | -1.36 |
| 122 | 15S-Hydroxyeicosapentaenoic acid | 15-HEPE | PUFA metabolite | 884905-07-1 | - | - | CCC=CCC(C=CC=CCC=CCC=CCCCC(=O)O)O | 4.59 | 4.82 | -1.61 |
| 123 | 5S-Hydroxy-6E,8Z,11Z,14Z,17Z-eicosapentaenoic acid | 5-HEPE | PUFA metabolite | 434-13-9 | HMDB0000761 | C03990 | CCC=CCC=CCC=CCC=CC=CC(CCCC(=O)O)O | 4.59 | 4.58 | -1.55 |
| 124 | 15S-Hydroxy-5Z,8Z,11Z,13E-eicosatetraenoic acid | 15-HETE | PUFA metabolite | 2276-94-0 | HMDB0000381 | - | CCCCCC(C=CC=CCC=CCC=CCCCC(=O)O)O | 4.84 | 4.82 | -1.58 |
| 125 | 15S-Hydroperoxy-5Z,8Z,11Z,13E-eicosatetraenoic acid | 15-HpETE | PUFA metabolite | 70608-72-9 | HMDB0011134 | C04805 | CCCCCC(C=CC=CCC=CCC=CCCCC(=O)O)OO | 5.15 | 4.82 | -4.24 |
| 126 | 5S-Hydroperoxy-6E,8Z,11Z,14Z-eicosatetraenoic acid | 5-HpETE | PUFA metabolite | 74581-83-2 | HMDB0011135 | C05356 | CCCCCC=CCC=CCC=CC=CC(CCCC(=O)O)OO | 5.15 | 4.39 | -4.24 |
| 127 | 12S-Hydroperoxy-5Z,8Z,10E,14Z-eicosatetraenoic acid | 12-HpETE | PUFA metabolite | 298-36-2 | HMDB60407 | C18040 | CCCCCC=CCC(C=CC=CCC=CCCCC(=O)O)OO | 5.15 | 4.89 | -4.24 |
| 128 | 12S-Hydroxy-5Z,8Z,10E,14Z-eicosatetraenoic acid | 12-HETE | PUFA metabolite | 303-01-5 | - | - | CCCCCC=CCC(C=CC=CCC=CCCCC(=O)O)O | 4.84 | 4.89 | -1.61 |
| 129 | (±)17-Hydroxy-4Z,7Z,10Z,13Z,15E,19Z-docosahexaenoic acid | 17-HDoHE | PUFA metabolite | 571-22-2/521-18-6 | HMDB06770/HMDB02961 | C05293/C03917 | CCC=CCC(C=CC=CCC=CCC=CCC=CCCC(=O)O)O | 5.05 | 4.89 | -1.61 |
| 130 | 25-Hydroxycholesterol-3-sulfate | 25-OHChol-S | Steroid | 1229-12-5/846-46-8 | HMDB03769/HMDB0000899 | C03772/C00674 | CC(C1CCC2C1(C)CCC1C2CC=C2C1(C)CCC(C2)OS(=O)(=O)O)CCCC(O)(C)C | 5.70 | -1.61 | -1.00 |
| 131 | 5S-Hydroxy-6E,8Z,11Z,14Z-eicosatetraenoic acid | 5-HETE | PUFA metabolite | 10417-94-4 | HMDB0001999 | C06428 | CCCCCC=CCC=CCC=CC=CC1CCCC(=O)O1 | 5.35 | 0.00 | -7.05 |
| 132 | Eicosapentaenoic acid 20:5 (n-3) | EPA | PUFA metabolite | 6217-54-5 | HMDB0002183 | C06429 | CCC=CCC=CCC=CCC=CCC=CCCCC(=O)O | 5.58 | 4.82 | 0.00 |
| 133 | 25-Hydroxycholesterol | 25-OHChol | Steroid | 2140-46-7 | HMDB0006247 | C15519 | OC1CCC2(C(=CCC3C2CCC2(C3CCC2C(CCCC(O)(C)C)C)C)C1)C | 6.01 | 18.20 | -1.02 |
| 134 | 24(S)-Hydroxycholesterol | 24-OHChol | Steroid | 128-23-4/566-65-4 | HMDB03759 | C05479/C03681 | OC1CCC2(C(=CCC3C2CCC2(C3CCC2C(CCC(C(C)C)O)C)C)C1)C | 6.03 | 18.20 | -0.95 |
| 135 | Arachidonic acid 20:4 (n-6) | AA | PUFA metabolite | 506-32-1 | HMDB0001043 | C00219 | CCCCCC=CCC=CCC=CCC=CCCCC(=O)O | 5.83 | 4.82 | 0.00 |
| 136 | 4β-Hydroxycholesterol | 4-OHChol | Steroid | 20380-11-4 | - | - | CC(CCCC(C1CCC2C1(C)CCC1C2CC=C2C1(C)CCC(C2O)O)C)C | 6.14 | 13.50 | -3.17 |
| 137 | 22β-Hydroxycholesterol | 22-OHChol | Steroid | 474-73-7 | HMDB0001419 | C13550 | CC(CCC(C(C1CCC2C1(C)CCC1C2CC=C2C1(C)CCC(C2)O)C)O)C | 6.03 | 18.20 | -0.73 |
| 138 | 20α-Hydroxycholesterol | 20-OHChol | Steroid | 72542-49-5 | - | - | CC(CCCC(C1CCC2C1(C)CCC1C2CC=C2C1(C)CCC(C2)O)(O)C)C | 6.06 | 18.20 | -0.26 |
| 139 | 24(R/S),25-Epoxycholesterol | 24,25-epoxy-Chol | Steroid | 1256-86-6 | HMDB0000653 | C18043 | OC1CCC2(C(=CCC3C2CCC2(C3CCC2C(CCC2OC2(C)C)C)C)C1)C | 5.95 | 18.20 | -1.40 |
| 140 | 27-Hydroxycholesterol | 27-OHChol | Steroid | 22348-64-7 | HMDB04035 | C05502 | OCC(CCCC(C1CCC2C1(C)CCC1C2CC=C2C1(C)CCC(C2)O)C)C | 6.03 | 17.42 | -1.23 |
| 141 | Docosahexaenoic acid 22:6 (n-3) | DHA | PUFA metabolite | 516-72-3 | HMDB06283 | C05500 | CCC=CCC=CCC=CCC=CCC=CCC=CCCC(=O)O | 6.04 | 4.89 | 0.00 |
| 142 | 7α-Hydroxycholesterol | 7-OHChol | Steroid | 566-26-7 | - | - | CC(CCCC(C1CCC2C1(C)CCC1C2C(O)C=C2C1(C)CCC(C2)O)C)C | 6.07 | 18.20 | -0.83 |
| 143 | 7-Ketocholesterol | 7-keto-Chol | Steroid | 566-28-9 | HMDB0000501 | - | CC(CCCC(C1CCC2C1(C)CCC1C2C(=O)C=C2C1(C)CCC(C2)O)C)C | 6.14 | 17.39 | -1.37 |
| 144 | Cholesterol sulfate | Chol-S | Steroid | 17320-10-4 | - | - | CC(CCCC(C1CCC2C1(C)CCC1C2CC=C2C1(C)CCC(C2)OS(=O)(=O)O)C)C | 6.70 | -1.36 | 0.00 |

**Supplemental Table S2.** Sample information used in this study.

| Sample ID | Sample name | Sex | Date of blood collection | Time of blood collection |
| --- | --- | --- | --- | --- |
| P1 | Healthy Volunteer1 | Male | 2021/4/7 | Morning (8:00-9:00) |
| P2 | Healthy Volunteer1 | Male | 2021/4/7 | Noon (12:00-13:00) |
| P3 | Healthy Volunteer2 | Male | 2021/4/7 | Morning (8:00-9:00) |
| P4 | Healthy Volunteer2 | Male | 2021/4/7 | Noon (12:00-13:00) |
| P5 | Healthy Volunteer2 | Male | 2021/4/7 | Evening (17:00-18:00) |
| P6 | Healthy Volunteer2 | Male | 2021/4/8 | Morning (8:00-9:00) |
| P7 | Healthy Volunteer3 | Male | 2021/4/14 | Morning (8:00-9:00) |
| P8 | Healthy Volunteer3 | Male | 2021/4/21 | Morning (8:00-9:00) |
| P9 | Healthy Volunteer3 | Male | 2021/4/21 | Noon (12:00-13:00) |
| P10 | Healthy Volunteer3 | Male | 2021/4/21 | Evening (17:00-18:00) |
| P11 | Healthy Volunteer3 | Male | 2021/4/28 | Morning (8:00-9:00) |
| P12 | Healthy Volunteer4 | Male | 2021/4/7 | Morning (8:00-9:00) |
| P13 | Healthy Volunteer4 | Male | 2021/4/7 | Noon (12:00-13:00) |
| P14 | Healthy Volunteer4 | Male | 2021/4/7 | Evening (17:00-18:00) |
| P15 | Healthy Volunteer4 | Male | 2021/4/8 | Morning (8:00-9:00) |
| P16 | Healthy Volunteer4 | Male | 2021/5/13 | Morning (8:00-9:00) |
| P17 | Healthy Volunteer5 | Male | 2021/4/12 | Morning (8:00-9:00) |
| P18 | Healthy Volunteer5 | Male | 2021/4/12 | Noon (12:00-13:00) |
| P19 | Healthy Volunteer5 | Male | 2021/4/12 | Evening (17:00-18:00) |
| P20 | Healthy Volunteer5 | Male | 2021/4/13 | Morning (8:00-9:00) |
| P21 | Healthy Volunteer5 | Male | 2021/5/13 | Morning (8:00-9:00) |
| P22 | Healthy Volunteer6 | Female | 2021/4/14 | Morning (8:00-9:00) |
| P23 | Healthy Volunteer7 | Female | 2021/4/19 | Morning (8:00-9:00) |
| P24 | Healthy Volunteer8 | Female | 2021/5/12 | Morning (8:00-9:00) |

**Supplemental Table S3.** Concentration of each compound in the IS mixture.

|  | Compound name | Abbreviation | Group | IS mixture conc. (µM) |
| --- | --- | --- | --- | --- |
|  |  |  |  |  |
| 145 | Corticosterone-d8 | B-d8 (IS) | Steroid (IS) | 1.40 |
| 146 | 18-Hydroxycorticosterone-9,11,12,12-d4 | 18-OHB-d4 (IS) | Steroid (IS) | 41.13 |
| 147 | 11-Deoxycorticosterone-2,2,4,6,6,17α,21,21-d8 | DOC-d8 (IS) | Steroid (IS) | 1.29 |
| 148 | 11-Deoxycortisol-2,2,4,6,6-d5 | 11-DOF-d5 (IS) | Steroid (IS) | 2.83 |
| 149 | Cortisol-9,11,12,12-d4 | F-d4 (IS) | Steroid (IS) | 1.24 |
| 150 | Cortisone-2,2,4,6,6,9,12,12-d8 | E-d8 (IS) | Steroid (IS) | 10.60 |
| 151 | Testosterone-16,16,17-d3 | T-d3 (IS) | Steroid (IS) | 0.24 |
| 152 | Cholesterol sulfate-25,26,26,26,27,27,27-d7 | Chol-S-d7 (IS) | Steroid (IS) | 7.20 |
| 153 | Cholic acid-2,2,4,4-d4 | CA-d4 (IS) | Bile acid (IS) | 0.75 |
| 154 | Chenodeoxycholic acid-2,2,4,4-d4 | CDCA-d4 (IS) | Bile acid (IS) | 4.33 |
| 155 | Deoxycholic acid-2,2,4,4-d4 | DCA-d4 (IS) | Bile acid (IS) | 0.51 |
| 156 | Lithocholic acid-2,2,4,4-d4 | LCA-d4 (IS) | Bile acid (IS) | 0.53 |
| 157 | Glycocholic acid-2,2,4,4-d4 | GCA-d4 (IS) | Bile acid (IS) | 0.43 |
| 158 | Glycodeoxycholic acid-2,2,4,4-d4 | GDCA-d4 (IS) | Bile acid (IS) | 0.24 |
| 159 | Glycolithocholic acid-2,2,4,4-d4 | GLCA-d4 (IS) | Bile acid (IS) | 0.43 |
| 160 | Taurocholic acid-2,2,4,5,5-d5 | TCA-d5 (IS) | Bile acid (IS) | 2.54 |
| 161 | Taurolithocholic acid-2,2,4,5,5-d5 | TLCA-d5 (IS) | Bile acid (IS) | 0.61 |
| 162 | Prostaglandin E2-3,3,4,4-d4 | PGE2-d4 (IS) | PUFA metabolite (IS) | 5.79 |
| 163 | Prostaglandin D2-3,3,4,4,-d4 | PGD2-d4 (IS) | PUFA metabolite (IS) | 3.83 |
| 164 | Prostaglandin F2α-3,3,4,4-d4 | PGF2α-d4 (IS) | PUFA metabolite (IS) | 13.21 |
| 165 | Thromboxane B2-3,3,4,4-d4 | TXB2-d4 (IS) | PUFA metabolite (IS) | 11.04 |
| 166 | Leukotriene D4-19,19,20,20,20-d5 | LTD4-d5 (IS) | PUFA metabolite (IS) | 15.35 |
| 167 | Leukotriene B4-6,7,14,15-d4 | LTB4-d4 (IS) | PUFA metabolite (IS) | 7.46 |
| 168 | 15(S)-Hydroxy-5Z,8Z,11Z,13E-eicosatetraenoic-5,6,8,9,11,12,14,15-d8 acid | 15-HETE-d8 (IS) | PUFA metabolite (IS) | 5.34 |
| 169 | (S,5Z,8Z,11Z,13E,17Z)-15-Hydroxyicosa-5,8,11,13,17-pentaenoic-19,19,20,20,20-d5 acid | 15-HEPE-d5 (IS) | PUFA metabolite (IS) | 1.63 |
| 170 | Docosahexaenoic acid-d5 | DHA-d5 (IS) | PUFA metabolite (IS) | 9.46 |
| 171 | Arachidonic acid-d8 | AA-d8 (IS) | PUFA metabolite (IS) | 25.44 |
| 172 | Eicosapentaenoic acid-d5 | EPA-d5 (IS) | PUFA metabolite (IS) | 11.47 |

**Supplemental Table S4.** Type and sequence of solvents used for each SPE method.

| Order of use | 1st | 2nd | 3rd | 4th | 5th | 6th | 7th |
| --- | --- | --- | --- | --- | --- | --- | --- |
| Method | Conditioning solvent | Equilibration solvent | Loading solvent | Washing solvent1 | Washing solvent2 | Washing solvent3 | Elution solvent |
| MeP-SPE1 | MeOH/FA  (100/0.1, vol/vol) | H_2_O/FA  (100/0.1, vol/vol) | MeOH/H_2_O/FA  (20/80/0.1, vol/vol/vol) | H_2_O/FA  (100/0.1, vol/vol) | EtOH/H_2_O/FA  (15/85/0.1, vol/vol/vol) | Hexane | MeOH |
| MeP-SPE2 | MeOHlFA  (100/0.1, vol/vol) | H_2_O/FA  (100/0.1, vol/vol) | MeOH/H_2_O/FA  (20/80/0.1, vol/vol/vol) | H_2_O/FA  (100/0.1, vol/vol) | - | Hexane | MeOH |
| MeP-SPE3 | MeOH/FA  (100/0.1, vol/vol) | H_2_O/FA  (100/0.1, vol/vol) | MeOH/H_2_O/FA  (20/80/0.1, vol/vol/vol) | H_2_O/FA  (100/0.1, vol/vol) | MeOH/H_2_O/FA  (10/90/0.1, vol/vol/vol) | Hexane | MeOH |
| MeP-SPE4 | MeOH/FA  (100/0.1, vol/vol) | H_2_O/FA  (100/0.1, vol/vol) | MeOH/H_2_O/FA  (20/80/0.1, vol/vol/vol) | H_2_O/FA  (100/0.1, vol/vol) | MeOH/H_2_O/FA  (10/90/0.1, vol/vol/vol) | - | MeOH |
| MeP-SPE5 | MeOH/FA  (100/0.1, vol/vol) | H_2_O/FA  (100/0.1, vol/vol) | MeOH/H_2_O/FA  (20/80/0.1, vol/vol/vol) | H_2_O/FA  (100/0.1, vol/vol) | MeOH/H_2_O/FA  (20/80/0.1, vol/vol/vol) | - | MeOH |
| MeP-SPE6 | MeOH/FA  (100/0.1, vol/vol) | H_2_O/FA  (100/0.1, vol/vol) | MeOH/H_2_O/FA  (20/80/0.1, vol/vol/vol) | H_2_O/FA  (100/0.1, vol/vol) | MeOH/H_2_O/FA  (40/60/0.1, vol/vol/vol) | - | MeOH |

MeOH: methanol, FA: formic acid, H2O: water, EtOH: ethanol.

**Supplemental Table S5.** Database IDs, MRM conditions, and analytical validation of the targeted LC/MS/MS method for steroids, bile acids, and PUFA metabolites.

|  | Compound name | Abbreviation | Molecular formula | Exact mass | Retention time (min) | | Ion species | MRM/SIM transition 1 | | | | | MRM transition 2 | | | | | LLOQ (fmol)*^a^* | ULOQ (fmol)*^b^* | RSD^c^ | Linearity (*R*^2^) |
| --- | --- | --- | --- | --- | --- | --- | --- | --- | --- | --- | --- | --- | --- | --- | --- | --- | --- | --- | --- | --- | --- |
|  |  |  |  |  | Mean | Standard deviation |  | Precursor ion | Product ion | Q1 Pre-bias (V) | Collision energy (V) | Q3 Pre-bias (V) | Precursor ion | Product ion | Q1 Pre-bias (V) | Collision energy (V) | Q3 Pre-bias (V) |  |  |  |  |
| 1 | β-Estradiol 3-(β-D-glucuronide) 17-sulfate | E2-3-G-17-S | C24H32O11S | 528.1665 | 1.49 | 0.00 | [M-H]- | 527.15 | 351.15 | 30 | 30 | 12 | 527.15 | 97.00 | 20 | 60 | 16 | 10 | 10000 | 1.5 | 0.973 |
| 2 | Prostaglandin K2 | PGK2 | C20H30O5 | 350.2093 | 1.62 | 0.00 | [M-H]- | 349.20 | 287.20 | 12 | 18 | 12 | 349.20 | 249.10 | 16 | 15 | 24 | 10 | 10000 | 1.4 | 1.000 |
| 3 | Estriol-16-glucuronide | E3-16-G | C24H32O9 | 464.2046 | 1.65 | 0.00 | [M-H]- | 463.20 | 287.00 | 10 | 33 | 19 | 463.20 | 112.90 | 13 | 27 | 23 | 40 | 10000 | 1.6 | 0.984 |
| 4 | 17β-Estradiol-3-glucuronide | E2-3-G | C24H32O8 | 448.2097 | 1.95 | 0.01 | [M-H]- | 447.20 | 271.00 | 10 | 45 | 16 | 447.20 | 112.85 | 10 | 20 | 11 | 10 | 10000 | 2.4 | 0.998 |
| 5 | (3Z)-5-[(1S,2R,3R,5S)-3,5-Dihydroxy-2-[(1E,3S)-3-hydroxy-1-octen-1-yl]cyclopentyl]-3-pentenoic acid | 2,3-dinor-8iso-PGF2α | C18H30O5 | 326.2093 | 1.97 | 0.00 | [M-H]- | 325.20 | 237.30 | 21 | 14 | 22 | 325.20 | 137.10 | 11 | 18 | 12 | 10 | 10000 | 4.4 | 0.991 |
| 6 | 6-keto-Prostaglandin F1α | 6-keto-PGF1α | C20H34O6 | 370.2355 | 2.33 | 0.01 | [M-H]- | 369.23 | 163.20 | 17 | 29 | 28 | 369.23 | 245.30 | 17 | 26 | 17 | 1000 | 100000 | 4.3 | 0.991 |
| 7 | Resolvin E1 | RvE1 | C20H30O5 | 350.2093 | 2.42 | 0.00 | [M-H]- | 349.20 | 195.20 | 24 | 16 | 19 | 349.20 | 107.10 | 12 | 21 | 10 | 40 | 10000 | 3.0 | 0.999 |
| 8 | Estrone glucuronide | E1-G | C24H30O8 | 446.1941 | 2.46 | 0.01 | [M-H]- | 445.20 | 269.15 | 13 | 38 | 12 | 445.20 | 145.15 | 23 | 63 | 29 | 10 | 10000 | 2.5 | 0.984 |
| 9 | Testosterone glucuronide | T-G | C25H36O8 | 464.2410 | 2.62 | 0.01 | [M+H]+ | 465.25 | 289.30 | -20 | -21 | -19 | 465.25 | 97.10 | -20 | -29 | -16 | 1 | 10000 | 1.6 | 0.991 |
| 10 | 18-Oxocortisol | 18-oxo-F | C21H28O6 | 376.1886 | 3.01 | 0.00 | [M+H-H2O]+ | 359.20 | 341.15 | -18 | -12 | -23 |  |  |  |  |  | 1 | 10000 | 2.7 | 0.955 |
| 11 | 18-Hydroxycortisol | 18-OHF | C21H30O6 | 378.2042 | 3.04 | 0.01 | [M+H]+ | 379.20 | 297.00 | -19 | -12 | -15 |  |  |  |  |  | 400 | 10000 | 7.4 | 0.972 |
| 12 | Thromboxane B2 | TXB2 | C20H34O6 | 370.2355 | 3.19 | 0.01 | [M-H]- | 369.25 | 169.20 | 17 | 18 | 30 | 369.25 | 195.20 | 16 | 14 | 13 | 40 | 10000 | 6.2 | 1.000 |
| 13 | 17α,20α-Dihydroxypregn-4-en-3-one | 17,20α-(OH)2Prog | C21H32O3 | 332.2351 | 3.75 | 0.00 | [M+H-H2O]+ | 315.23 | 155.15 | -24 | -18 | -30 | 315.23 | 197.10 | -26 | -12 | -20 | 1000 | 100000 | 2.7 | 0.984 |
| 14 | Prostaglandin D3 | PGD3 | C20H30O5 | 350.2093 | 3.75 | 0.00 | [M-H]- | 349.20 | 269.25 | 13 | 15 | 29 | 349.20 | 233.20 | 13 | 12 | 15 | 1000 | 100000 | 1.5 | 0.992 |
| 15 | Tauro-α-muricholic acid | TαMCA | C26H45NO7S | 515.2917 | 3.83 | 0.00 | [M-H]- | 514.30 | 514.15 | 20 | 28 | 24 |  |  |  |  |  | 1 | 10000 | 1.6 | 0.986 |
| 16 | Tauro-β-muricholic acid | TβMCA | C26H45NO7S | 515.2917 | 3.99 | 0.00 | [M-H]- | 514.30 | 514.15 | 20 | 28 | 24 |  |  |  |  |  | 1 | 10000 | 1.8 | 0.981 |
| 17 | Prostaglandin F2α | PGF2α | C20H34O5 | 354.2406 | 4.19 | 0.00 | [M-H]- | 353.25 | 309.20 | 16 | 19 | 18 | 353.25 | 193.20 | 16 | 25 | 12 | 10 | 10000 | 6.1 | 0.999 |
| 18 | ω-Muricholic acid | ω-MCA | C24H40O5 | 408.2876 | 4.24 | 0.01 | [M-H]- | 407.30 | 407.10 | 11 | 21 | 14 |  |  |  |  |  | 10 | 10000 | 2.7 | 0.996 |
| 19 | 18-Hydroxycorticosterone | 18-OHB | C21H30O5 | 362.2093 | 4.28 | 0.00 | [M+H]+ | 363.20 | 269.35 | -29 | -20 | -18 | 363.20 | 91.05 | -30 | -60 | -14 | 1 | 10000 | 1.4 | 0.963 |
| 20 | Prostaglandin E2 | PGE2 | C20H32O5 | 352.2250 | 4.33 | 0.00 | [M-H]- | 351.20 | 271.30 | 16 | 18 | 20 | 351.20 | 315.20 | 12 | 13 | 18 | 10 | 10000 | 1.5 | 0.998 |
| 21 | Prostaglandin H2 | PGH2 | C20H32O5 | 352.2250 | 4.34 | 0.00 | [M-H]- | 351.20 | 271.30 | 16 | 18 | 20 | 351.20 | 315.20 | 12 | 13 | 18 | 40 | 10000 | 2.4 | 0.996 |
| 22 | Estriol | E3 | C18H24O3 | 288.1725 | 4.37 | 0.03 | [M-H]- | 287.15 | 171.05 | 15 | 37 | 17 | 287.15 | 145.05 | 23 | 42 | 24 | 400 | 10000 | 19.1 | 0.982 |
| 23 | Prostaglandin I2 | PGI2 | C20H32O5 | 352.2250 | 4.40 | 0.00 | [M+H]+ | 353.25 | 55.10 | -30 | -46 | -25 | 353.25 | 111.20 | -30 | -21 | -22 | 1 | 10000 | 1.8 | 0.978 |
| 24 | Aldosterone | A | C21H28O5 | 360.1937 | 4.46 | 0.00 | [M+H-H2O]+ | 343.20 | 325.15 | -20 | -14 | -16 | 343.20 | 315.20 | -19 | -14 | -22 | 10 | 10000 | 2.7 | 0.946 |
| 25 | α-Muricholic acid | α-MCA | C24H40O5 | 408.2876 | 4.55 | 0.01 | [M-H]- | 407.30 | 407.10 | 11 | 21 | 14 |  |  |  |  |  | 10 | 10000 | 6.1 | 0.999 |
| 26 | 11β,17α,21-Trihydroxypregnenolone | 11β,17α,21-(OH)3Preg | C21H32O5 | 364.2250 | 4.62 | 0.00 | [M-H]- | 363.20 | 333.25 | 11 | 15 | 11 | 363.20 | 299.00 | 11 | 26 | 14 | 100 | 10000 | 13.4 | 0.967 |
| 27 | 7α-Hydroxytestosterone | 7α-OHT | C19H28O3 | 304.2038 | 4.63 | 0.00 | [M+H]+ | 305.20 | 269.30 | -26 | -19 | -17 | 305.20 | 287.20 | -26 | -17 | -29 | 1 | 10000 | 2.5 | 0.993 |
| 28 | Prostaglandin E1 | PGE1 | C20H34O5 | 354.2406 | 4.78 | 0.00 | [M-H]- | 353.25 | 317.20 | 16 | 15 | 20 | 353.25 | 335.10 | 16 | 11 | 11 | 10 | 10000 | 2.4 | 0.999 |
| 29 | Prostaglandin D2 | PGD2 | C20H32O5 | 352.2250 | 4.83 | 0.00 | [M-H]- | 351.20 | 271.30 | 16 | 18 | 20 | 351.20 | 315.20 | 12 | 13 | 18 | 10 | 10000 | 1.3 | 0.999 |
| 30 | 19-Hydroxytestosterone | 19-OHT | C19H28O3 | 304.2038 | 4.84 | 0.00 | [M+H]+ | 305.20 | 269.20 | -26 | -17 | -17 | 305.20 | 91.15 | -14 | -53 | -15 | 1 | 10000 | 1.0 | 0.959 |
| 31 | β-Muricholic acid | β-MCA | C24H40O5 | 408.2876 | 4.95 | 0.00 | [M-H]- | 407.30 | 407.10 | 11 | 21 | 14 |  |  |  |  |  | 10 | 10000 | 2.1 | 0.999 |
| 32 | Etiocholanolone glucuronide | Et-G | C25H38O8 | 466.2567 | 5.14 | 0.00 | [M-H]- | 465.25 | 112.90 | 27 | 28 | 29 | 465.25 | 75.10 | 11 | 32 | 30 | 10 | 10000 | 4.8 | 0.995 |
| 33 | Androsterone glucuronide | An-G | C25H38O8 | 466.2567 | 5.19 | 0.01 | [M-H]- | 465.25 | 112.85 | 25 | 30 | 22 | 465.25 | 75.00 | 14 | 33 | 29 | 40 | 10000 | 2.2 | 0.996 |
| 34 | Estrone sulfate | E1-S | C18H22O5S | 350.1188 | 5.22 | 0.00 | [M-H]- | 349.10 | 269.10 | 24 | 34 | 27 | 349.10 | 142.90 | 27 | 64 | 30 | 1 | 10000 | 2.0 | 0.986 |
| 35 | Resolvin D2 | RvD2 | C22H32O5 | 376.2250 | 5.27 | 0.00 | [M-H]- | 375.20 | 175.20 | 25 | 23 | 17 | 375.20 | 141.10 | 17 | 16 | 24 | 10 | 10000 | 4.4 | 0.999 |
| 36 | 5(S),14(R)-Lipoxin B4 | LXB4 | C20H32O5 | 352.2250 | 5.37 | 0.01 | [M-H]- | 351.20 | 221.20 | 23 | 16 | 23 | 351.20 | 315.10 | 15 | 15 | 14 | 100 | 10000 | 2.3 | 0.996 |
| 37 | Resolvin D3 | RvD3 | C22H32O5 | 376.2250 | 5.52 | 0.00 | [M-H]- | 375.20 | 147.30 | 13 | 22 | 14 | 375.20 | 115.20 | 17 | 22 | 11 | 10 | 10000 | 1.9 | 0.999 |
| 38 | Prostaglandin G2 | PGG2 | C20H32O6 | 368.2199 | 5.58 | 0.00 | [M-H]- | 367.20 | 235.20 | 17 | 10 | 23 |  |  |  |  |  | 40 | 10000 | 1.7 | 0.850 |
| 39 | 19-Hydroxyandrostenedione | 19-OHA4 | C19H26O3 | 302.1882 | 5.58 | 0.00 | [M+H]+ | 303.20 | 267.20 | -26 | -18 | -12 | 303.20 | 255.30 | -25 | -18 | -11 | 1 | 10000 | 2.2 | 0.971 |
| 40 | Cortisone | E | C21H28O5 | 360.1937 | 5.59 | 0.00 | [M+H]+ | 361.20 | 163.20 | -30 | -25 | -30 | 361.20 | 121.20 | -30 | -30 | -25 | 1 | 10000 | 3.2 | 0.983 |
| 41 | Cortisol | F | C21H30O5 | 362.2093 | 5.62 | 0.00 | [M+H]+ | 363.20 | 121.15 | -30 | -25 | -26 | 363.20 | 327.25 | -16 | -18 | -11 | 1 | 10000 | 0.6 | 0.967 |
| 42 | Glycoursodeoxycholic acid | GUDCA | C26H43NO5 | 449.3141 | 5.76 | 0.00 | [M-H]- | 448.30 | 448.15 | 13 | 19 | 16 | 448.30 | 74.10 | 10 | 45 | 14 | 10 | 10000 | 3.8 | 1.000 |
| 43 | 7α-Hydroxydehydroepiandrosterone | 7α-OHDHEA | C19H28O3 | 304.2038 | 5.90 | 0.01 | [M+H-H2O]+ | 287.20 | 269.15 | -11 | -13 | -28 | 287.20 | 211.00 | -14 | -19 | -21 | 40 | 10000 | 5.0 | 0.990 |
| 44 | Glycohyodeoxycholic acid | GHDCA | C26H43NO5 | 449.3141 | 5.92 | 0.00 | [M-H]- | 448.30 | 448.15 | 13 | 19 | 16 | 448.30 | 74.10 | 10 | 45 | 14 | 1 | 10000 | 1.1 | 1.000 |
| 45 | 7α-Hydroxyandrostenedione | 7α-OHA4 | C19H26O3 | 302.1882 | 5.96 | 0.00 | [M+H]+ | 303.20 | 285.35 | -26 | -19 | -19 | 303.20 | 97.15 | -25 | -26 | -24 | 10 | 10000 | 6.0 | 0.979 |
| 46 | Dehydroepiandrosterone sulfate | DHEA-S | C19H28O5S | 368.1657 | 6.22 | 0.00 | [M-H]- | 367.15 | 96.90 | 11 | 46 | 19 | 367.15 | 80.00 | 11 | 65 | 15 | 10 | 10000 | 0.7 | 0.971 |
| 47 | 16α-Hydroxyestrone | 16α-OHE1 | C18H22O3 | 286.1569 | 6.29 | 0.01 | [M-H]- | 285.15 | 144.90 | 15 | 38 | 14 | 285.15 | 143.00 | 12 | 56 | 14 | 100 | 10000 | 9.5 | 1.000 |
| 48 | Glycocholic acid | GCA | C26H43NO6 | 465.3090 | 6.42 | 0.00 | [M-H]- | 464.30 | 464.15 | 11 | 17 | 16 | 464.30 | 73.95 | 17 | 45 | 14 | 1 | 10000 | 1.4 | 0.991 |
| 49 | Tauroursodeoxycholic acid | TUDCA | C26H45NO6S | 499.2968 | 6.46 | 0.00 | [M-H]- | 498.30 | 498.20 | 12 | 28 | 17 |  |  |  |  |  | 1 | 10000 | 0.4 | 0.988 |
| 50 | Taurohyodeoxycholic acid | THDCA | C26H45NO6S | 499.2968 | 6.65 | 0.00 | [M-H]- | 498.30 | 498.20 | 12 | 28 | 17 |  |  |  |  |  | 10 | 10000 | 1.1 | 0.972 |
| 51 | Resolvin D1 | RvD1 | C22H32O5 | 376.2250 | 6.76 | 0.00 | [M-H]- | 375.20 | 141.10 | 17 | 16 | 24 | 375.20 | 121.10 | 13 | 31 | 10 | 10 | 10000 | 19.9 | 0.939 |
| 52 | 16α-Hydroxyandrostenedione | 16α-OHA4 | C19H26O3 | 302.1882 | 6.86 | 0.00 | [M+H]+ | 303.20 | 97.20 | -25 | -24 | -20 | 303.20 | 109.20 | -25 | -26 | -28 | 1 | 10000 | 3.5 | 1.000 |
| 53 | Taurocholic acid | TCA | C26H45NO7S | 515.2917 | 6.98 | 0.00 | [M-H]- | 514.30 | 514.15 | 20 | 28 | 24 | 514.30 | 124.05 | 22 | 57 | 10 | 1 | 10000 | 2.1 | 0.981 |
| 54 | 11-Dehydrocorticosterone | 11-DHB | C21H28O4 | 344.1988 | 6.98 | 0.00 | [M+H]+ | 345.20 | 121.25 | -29 | -26 | -30 | 345.20 | 301.20 | -29 | -24 | -19 | 1 | 10000 | 4.0 | 0.992 |
| 55 | 11β-Hydroxytestosterone | 11β-OHT | C19H28O3 | 304.2038 | 7.06 | 0.00 | [M+H]+ | 305.20 | 269.25 | -26 | -18 | -26 | 305.20 | 121.20 | -26 | -24 | -11 | 1 | 10000 | 1.9 | 0.962 |
| 56 | 16α-Hydroxydehydroisoandrosterone | 16α-OHDHEA | C19H28O3 | 304.2038 | 7.15 | 0.02 | [M+H]+ | 305.21 | 269.20 | -16 | -13 | -17 | 305.21 | 287.20 | -25 | -11 | -19 | 4000 | 100000 | 1.6 | 0.997 |
| 57 | 5(S),6(R)-Lipoxin A4/15-epi-Lipoxin A4 | LXA4/15-epi-LXA4 | C20H32O5 | 352.2250 | 7.17 | 0.00 | [M-H]- | 351.20 | 115.20 | 16 | 15 | 19 | 351.20 | 217.20 | 12 | 22 | 12 | 10 | 10000 | 2.0 | 0.998 |
| 58 | Prostaglandin A2 | PGA2 | C20H30O4 | 334.2144 | 7.36 | 0.00 | [M-H]- | 333.20 | 271.20 | 15 | 16 | 27 | 333.20 | 189.20 | 27 | 20 | 16 | 10 | 10000 | 2.3 | 0.993 |
| 59 | Adrenosterone | 11-keto-A4 | C19H24O3 | 300.1725 | 7.58 | 0.00 | [M+H]+ | 301.20 | 257.20 | -25 | -25 | -17 | 301.20 | 121.20 | -24 | -26 | -25 | 1 | 10000 | 4.7 | 0.998 |
| 60 | 5β-Dihydrocortisol | 5β-DiHF | C21H32O5 | 364.2250 | 7.73 | 0.00 | [M+CH3COO]- | 423.25 | 333.30 | 11 | 22 | 11 | 423.25 | 299.10 | 11 | 38 | 14 | 10 | 10000 | 0.9 | 0.960 |
| 61 | Prostaglandin B2 | PGB2 | C20H30O4 | 334.2144 | 7.76 | 0.01 | [M-H]- | 333.20 | 175.20 | 22 | 20 | 15 | 333.20 | 235.20 | 15 | 20 | 10 | 10 | 10000 | 2.5 | 0.999 |
| 62 | Leukotriene C4 | LTC4 | C30H47N3O9S | 625.3033 | 7.81 | 0.01 | [M-H]- | 624.30 | 272.20 | 22 | 25 | 14 | 624.30 | 254.10 | 22 | 26 | 11 | 1000 | 100000 | 4.1 | 0.987 |
| 63 | 11β-Hydroxyandrostenedione | 11β-OHA4 | C19H26O3 | 302.1882 | 7.88 | 0.00 | [M+H]+ | 303.20 | 285.25 | -25 | -17 | -18 | 303.20 | 267.25 | -25 | -19 | -18 | 1 | 10000 | 2.0 | 0.977 |
| 64 | Cholic acid | CA | C24H40O5 | 408.2876 | 7.90 | 0.00 | [M-H]- | 407.30 | 407.10 | 11 | 21 | 14 | 407.30 | 343.05 | 17 | 34 | 14 | 1 | 10000 | 1.0 | 0.995 |
| 65 | 17α,21-Dihydroxy-5β-pregnane-3,11,20-trione | 5β-DiHE | C21H30O5 | 362.2093 | 7.90 | 0.00 | [M+CH3COO]- | 421.20 | 331.10 | 11 | 30 | 11 | 421.20 | 303.15 | 11 | 23 | 10 | 10 | 10000 | 4.7 | 0.979 |
| 66 | 21-Deoxycortisol | 21-DOF | C21H30O4 | 346.2144 | 7.93 | 0.00 | [M+H]+ | 347.20 | 311.20 | -29 | -18 | -20 | 347.20 | 91.20 | -16 | -58 | -15 | 1 | 10000 | 1.2 | 0.992 |
| 67 | 7-ketodeoxycholic acid | 7-keto-DCA | C24H38O5 | 406.2719 | 7.94 | 0.00 | [M-H]- | 405.25 | 405.20 | 20 | 17 | 20 | 405.25 | 289.30 | 14 | 37 | 18 | 40 | 10000 | 5.5 | 0.997 |
| 68 | Corticosterone | B | C21H30O4 | 346.2144 | 7.94 | 0.00 | [M+H]+ | 347.20 | 329.30 | -29 | -16 | -23 | 347.20 | 121.10 | -15 | -26 | -11 | 1 | 10000 | 1.7 | 0.970 |
| 69 | 17α,21-Dihydroxypregnenolone | 17,21-(OH)2Preg | C21H32O4 | 348.2301 | 7.96 | 0.02 | [M+CH3COO]- | 407.25 | 317.20 | 12 | 21 | 14 |  |  |  |  |  | 100 | 10000 | 23.1 | 0.862 |
| 70 | Ursodeoxycholic acid | UDCA | C24H40O4 | 392.2927 | 8.07 | 0.00 | [M-H]- | 391.30 | 391.15 | 11 | 20 | 13 |  |  |  |  |  | 1 | 10000 | 1.8 | 0.999 |
| 71 | 2-Hydroxyestradiol | 2-OHE2 | C18H24O3 | 288.1725 | 8.31 | 0.01 | [M-H]- | 287.15 | 255.20 | 25 | 42 | 25 | 287.15 | 161.95 | 15 | 33 | 16 | 100 | 10000 | 8.5 | 0.835 |
| 72 | 11-Deoxycortisol | 11-DOF | C21H30O4 | 346.2144 | 8.42 | 0.00 | [M+H]+ | 347.20 | 97.20 | -28 | -28 | -19 | 347.20 | 109.10 | -30 | -29 | -10 | 1 | 10000 | 1.4 | 0.964 |
| 73 | Hyodeoxycholic acid | HDCA | C24H40O4 | 392.2927 | 8.58 | 0.01 | [M-H]- | 391.30 | 391.15 | 11 | 20 | 13 |  |  |  |  |  | 1 | 10000 | 3.4 | 0.999 |
| 74 | Resolvin D4 | RvD4 | C22H32O5 | 376.2250 | 8.85 | 0.00 | [M-H]- | 375.20 | 101.00 | 25 | 21 | 15 | 375.20 | 131.10 | 25 | 21 | 15 | 10 | 10000 | 2.8 | 0.976 |
| 75 | 7α-Hydroxypregnenolone | 7α-OHPreg | C21H32O3 | 332.2351 | 8.89 | 0.01 | [M+H-H2O]+ | 315.25 | 297.10 | -16 | -12 | -30 | 315.25 | 279.00 | -16 | -13 | -18 | 10 | 10000 | 2.2 | 0.988 |
| 76 | 2-Hydroxyestrone | 2-OHE1 | C18H22O3 | 286.1569 | 8.97 | 0.00 | [M-H]- | 285.15 | 160.95 | 14 | 40 | 15 | 285.15 | 158.95 | 14 | 52 | 15 | 10 | 10000 | 1.5 | 0.992 |
| 77 | 7-Ketolithocholic acid | 7-keto-LCA | C24H38O4 | 390.2770 | 9.06 | 0.00 | [M-H]- | 389.25 | 389.10 | 12 | 20 | 13 |  |  |  |  |  | 10 | 10000 | 3.5 | 0.999 |
| 78 | Tetrahydrocorticosterone | THB | C21H34O4 | 350.2457 | 9.22 | 0.01 | [M+CH3COO]- | 409.25 | 59.00 | 13 | 21 | 23 |  |  |  |  |  | 100 | 10000 | 6.8 | 1.000 |
| 79 | 3α,21-Dihydroxy-5β-pregnane-11,20-dione | THA | C21H32O4 | 348.2301 | 9.34 | 0.00 | [M+CH3COO]- | 407.25 | 59.00 | 12 | 20 | 22 | 407.25 | 347.25 | 11 | 14 | 16 | 10 | 10000 | 1.3 | 0.993 |
| 80 | Pregnenolone sulfate | Preg-S | C21H32O5S | 396.1970 | 9.35 | 0.00 | [M-H]- | 395.20 | 96.90 | 18 | 50 | 21 | 395.20 | 80.10 | 11 | 65 | 16 | 1 | 10000 | 0.6 | 0.980 |
| 81 | Leukotriene D4 | LTD4 | C25H40N2O6S | 496.2607 | 9.44 | 0.00 | [M-H]- | 495.25 | 177.10 | 23 | 21 | 30 | 495.25 | 143.15 | 13 | 26 | 12 | 10 | 10000 | 1.3 | 1.000 |
| 82 | 12-Ketolithocholic acid | 12-keto-LCA | C24H38O4 | 390.2770 | 9.44 | 0.00 | [M-H]- | 389.25 | 389.10 | 12 | 20 | 13 |  |  |  |  |  | 10 | 10000 | 1.2 | 0.994 |
| 83 | Glycochenodeoxycholic acid | GCDCA | C26H43NO5 | 449.3141 | 9.55 | 0.00 | [M-H]- | 448.30 | 448.15 | 13 | 19 | 16 |  |  |  |  |  | 1 | 10000 | 1.6 | 0.998 |
| 84 | 10(S),17(S)-dihydroxy-4Z,7Z,11E,13Z,15E,19Z-docosahexaenoic acid | 10(S),17(S)-DiHDHA | C22H32O4 | 360.2301 | 9.77 | 0.01 | [M-H]- | 359.20 | 153.10 | 13 | 17 | 29 | 359.20 | 93.00 | 13 | 28 | 16 | 10 | 10000 | 1.2 | 0.999 |
| 85 | 10,17-DiHDoHE | Protectin D1 | C22H32O4 | 360.2301 | 9.77 | 0.00 | [M-H]- | 359.20 | 153.20 | 16 | 18 | 26 | 359.20 | 92.90 | 23 | 28 | 13 | 10 | 10000 | 4.9 | 1.000 |
| 86 | 11β,21-Dihydroxy-5β-pregnane-3,20-dione | 5β-DiHB | C21H32O4 | 348.2301 | 9.86 | 0.00 | [M+CH3COO]- | 407.25 | 59.00 | 11 | 21 | 22 | 407.25 | 347.30 | 16 | 15 | 17 | 100 | 10000 | 3.1 | 0.956 |
| 87 | Taurochenodeoxycholic acid | TCDCA | C26H45NO6S | 499.2968 | 10.04 | 0.01 | [M-H]- | 498.30 | 498.20 | 12 | 28 | 17 |  |  |  |  |  | 1 | 10000 | 1.5 | 0.995 |
| 88 | Maresin-1 | Maresin-1 | C22H32O4 | 360.2301 | 10.14 | 0.01 | [M-H]- | 359.20 | 92.90 | 23 | 28 | 13 | 359.20 | 177.30 | 12 | 16 | 10 | 40 | 10000 | 3.6 | 0.986 |
| 89 | Glycodeoxycholic acid | GDCA | C26H43NO5 | 449.3141 | 10.41 | 0.00 | [M-H]- | 448.30 | 448.15 | 13 | 19 | 16 |  |  |  |  |  | 10 | 10000 | 0.4 | 0.999 |
| 90 | Estradiol | E2 | C18H24O2 | 272.1776 | 10.70 | 0.01 | [M+H-H2O]+ | 255.15 | 159.10 | -14 | -20 | -16 | 255.15 | 199.20 | -14 | -20 | -22 | 1000 | 100000 | 1.8 | 0.999 |
| 91 | 11-Deoxycorticosterone | DOC | C21H30O3 | 330.2195 | 10.81 | 0.00 | [M+H]+ | 331.25 | 97.20 | -28 | -23 | -20 | 331.25 | 109.20 | -28 | -27 | -23 | 1 | 10000 | 1.0 | 0.961 |
| 92 | Taurodeoxychloic acid | TDCA | C26H45NO6S | 499.2968 | 10.81 | 0.01 | [M-H]- | 498.30 | 498.20 | 12 | 28 | 17 |  |  |  |  |  | 1 | 10000 | 2.4 | 0.991 |
| 93 | Testosterone | T | C19H28O2 | 288.2089 | 10.89 | 0.01 | [M+H]+ | 289.20 | 97.20 | -24 | -25 | -21 | 289.20 | 109.20 | -13 | -24 | -23 | 1 | 10000 | 1.3 | 0.986 |
| 94 | Leukotriene E4 | LTE4 | C23H37NO5S | 439.2392 | 10.94 | 0.01 | [M-H]- | 438.25 | 333.10 | 20 | 20 | 21 | 438.25 | 420.10 | 29 | 14 | 14 | 40 | 10000 | 2.6 | 0.996 |
| 95 | Leukotriene B4 | LTB4 | C20H32O4 | 336.2301 | 11.06 | 0.01 | [M-H]- | 335.20 | 195.20 | 15 | 16 | 18 | 335.20 | 59.00 | 15 | 22 | 12 | 40 | 10000 | 3.3 | 0.995 |
| 96 | Androstenedione | A4 | C19H26O2 | 286.1933 | 11.11 | 0.00 | [M+H]+ | 287.20 | 97.10 | -24 | -24 | -20 | 287.20 | 109.10 | -24 | -26 | -18 | 1 | 10000 | 1.1 | 0.990 |
| 97 | 11β-Hydroxyprogesterone | 11β-OHProg | C21H30O3 | 330.2195 | 11.17 | 0.00 | [M+H]+ | 331.25 | 313.25 | -28 | -17 | -29 | 331.25 | 295.30 | -28 | -17 | -10 | 1 | 10000 | 1.1 | 0.972 |
| 98 | 21-Hydroxypregnenolone | 21-OHPreg | C21H32O3 | 332.2351 | 11.22 | 0.01 | [M+H-H2O]+ | 315.25 | 297.25 | -30 | -13 | -20 | 315.25 | 279.10 | -12 | -15 | -14 | 1000 | 100000 | 5.6 | 0.998 |
| 99 | Estrone | E1 | C18H22O2 | 270.1620 | 11.29 | 0.01 | [M+H-H2O]+ | 253.15 | 157.10 | -15 | -21 | -29 | 253.15 | 197.15 | -16 | -21 | -14 | 1000 | 100000 | 7.2 | 0.986 |
| 100 | 2-Methoxyestrone | 2-ME1 | C19H24O3 | 300.1725 | 11.83 | 0.00 | [M-H]- | 299.15 | 284.00 | 11 | 24 | 12 |  |  |  |  |  | 100 | 10000 | 7.4 | 0.996 |
| 101 | 17α-Hydroxypregnenolone | 17α-OHPreg | C21H32O3 | 332.2351 | 11.85 | 0.00 | [M+H-H2O]+ | 315.25 | 297.30 | -14 | -14 | -10 |  |  |  |  |  | 100 | 10000 | 9.2 | 0.952 |
| 102 | 17α-Hydroxyprogesterone | 17α-OHProg | C21H30O3 | 330.2195 | 11.89 | 0.00 | [M+H]+ | 331.25 | 97.15 | -28 | -27 | -21 | 331.25 | 109.20 | -28 | -27 | -10 | 1 | 10000 | 1.4 | 0.997 |
| 103 | Methyltestosterone | 17α-MeT | C20H30O2 | 302.2246 | 12.01 | 0.01 | [M+H]+ | 303.25 | 97.20 | -26 | -27 | -21 | 303.25 | 109.20 | -26 | -28 | -10 | 1 | 10000 | 1.4 | 0.987 |
| 104 | Dehydroepiandrosterone | DHEA | C19H28O2 | 288.2089 | 12.07 | 0.01 | [M+H]+ | 289.20 | 271.05 | -15 | -14 | -19 |  |  |  |  |  | 400 | 10000 | 13.3 | 0.914 |
| 105 | Chenodeoxycholic acid/Allochenodeoxycholic acid | CDCA/Allo-CDCA | C24H40O4 | 392.2927 | 12.24 | 0.01 | [M-H]- | 391.30 | 391.15 | 11 | 20 | 13 |  |  |  |  |  | 10 | 10000 | 5.7 | 1.000 |
| 106 | Deoxycholic acid | DCA | C24H40O4 | 392.2927 | 12.81 | 0.00 | [M-H]- | 391.30 | 391.15 | 11 | 20 | 13 |  |  |  |  |  | 1 | 10000 | 2.1 | 0.999 |
| 107 | (±)-18-Hydroxy-5Z,8Z,11Z,14Z,16E-eicosapentaenoic acid | 18-HEPE | C20H30O3 | 318.2195 | 13.17 | 0.00 | [M-H]- | 317.20 | 255.25 | 21 | 12 | 26 | 317.20 | 259.20 | 11 | 12 | 17 | 10 | 10000 | 1.4 | 0.994 |
| 108 | Glycolithocholic acid | GLCA | C26H43NO4 | 433.3192 | 13.21 | 0.01 | [M-H]- | 432.30 | 432.20 | 10 | 15 | 15 | 432.30 | 74.10 | 14 | 50 | 13 | 10 | 10000 | 2.0 | 0.994 |
| 109 | Taurolithocholic acid | TLCA | C26H45NO5S | 483.3018 | 13.55 | 0.01 | [M-H]- | 482.30 | 482.15 | 14 | 26 | 10 | 482.30 | 80.00 | 17 | 65 | 11 | 1 | 10000 | 1.8 | 0.993 |
| 110 | 15S-Hydroxyeicosapentaenoic acid | 15-HEPE | C20H30O3 | 318.2195 | 13.87 | 0.01 | [M-H]- | 317.20 | 255.25 | 21 | 13 | 27 | 317.20 | 219.15 | 11 | 13 | 14 | 40 | 10000 | 2.6 | 0.999 |
| 111 | 20α-Hydroxyprogesterone | 20α-OHProg | C21H32O2 | 316.2402 | 14.03 | 0.00 | [M+H]+ | 317.25 | 97.20 | -27 | -25 | -21 | 317.25 | 109.10 | -27 | -26 | -10 | 1 | 10000 | 0.2 | 0.929 |
| 112 | Etiocholanolone | Et | C19H30O2 | 290.2246 | 14.70 | 0.01 | [M+H]+ | 291.25 | 255.10 | -25 | -15 | -26 | 291.25 | 273.30 | -26 | -10 | -17 | 1000 | 10000 | 7.1 | 0.995 |
| 113 | Progesterone | Prog | C21H30O2 | 314.2246 | 15.07 | 0.00 | [M+H]+ | 315.25 | 97.20 | -27 | -23 | -16 | 315.25 | 109.20 | -27 | -25 | -10 | 1 | 10000 | 0.7 | 0.957 |
| 114 | Androsterone | An | C19H30O2 | 290.2246 | 15.10 | 0.01 | [M+H]+ | 291.25 | 273.25 | -25 | -11 | -28 | 291.25 | 255.25 | -25 | -16 | -17 | 100 | 10000 | 3.9 | 0.980 |
| 115 | 5S-Hydroxy-6E,8Z,11Z,14Z,17Z-eicosapentaenoic acid | 5-HEPE | C20H30O3 | 318.2195 | 15.48 | 0.01 | [M-H]- | 317.20 | 255.25 | 21 | 12 | 28 | 317.20 | 115.00 | 11 | 14 | 11 | 10 | 10000 | 1.6 | 0.997 |
| 116 | (±)17-Hydroxy-4Z,7Z,10Z,13Z,15E,19Z-docosahexaenoic acid | 17-HDoHE | C22H32O3 | 344.2351 | 15.58 | 0.01 | [M-H]- | 343.25 | 281.40 | 16 | 13 | 29 |  |  |  |  |  | 40 | 10000 | 3.6 | 0.987 |
| 117 | 15S-Hydroxy-5Z,8Z,11Z,13E-eicosatetraenoic acid | 15-HETE | C20H32O3 | 320.2351 | 15.62 | 0.00 | [M-H]- | 319.25 | 219.20 | 15 | 13 | 12 | 319.25 | 301.20 | 15 | 11 | 18 | 10 | 10000 | 2.5 | 0.999 |
| 118 | Pregnenolone | Preg | C21H32O2 | 316.2402 | 16.05 | 0.02 | [M+H]+ | 317.25 | 93.15 | -30 | -29 | -22 | 317.25 | 299.10 | -27 | -14 | -13 | 400 | 10000 | 11.9 | 0.994 |
| 119 | 15S-Hydroperoxy-5Z,8Z,11Z,13E-eicosatetraenoic acid | 15-HpETE | C20H32O4 | 336.2301 | 16.05 | 0.00 | [M-H]- | 335.20 | 113.20 | 16 | 11 | 19 | 335.20 | 139.10 | 21 | 15 | 19 | 40 | 10000 | 1.7 | 0.994 |
| 120 | 12S-Hydroxy-5Z,8Z,10E,14Z-eicosatetraenoic acid | 12-HETE | C20H32O3 | 320.2351 | 16.38 | 0.01 | [M-H]- | 319.25 | 179.20 | 22 | 15 | 30 | 319.25 | 301.20 | 15 | 11 | 18 | 10 | 10000 | 3.7 | 1.000 |
| 121 | 12S-Hydroperoxy-5Z,8Z,10E,14Z-eicosatetraenoic acid | 12-HpETE | C20H32O4 | 336.2301 | 16.70 | 0.01 | [M-H]- | 335.20 | 153.20 | 21 | 10 | 12 | 335.20 | 179.30 | 21 | 10 | 12 | 100 | 10000 | 1.8 | 0.996 |
| 122 | 25-Hydroxycholesterol-3-sulfate | 25-OHChol-S | C27H46O5S | 482.3066 | 16.73 | 0.00 | [M-H]- | 481.30 | 96.90 | 15 | 39 | 19 |  |  |  |  |  | 1 | 10000 | 0.8 | 0.989 |
| 123 | Lithocholic acid | LCA | C24H40O3 | 376.2977 | 16.91 | 0.00 | [M-H]- | 375.30 | 375.15 | 14 | 20 | 13 |  |  |  |  |  | 1 | 10000 | 0.8 | 0.999 |
| 124 | Allolithocholic acid | Allo-LCA | C24H40O3 | 376.2977 | 17.12 | 0.00 | [M-H]- | 375.30 | 375.15 | 14 | 20 | 13 |  |  |  |  |  | 10 | 10000 | 1.5 | 1.000 |
| 125 | 5S-Hydroxy-6E,8Z,11Z,14Z-eicosatetraenoic acid | 5-HETE | C20H32O3 | 320.2351 | 17.52 | 0.01 | [M-H]- | 319.25 | 115.10 | 11 | 15 | 10 | 319.25 | 301.20 | 15 | 11 | 18 | 40 | 10000 | 1.6 | 0.998 |
| 126 | 5S-Hydroperoxy-6E,8Z,11Z,14Z-eicosatetraenoic acid | 5-HpETE | C20H32O4 | 336.2301 | 17.63 | 0.01 | [M-H]- | 335.20 | 155.10 | 15 | 15 | 14 | 335.20 | 129.10 | 15 | 13 | 10 | 40 | 10000 | 6.0 | 0.999 |
| 127 | 5α-Dihydrodeoxycorticosterone | 5α-DiHDOC | C21H32O3 | 332.2351 | 18.70 | 0.02 | [M+H]+ | 333.25 | 315.10 | -29 | -16 | -14 | 333.25 | 297.05 | -14 | -18 | -13 | 40 | 10000 | 1.1 | 0.908 |
| 128 | 5β-Dihydrodeoxycorticosterone | 5β-DiHDOC | C21H32O3 | 332.2351 | 18.90 | 0.01 | [M+H]+ | 333.25 | 315.20 | -12 | -13 | -16 | 333.25 | 292.00 | -16 | -7 | -14 | 10 | 4000 | 2.0 | 0.999 |
| 129 | 5β-Dihydrotestosterone/Dihydrotestosterone | 5β-DHT/DHT | C19H30O2 | 290.2246 | 19.28 | 0.01 | [M+H-H2O]+ | 273.20 | 255.20 | -15 | -14 | -28 | 273.20 | 199.15 | -14 | -15 | -13 | 40 | 4000 | 2.2 | 0.994 |
| 130 | Etiocholanedione/5α-Androstanedione | ED/AD | C19H28O2 | 288.2089 | 19.47 | 0.01 | [M+H]+ | 289.20 | 271.30 | -24 | -14 | -18 | 289.20 | 213.20 | -23 | -22 | -15 | 10 | 10000 | 1.0 | 0.906 |
| 131 | Eicosapentaenoic acid 20:5 (n-3) | EPA | C20H30O2 | 302.2246 | 20.57 | 0.01 | [M-H]- | 301.20 | 257.25 | 21 | 12 | 28 | 301.20 | 203.05 | 11 | 13 | 19 | 40 | 10000 | 2.1 | 1.000 |
| 132 | Docosahexaenoic acid 22:6 (n-3) | DHA | C22H32O2 | 328.2402 | 21.17 | 0.00 | [M-H]- | 327.25 | 283.30 | 23 | 11 | 30 | 327.25 | 229.25 | 12 | 13 | 23 | 10 | 10000 | 2.7 | 0.991 |
| 133 | 25-Hydroxycholesterol | 25-OHChol | C27H46O2 | 402.3498 | 21.36 | 0.02 | [M+H-H2O]+ | 385.35 | 367.20 | -13 | -14 | -17 | 385.35 | 133.10 | -19 | -35 | -26 | 400 | 10000 | 1.6 | 0.996 |
| 134 | 5β-Dihydroprogesterone/5α-Dihydroprogesterone | 5β-DiHProg/5α-DiHProg | C21H32O2 | 316.2402 | 21.38 | 0.00 | [M+H]+ | 317.25 | 281.25 | -18 | -16 | -19 | 317.25 | 299.20 | -10 | -11 | -11 | 10 | 10000 | 1.3 | 0.917 |
| 135 | Arachidonic acid 20:4 (n-6) | AA | C20H32O2 | 304.2402 | 21.43 | 0.01 | [M-H]- | 303.25 | 259.20 | 21 | 13 | 27 | 303.25 | 59.00 | 21 | 22 | 10 | 10 | 10000 | 4.4 | 0.990 |
| 136 | 27-Hydroxycholesterol | 27-OHChol | C27H46O2 | 402.3498 | 21.52 | 0.01 | [M+H-H2O]+ | 385.35 | 81.00 | -27 | -40 | -30 | 385.35 | 367.20 | -13 | -14 | -17 | 10 | 10000 | 10.6 | 0.926 |
| 137 | 24(S)-Hydroxycholesterol | 24-OHChol | C27H46O2 | 402.3498 | 21.66 | 0.01 | [M+H-H2O]+ | 385.35 | 367.20 | -13 | -14 | -17 | 385.35 | 81.00 | -27 | -40 | -30 | 400 | 10000 | 3.3 | 0.976 |
| 138 | 24(R/S),25-Epoxycholesterol | 24,25-epoxy-Chol | C27H44O2 | 400.3341 | 22.06 | 0.01 | [M+H-H2O]+ | 383.35 | 161.00 | -19 | -22 | -16 | 383.35 | 365.20 | -13 | -15 | -12 | 10 | 4000 | 6.1 | 0.997 |
| 139 | Cholesterol sulfate | Chol-S | C27H46O4S | 466.3117 | 22.08 | 0.02 | [M-H]- | 465.30 | 96.90 | 14 | 56 | 16 | 465.30 | 80.05 | 14 | 65 | 11 | 1 | 10000 | 0.7 | 0.994 |
| 140 | 22β-Hydroxycholesterol | 22-OHChol | C27H46O2 | 402.3498 | 22.08 | 0.02 | [M+H-H2O]+ | 385.35 | 257.30 | -17 | -19 | -17 |  |  |  |  |  | 400 | 4000 | 13.3 | 0.981 |
| 141 | 20α-Hydroxycholesterol | 20-OHChol | C27H46O2 | 402.3498 | 22.18 | 0.01 | [M+H-H2O]+ | 385.35 | 367.45 | -30 | -16 | -12 | 385.35 | 159.25 | -30 | -27 | -30 | 400 | 4000 | 6.4 | 0.992 |
| 142 | 7α-Hydroxycholesterol | 7-OHChol | C27H46O2 | 402.3498 | 22.98 | 0.01 | [M+H-H2O]+ | 385.35 | 367.20 | -13 | -14 | -17 | 385.35 | 159.30 | -17 | -28 | -10 | 1000 | 10000 | 1.3 | 0.919 |
| 143 | 7-Ketocholesterol | 7-keto-Chol | C27H44O2 | 400.3341 | 23.05 | 0.02 | [M+H]+ | 401.35 | 383.40 | -13 | -22 | -27 | 401.35 | 81.10 | -11 | -46 | -13 | 100 | 10000 | 6.6 | 0.817 |
| 144 | 4β-Hydroxycholesterol | 4-OHChol | C27H46O2 | 402.3498 | 24.03 | 0.01 | [M+H-H2O]+ | 385.35 | 109.20 | -17 | -25 | -22 | 385.35 | 367.20 | -13 | -14 | -17 | 100 | 1000 | 3.5 | 0.750 |
| 145 | Corticosterone-d8 | B-d8 (IS) | C21H22D8O4 | 354.2646 | - | - | [M+H]+ | 355.27 | 337.35 | -19 | -16 | -23 |  |  |  |  |  | - | - | - | - |
| 146 | 18-Hydroxycorticosterone-9,11,12,12-d4 | 18-OHB-d4 (IS) | C21H26O5D4 | 366.2344 | - | - | [M+H]+ | 367.24 | 273.30 | -10 | -17 | -13 |  |  |  |  |  | - | - | - | - |
| 147 | 11-Deoxycorticosterone-2,2,4,6,6,17α,21,21-d8 | DOC-d8 (IS) | C21H22O3D8 | 338.2697 | - | - | [M+H]+ | 339.28 | 100.20 | -29 | -24 | -15 |  |  |  |  |  | - | - | - | - |
| 148 | 11-Deoxycortisol-2,2,4,6,6-d5 | 11-DOF-d5 (IS) | C21H25O4D5 | 351.2458 | - | - | [M+H]+ | 352.25 | 100.20 | -16 | -25 | -17 |  |  |  |  |  | - | - | - | - |
| 149 | Cortisol-9,11,12,12-d4 | F-d4 (IS) | C21H26D4O5 | 366.2344 | - | - | [M+H]+ | 367.24 | 121.00 | -12 | -27 | -23 | 367.24 | 331.00 | -22 | -17 | -16 | - | - | - | - |
| 150 | Cortisone-2,2,4,6,6,9,12,12-d8 | E-d8 (IS) | C21H20D8O5 | 368.2439 | - | - | [M+H]+ | 369.25 | 168.20 | -16 | -25 | -28 |  |  |  |  |  | - | - | - | - |
| 151 | Testosterone-16,16,17-d3 | T-d3 (IS) | C19H25D3O2 | 291.2278 | - | - | [M+H]+ | 292.24 | 97.00 | -16 | -23 | -18 |  |  |  |  |  | - | - | - | - |
| 152 | Cholesterol sulfate-25,26,26,26,27,27,27-d7 | Chol-S-d7 (IS) | C27H39D7O4S | 473.3556 | - | - | [M+H]+ | 292.24 | 97.00 | -16 | -23 | -18 |  |  |  |  |  | - | - | - | - |
| 153 | Cholic acid-2,2,4,4-d4 | CA-d4 (IS) | C24 H36D4O5 | 412.3127 | - | - | [M-H]- | 411.31 | 411.30 | 11 | 21 | 14 |  |  |  |  |  | - | - | - | - |
| 154 | Chenodeoxycholic acid-2,2,4,4-d4 | CDCA-d4 (IS) | C24 H36D4O4 | 396.3178 | - | - | [M-H]- | 395.31 | 395.30 | 11 | 20 | 13 |  |  |  |  |  | - | - | - | - |
| 155 | Deoxycholic acid-2,2,4,4-d4 | DCA-d4 (IS) | C24H36D4O4 | 396.3178 | - | - | [M-H]- | 395.31 | 395.25 | 11 | 20 | 13 |  |  |  |  |  | - | - | - | - |
| 156 | Lithocholic acid-2,2,4,4-d4 | LCA-d4 (IS) | C24 H36D4O3 | 380.3229 | - | - | [M-H]- | 379.32 | 379.30 | 14 | 20 | 13 |  |  |  |  |  | - | - | - | - |
| 157 | Glycocholic acid-2,2,4,4-d4 | GCA-d4 (IS) | C26 H39D4NO6 | 469.3341 | - | - | [M-H]- | 468.33 | 468.30 | 11 | 17 | 16 |  |  |  |  |  | - | - | - | - |
| 158 | Glycodeoxycholic acid-2,2,4,4-d4 | GDCA-d4 (IS) | C26H39D4NO5 | 453.3392 | - | - | [M-H]- | 452.33 | 452.25 | 13 | 19 | 16 |  |  |  |  |  | - | - | - | - |
| 159 | Glycolithocholic acid-2,2,4,4-d4 | GLCA-d4 (IS) | C26H39D4NO4 | 437.3443 | - | - | [M-H]- | 436.34 | 436.30 | 10 | 15 | 15 |  |  |  |  |  | - | - | - | - |
| 160 | Taurocholic acid-2,2,4,5,5-d5 | TCA-d5 (IS) | C26H40D5NO7S | 520.3231 | - | - | [M-H]- | 519.32 | 519.25 | 20 | 28 | 24 |  |  |  |  |  | - | - | - | - |
| 161 | Taurolithocholic acid-2,2,4,5,5-d5 | TLCA-d5 (IS) | C26H40D5NO5S | 488.3332 | - | - | [M-H]- | 487.33 | 487.30 | 14 | 26 | 10 |  |  |  |  |  | - | - | - | - |
| 162 | Prostaglandin E2-3,3,4,4-d4 | PGE2-d4 (IS) | C20H28D4O5 | 356.2501 | - | - | [M-H]- | 355.24 | 275.25 | 10 | 18 | 12 |  |  |  |  |  | - | - | - | - |
| 163 | Prostaglandin D2-3,3,4,4,-d4 | PGD2-d4 (IS) | C20H28D4O5 | 356.2501 | - | - | [M-H]- | 355.24 | 275.40 | 16 | 19 | 26 |  |  |  |  |  | - | - | - | - |
| 164 | Prostaglandin F2α-3,3,4,4-d4 | PGF2α-d4 (IS) | C20H30D4O5 | 358.2657 | - | - | [M-H]- | 357.26 | 313.35 | 16 | 19 | 10 |  |  |  |  |  | - | - | - | - |
| 165 | Thromboxane B2-3,3,4,4-d4 | TXB2-d4 (IS) | C20H30D4O6 | 374.2606 | - | - | [M-H]- | 373.25 | 173.20 | 10 | 19 | 10 |  |  |  |  |  | - | - | - | - |
| 166 | Leukotriene D4-19,19,20,20,20-d5 | LTD4-d5 (IS) | C25H35D5N2O6S | 501.2921 | - | - | [M-H]- | 500.28 | 177.15 | 22 | 19 | 28 |  |  |  |  |  | - | - | - | - |
| 167 | Leukotriene B4-6,7,14,15-d4 | LTB4-d4 (IS) | C20H28D4O4 | 340.2552 | - | - | [M-H]- | 339.25 | 197.30 | 16 | 15 | 22 |  |  |  |  |  | - | - | - | - |
| 168 | 15(S)-Hydroxy-5Z,8Z,11Z,13E-eicosatetraenoic-5,6,8,9,11,12,14,15-d8 acid | 15-HETE-d8 (IS) | C20H24D8O3 | 328.2854 | - | - | [M-H]- | 327.28 | 226.20 | 10 | 13 | 10 |  |  |  |  |  | - | - | - | - |
| 169 | (S,5Z,8Z,11Z,13E,17Z)-15-Hydroxyicosa-5,8,11,13,17-pentaenoic-19,19,20,20,20-d5 acid | 15-HEPE-d5 (IS) | C20H25D5O3 | 323.2509 | - | - | [M-H]- | 322.25 | 260.25 | 21 | 13 | 28 |  |  |  |  |  | - | - | - | - |
| 170 | Docosahexaenoic acid-d5 | DHA-d5 (IS) | C22H27D5O2 | 333.2716 | - | - | [M-H]- | 332.25 | 288.35 | 12 | 12 | 30 |  |  |  |  |  | - | - | - | - |
| 171 | Arachidonic acid-d8 | AA-d8 (IS) | C20H24D8O2 | 312.2904 | - | - | [M-H]- | 311.30 | 267.35 | 20 | 14 | 18 |  |  |  |  |  | - | - | - | - |
| 172 | Eicosapentaenoic acid-d5 | EPA-d5 (IS) | C20H25D5O2 | 307.2560 | - | - | [M-H]- | 306.25 | 262.25 | 11 | 12 | 17 |  |  |  |  |  | - | - | - | - |

^a^LLOQ: lower limit of quantification tested.

^b^ULOQ: upper limit of quantification tested.

^c^Repeatability was calculated by measuring ULOQ injections (*n* = 3).

**Supplemental Table S6.** Information about the LC column used in this study.^a^

| Name | Hydrophobicity*^b^* | Steric selectivity*^c^* | Functional group | Carbon% | Surface area (m^2^/g) | Pore size (Å) | Pore volume (mL/g) | Column dimension | Particle size (μm) | pH availability |
| --- | --- | --- | --- | --- | --- | --- | --- | --- | --- | --- |
| Inertsil® ODS-4 | 5.9 | 1.5 | Octadecyl | 11% | 450 | 100 | 1.05 | 2.1 mm i.d. × 150 mm | 3 | 2–7.5 |
| InertSustain® C18 | 8.1 | 1.2 | Octadecyl | 14% | 350 | 100 | 0.85 | 2.1 mm i.d. × 150 mm | 3 | 1–10 |
| Inertsil® ODS-HL | 12.5 | 2.0 | Octadecyl | 29% | 450 | 100 | 1.05 | 2.1 mm i.d. × 150 mm | 3 | 2–7.5 |
| Inertsil® ODS-P | 7.1 | 2.4 | Octadecyl | 23% | 450 | 100 | 1.05 | 2.1 mm i.d. × 150 mm | 3 | 2–7.5 |

^a^Column information has been extracted and summarized from the vendor's website.

^b^Hydrophobicity was defined by the ratio of (k' amylbenzene/ k' butylbenzene) in the Tanaka method.

^c^Steric selectivity was defined by the ratio of (k' triphenylene/ k' o-terphenyl) in the Tanaka method.

**Supplemental Table S7.** Evaluation of the performance of each column on 128 bioactive lipid standards under LC-screening conditions.

|  | Column | Inertsil ODS-4 | | | | | | | | | | InertSustain C18 | | | | | | | | | | Inertsil ODS-HL | | | | | | | | | |
| --- | --- | --- | --- | --- | --- | --- | --- | --- | --- | --- | --- | --- | --- | --- | --- | --- | --- | --- | --- | --- | --- | --- | --- | --- | --- | --- | --- | --- | --- | --- | --- |
|  | Additive | Ammonium acetate | | | | | Acetic acid | | | | | Ammonium acetate | | | | | Acetic acid | | | | | Ammonium acetate | | | | | Acetic acid | | | | |
|  | Name | RT (min) | W_0.5_*^a^* (min) | S*^b^* | Intensity*^c^* | Area | RT (min) | W_0.5_*^a^* (min) | S*^b^* | Intensity*^c^* | Area | RT (min) | W_0.5_*^a^* (min) | S*^b^* | Intensity*^c^* | Area | RT (min) | W_0.5_*^a^* (min) | S*^b^* | Intensity*^c^* | Area | RT (min) | W_0.5_*^a^* (min) | S*^b^* | Intensity*^c^* | Area | RT (min) | W_0.5_*^a^* (min) | S*^b^* | Intensity*^c^* | Area |
| 1 | EPA | 10.57 | 0.08 | 1.116 | 8.E+06 | 1.E+06 | 11.48 | 0.09 | 1.128 | 1.E+08 | 2.E+07 | 10.31 | 0.09 | 1.267 | 7.E+06 | 1.E+06 | 11.49 | 0.09 | 1.102 | 6.E+07 | 9.E+06 | 11.12 | 0.08 | 1.208 | 1.E+07 | 2.E+06 | 11.97 | 0.08 | 1.122 | 7.E+07 | 1.E+07 |
| 2 | AA | 11.17 | 0.08 | 1.121 | 2.E+07 | 3.E+06 | 12.00 | 0.08 | 1.095 | 9.E+07 | 2.E+07 | 10.93 | 0.10 | 1.315 | 2.E+07 | 3.E+06 | 12.00 | 0.10 | 1.314 | 1.E+08 | 2.E+07 | 11.75 | 0.08 | 1.19 | 2.E+07 | 4.E+06 | 12.53 | 0.08 | 1.164 | 9.E+07 | 2.E+07 |
| 3 | 5-HETE | 9.13 | 0.07 | 1.085 | 2.E+06 | 5.E+05 | 10.13 | 0.07 | 1.088 | 4.E+07 | 9.E+06 | 8.82 | 0.08 | 1.214 | 2.E+06 | 3.E+05 | 10.07 | 0.09 | 1.314 | 1.E+07 | 2.E+06 | 9.48 | 0.08 | 1.257 | 8.E+06 | 2.E+06 | 10.52 | 0.08 | 1.199 | 2.E+07 | 4.E+06 |
| 4 | 12-HETE | 8.79 | 0.07 | 1.08 | 2.E+06 | 5.E+05 | 9.83 | 0.07 | 1.093 | 1.E+07 | 3.E+06 | 8.43 | 0.08 | 1.214 | 2.E+06 | 4.E+05 | 9.75 | 0.09 | 1.353 | 1.E+07 | 3.E+06 | 9.13 | 0.08 | 1.378 | 3.E+06 | 6.E+05 | 10.13 | 0.07 | 1.172 | 1.E+07 | 3.E+06 |
| 5 | DHA | 10.96 | 0.08 | 1.089 | 1.E+07 | 2.E+06 | 11.91 | 0.08 | 1.047 | 7.E+07 | 1.E+07 | 10.71 | 0.10 | 1.282 | 9.E+06 | 1.E+06 | 11.90 | 0.10 | 1.359 | 7.E+07 | 1.E+07 | 11.50 | 0.09 | 1.241 | 2.E+07 | 3.E+06 | 12.41 | 0.08 | 1.187 | 8.E+07 | 1.E+07 |
| 6 | PGA2 | 4.97 | 0.07 | 1.122 | 8.E+05 | 2.E+05 | 6.80 | 0.07 | 1.109 | 3.E+07 | 6.E+06 | 4.48 | 0.08 | 1.112 | 7.E+05 | 2.E+05 | 6.73 | 0.07 | 1.128 | 5.E+06 | 1.E+06 | 5.17 | 0.08 | 1.181 | 9.E+05 | 2.E+05 | 6.92 | 0.07 | 1.219 | 1.E+07 | 2.E+06 |
| 7 | PGB2 | 5.20 | 0.07 | 1.016 | 1.E+06 | 2.E+05 | 6.92 | 0.06 | 1.073 | 8.E+06 | 2.E+06 | 4.72 | 0.08 | 1.103 | 9.E+05 | 2.E+05 | 6.83 | 0.07 | 1.173 | 1.E+07 | 2.E+06 | 5.39 | 0.08 | 1.16 | 1.E+06 | 2.E+05 | 7.03 | 0.07 | 1.285 | 8.E+06 | 2.E+06 |
| 8 | 5-HpETE | 9.15 | 0.07 | 1.194 | 1.E+05 | 3.E+04 | 10.14 | 0.07 | 0.995 | 1.E+06 | 3.E+05 | 8.85 | 0.09 | 1.303 | 7.E+04 | 1.E+04 | 10.06 | 0.09 | 1.38 | 7.E+05 | 1.E+05 | 9.50 | 0.07 | 1.143 | 5.E+05 | 1.E+05 | 10.48 | 0.08 | 1.183 | 8.E+05 | 2.E+05 |
| 9 | 12-HpETE | 8.84 | 0.07 | 1.288 | 3.E+05 | 7.E+04 | 9.81 | 0.07 | 1.066 | 3.E+06 | 6.E+05 | 8.51 | 0.08 | 1.171 | 3.E+05 | 6.E+04 | 9.73 | 0.08 | 1.283 | 2.E+05 | 4.E+04 | 9.17 | 0.08 | 1.089 | 4.E+05 | 8.E+04 | 10.08 | 0.07 | 1.212 | 2.E+06 | 5.E+05 |
| 10 | 15-HpETE | 8.61 | 0.07 | 1.164 | 8.E+05 | 2.E+05 | 9.61 | 0.07 | 1.094 | 2.E+07 | 3.E+06 | 8.25 | 0.07 | 1.186 | 8.E+05 | 2.E+05 | 9.52 | 0.08 | 1.256 | 6.E+06 | 1.E+06 | 8.91 | 0.07 | 1.303 | 1.E+06 | 2.E+05 | 9.85 | 0.07 | 1.206 | 7.E+06 | 2.E+06 |
| 11 | LTB4 | 6.59 | 0.07 | 1.103 | 1.E+06 | 3.E+05 | 7.80 | 0.07 | 1.118 | 1.E+07 | 3.E+06 | 6.24 | 0.07 | 1.118 | 1.E+06 | 3.E+05 | 7.75 | 0.08 | 1.277 | 1.E+07 | 3.E+06 | 6.79 | 0.07 | 1.243 | 1.E+06 | 3.E+05 | 8.02 | 0.07 | 1.302 | 1.E+07 | 3.E+06 |
| 12 | 17-HDoHE | 8.42 | 0.07 | 1.086 | 7.E+05 | 2.E+05 | 9.56 | 0.07 | 1.115 | 1.E+07 | 3.E+06 | 8.07 | 0.08 | 1.069 | 5.E+05 | 1.E+05 | 9.49 | 0.09 | 1.29 | 4.E+06 | 7.E+05 | 8.72 | 0.07 | 1.187 | 3.E+06 | 6.E+05 | 9.84 | 0.07 | 1.257 | 5.E+06 | 1.E+06 |
| 13 | PGK2 | 1.50 | 0.04 | 1.283 | 3.E+06 | 1.E+06 | 5.73 | 0.08 | 1.378 | 6.E+07 | 1.E+07 | 1.44 | 0.04 | 1.181 | 2.E+06 | 8.E+05 | 5.78 | 0.18 | 3.909 | 2.E+07 | 1.E+06 | 1.39 | 0.04 | 1.46 | 3.E+06 | 1.E+06 | 5.91 | 0.09 | 1.77 | 2.E+07 | 3.E+06 |
| 14 | RvE1 | 1.96 | 0.06 | 1.157 | 5.E+05 | 1.E+05 | 3.49 | 0.06 | 1.125 | 4.E+06 | 9.E+05 | 1.74 | 0.06 | 1.173 | 6.E+05 | 1.E+05 | 3.55 | 0.07 | 1.144 | 4.E+06 | 9.E+05 | 1.80 | 0.06 | 1.345 | 7.E+05 | 2.E+05 | 3.43 | 0.08 | 1.568 | 4.E+06 | 7.E+05 |
| 15 | PGD2 | 3.29 | 0.07 | 1.122 | 3.E+06 | 7.E+05 | 5.71 | 0.07 | 1.101 | 5.E+07 | 1.E+07 | 2.89 | 0.07 | 1.204 | 2.E+06 | 5.E+05 | 5.70 | 0.07 | 1.163 | 2.E+07 | 4.E+06 | 3.26 | 0.08 | 1.147 | 1.E+07 | 2.E+06 | 5.84 | 0.07 | 1.345 | 3.E+07 | 5.E+06 |
| 16 | PGE2 | 2.88 | 0.06 | 1.171 | 4.E+06 | 1.E+06 | 5.48 | 0.07 | 1.118 | 3.E+07 | 7.E+06 | 2.58 | 0.05 | 1.132 | 4.E+06 | 1.E+06 | 5.51 | 0.07 | 1.144 | 3.E+07 | 7.E+06 | 2.84 | 0.07 | 1.239 | 5.E+06 | 1.E+06 | 5.59 | 0.07 | 1.312 | 3.E+07 | 6.E+06 |
| 17 | PGH2 | 2.88 | 0.06 | 1.171 | 1.E+06 | 2.E+05 | 5.48 | 0.07 | 1.076 | 1.E+07 | 2.E+06 | 2.59 | 0.05 | 1.15 | 1.E+06 | 3.E+05 | 5.51 | 0.07 | 1.113 | 9.E+06 | 2.E+06 | 2.84 | 0.08 | 1.194 | 1.E+06 | 3.E+05 | 5.59 | 0.07 | 1.304 | 1.E+07 | 2.E+06 |
| 18 | LXA4 | 4.78 | 0.06 | 1.051 | 1.E+06 | 2.E+05 | 6.23 | 0.07 | 1.088 | 1.E+07 | 3.E+06 | 4.37 | 0.08 | 1.154 | 9.E+05 | 2.E+05 | 6.22 | 0.08 | 1.125 | 1.E+07 | 2.E+06 | 4.85 | 0.07 | 1.196 | 1.E+06 | 2.E+05 | 6.37 | 0.07 | 1.33 | 1.E+07 | 2.E+06 |
| 19 | LXB4 | 3.57 | 0.06 | 1.123 | 6.E+05 | 1.E+05 | 5.62 | 0.07 | 1.102 | 3.E+07 | 6.E+06 | 3.20 | 0.06 | 1.085 | 6.E+05 | 1.E+05 | 5.63 | 0.07 | 1.187 | 6.E+06 | 1.E+06 | 3.50 | 0.08 | 1.199 | 6.E+05 | 1.E+05 | 5.75 | 0.07 | 1.309 | 1.E+07 | 2.E+06 |
| 20 | 15-epi-LXA4 | 4.75 | 0.07 | 1.093 | 1.E+06 | 2.E+05 | 6.25 | 0.06 | 1.07 | 1.E+07 | 3.E+06 | 4.36 | 0.08 | 1.092 | 1.E+06 | 2.E+05 | 6.24 | 0.07 | 1.164 | 2.E+07 | 3.E+06 | 4.84 | 0.08 | 1.308 | 1.E+06 | 2.E+05 | 6.41 | 0.07 | 1.232 | 1.E+07 | 3.E+06 |
| 21 | PGF2α | 2.68 | 0.05 | 1.161 | 3.E+06 | 8.E+05 | 5.31 | 0.07 | 1.057 | 2.E+07 | 4.E+06 | 2.44 | 0.05 | 1.17 | 1.E+06 | 3.E+05 | 5.37 | 0.08 | 1.125 | 1.E+07 | 2.E+06 | 2.63 | 0.05 | 1.364 | 8.E+06 | 2.E+06 | 5.46 | 0.08 | 1.403 | 1.E+07 | 2.E+06 |
| 22 | PGE1 | 3.09 | 0.07 | 1.106 | 7.E+06 | 1.E+06 | 5.64 | 0.06 | 1.1 | 5.E+07 | 1.E+07 | 2.69 | 0.06 | 1.136 | 6.E+06 | 2.E+06 | 5.65 | 0.07 | 1.102 | 4.E+06 | 1.E+06 | 3.11 | 0.08 | 1.247 | 7.E+06 | 1.E+06 | 5.75 | 0.07 | 1.288 | 5.E+07 | 1.E+07 |
| 23 | Maresin-1 | 6.16 | 0.07 | 1.348 | 3.E+05 | 8.E+04 | 7.56 | 0.07 | 1.168 | 2.E+06 | 6.E+05 | 5.79 | 0.07 | 1.084 | 3.E+05 | 7.E+04 | 7.50 | 0.07 | 1.202 | 3.E+06 | 6.E+05 | 6.32 | 0.07 | 1.29 | 4.E+05 | 8.E+04 | 7.71 | 0.07 | 1.385 | 3.E+06 | 6.E+05 |
| 24 | Protectin D1 | 6.04 | 0.07 | 1.095 | 2.E+06 | 6.E+05 | 7.53 | 0.06 | 1.104 | 2.E+07 | 4.E+06 | 5.65 | 0.07 | 1.116 | 2.E+06 | 5.E+05 | 7.47 | 0.07 | 1.164 | 1.E+07 | 3.E+06 | 6.24 | 0.07 | 1.271 | 3.E+06 | 6.E+05 | 7.67 | 0.07 | 1.251 | 2.E+07 | 4.E+06 |
| 25 | PGG2 | 3.84 | 0.08 | 1.044 | 4.E+06 | 7.E+05 | 6.00 | 0.07 | 1.136 | 2.E+07 | 4.E+06 | 3.37 | 0.07 | 1.096 | 3.E+06 | 7.E+05 | 5.99 | 0.08 | 1.143 | 2.E+07 | 4.E+06 | 3.96 | 0.09 | 1.125 | 4.E+06 | 7.E+05 | 6.09 | 0.07 | 1.267 | 2.E+07 | 5.E+06 |
| 26 | TXB2 | 2.34 | 0.05 | 3.86 | 4.E+06 | 6.E+05 | 4.63 | 0.08 | 7.916 | 2.E+08 | 2.E+07 | 2.06 | 0.06 | 1.196 | 2.E+06 | 5.E+05 | 4.74 | 0.08 | 7.131 | 4.E+07 | 3.E+06 | 2.23 | 0.07 | 3.881 | 4.E+06 | 5.E+05 | 4.76 | 0.09 | 7.064 | 7.E+07 | 5.E+06 |
| 27 | RvD1 | 4.55 | 0.07 | 1.01 | 7.E+05 | 2.E+05 | 6.14 | 0.06 | 1.141 | 7.E+06 | 2.E+06 | 4.15 | 0.07 | 1.059 | 7.E+05 | 2.E+05 | 6.12 | 0.07 | 1.201 | 9.E+06 | 2.E+06 | 4.61 | 0.08 | 1.126 | 8.E+05 | 2.E+05 | 6.25 | 0.07 | 1.283 | 7.E+06 | 1.E+06 |
| 28 | RvD2 | 3.52 | 0.06 | 1.126 | 3.E+06 | 7.E+05 | 5.67 | 0.06 | 1.126 | 1.E+07 | 4.E+06 | 3.15 | 0.07 | 1.152 | 1.E+06 | 3.E+05 | 5.66 | 0.07 | 1.106 | 9.E+06 | 2.E+06 | 3.46 | 0.07 | 1.137 | 9.E+06 | 2.E+06 | 5.76 | 0.07 | 1.277 | 1.E+07 | 2.E+06 |
| 29 | RvD3 | 3.73 | 0.07 | 1.181 | 2.E+06 | 5.E+05 | 5.50 | 0.06 | 1.124 | 2.E+07 | 4.E+06 | 3.38 | 0.07 | 1.181 | 2.E+06 | 5.E+05 | 5.52 | 0.07 | 1.094 | 2.E+06 | 4.E+05 | 3.68 | 0.08 | 1.229 | 2.E+06 | 4.E+05 | 5.60 | 0.07 | 1.297 | 2.E+07 | 4.E+06 |
| 30 | RvD4 | 5.54 | 0.06 | 1.166 | 1.E+06 | 3.E+05 | 6.73 | 0.07 | 1.134 | 3.E+07 | 6.E+06 | 5.21 | 0.07 | 1.187 | 1.E+06 | 3.E+05 | 6.71 | 0.08 | 1.19 | 9.E+06 | 2.E+06 | 5.65 | 0.07 | 1.097 | 1.E+06 | 3.E+05 | 6.89 | 0.07 | 1.281 | 1.E+07 | 2.E+06 |
| 31 | LTE4 | 6.59 | 0.20 | 5.739 | 1.E+06 | 3.E+04 | 7.53 | 0.13 | 4.187 | 2.E+07 | 1.E+06 | ----- | ----- | ----- | ----- | ----- | 8.25 | 1.29 | 4.541 | 1.E+07 | 1.E+05 | 6.76 | 0.27 | 4.675 | 1.E+06 | 5.E+04 | 7.81 | 0.10 | 3.409 | 2.E+07 | 2.E+06 |
| 32 | LTD4 | 5.89 | 0.17 | 5.378 | 2.E+06 | 1.E+05 | 6.95 | 0.10 | 2.804 | 6.E+07 | 7.E+06 | 5.77 | 0.54 | 7.217 | 2.E+06 | 2.E+04 | 7.35 | 0.46 | 7.292 | 2.E+07 | 4.E+05 | 6.06 | 0.12 | 3.818 | 8.E+06 | 7.E+05 | 7.05 | 0.09 | 2.405 | 3.E+07 | 3.E+06 |
| 33 | LTC4 | ----- | ----- | ----- | ----- | ----- | ----- | ----- | ----- | ----- | ----- | ----- | ----- | ----- | ----- | ----- | ----- | ----- | ----- | ----- | ----- | ----- | ----- | ----- | ----- | ----- | 8.10 | 0.22 | 3.84 | 1.E+06 | 1.E+04 |
| 34 | LCA | 9.09 | 0.08 | 1.084 | 2.E+07 | 4.E+06 | 10.00 | 0.09 | 1.112 | 1.E+08 | 2.E+07 | 8.63 | 0.08 | 1.18 | 2.E+07 | 3.E+06 | 9.82 | 0.08 | 1.169 | 1.E+08 | 2.E+07 | 9.54 | 0.07 | 1.27 | 2.E+07 | 4.E+06 | 10.39 | 0.09 | 1.159 | 1.E+08 | 2.E+07 |
| 35 | Allo-LCA | 9.20 | 0.07 | 1.123 | 1.E+07 | 3.E+06 | 10.14 | 0.07 | 1.132 | 7.E+07 | 2.E+07 | 8.38 | 0.08 | 1.095 | 2.E+07 | 5.E+06 | 9.92 | 0.13 | 1.605 | 2.E+08 | 2.E+07 | 9.75 | 0.09 | 1.217 | 1.E+07 | 3.E+06 | 10.61 | 0.08 | 1.266 | 6.E+07 | 1.E+07 |
| 36 | 7-keto-LCA | 5.83 | 0.07 | 1.138 | 1.E+07 | 2.E+06 | 7.43 | 0.07 | 1.109 | 8.E+07 | 2.E+07 | 5.26 | 0.08 | 1.161 | 9.E+06 | 2.E+06 | 7.31 | 0.09 | 1.172 | 1.E+08 | 2.E+07 | 6.22 | 0.07 | 1.214 | 1.E+07 | 3.E+06 | 7.65 | 0.07 | 1.237 | 9.E+07 | 2.E+07 |
| 37 | 12-keto-LCA | 6.01 | 0.07 | 1.137 | 1.E+07 | 2.E+06 | 7.66 | 0.06 | 1.091 | 5.E+07 | 1.E+07 | 5.46 | 0.07 | 1.069 | 9.E+06 | 2.E+06 | 7.52 | 0.07 | 1.194 | 7.E+07 | 2.E+07 | 6.41 | 0.07 | 1.248 | 3.E+07 | 7.E+06 | 7.89 | 0.07 | 1.218 | 8.E+07 | 2.E+07 |
| 38 | UDCA | 5.27 | 0.08 | 1.083 | 5.E+06 | 1.E+06 | 7.13 | 0.07 | 1.107 | 5.E+07 | 1.E+07 | 4.59 | 0.09 | 1.083 | 5.E+06 | 8.E+05 | 7.03 | 0.07 | 1.187 | 5.E+07 | 1.E+07 | 5.67 | 0.08 | 1.151 | 6.E+06 | 1.E+06 | 7.32 | 0.07 | 1.235 | 5.E+07 | 1.E+07 |
| 39 | HDCA | 5.48 | 0.08 | 1.133 | 5.E+06 | 1.E+06 | 7.33 | 0.08 | 1.084 | 1.E+08 | 2.E+07 | 4.79 | 0.09 | 1.023 | 4.E+06 | 7.E+05 | 7.22 | 0.08 | 1.223 | 5.E+07 | 1.E+07 | 5.91 | 0.08 | 1.208 | 6.E+06 | 1.E+06 | 7.53 | 0.07 | 1.259 | 6.E+07 | 1.E+07 |
| 40 | CDCA | 7.30 | 0.07 | 1.068 | 5.E+06 | 1.E+06 | 8.57 | 0.07 | 1.056 | 8.E+07 | 2.E+07 | 6.81 | 0.08 | 1.102 | 5.E+06 | 9.E+05 | 8.42 | 0.08 | 1.171 | 5.E+07 | 8.E+06 | 7.71 | 0.08 | 1.254 | 6.E+06 | 1.E+06 | 8.85 | 0.07 | 1.184 | 5.E+07 | 9.E+06 |
| 41 | Allo-CDCA | 7.34 | 0.07 | 1.11 | 6.E+06 | 1.E+06 | 8.61 | 0.07 | 1.075 | 7.E+07 | 2.E+07 | 6.84 | 0.08 | 1.119 | 6.E+06 | 1.E+06 | 8.48 | 0.08 | 1.21 | 5.E+07 | 1.E+07 | 7.75 | 0.07 | 1.249 | 7.E+06 | 1.E+06 | 8.90 | 0.07 | 1.228 | 5.E+07 | 1.E+07 |
| 42 | DCA | 7.55 | 0.07 | 1.077 | 2.E+07 | 5.E+06 | 8.78 | 0.14 | 1.624 | 2.E+08 | 2.E+07 | 7.08 | 0.08 | 1.095 | 2.E+07 | 5.E+06 | 8.64 | 0.17 | 2.247 | 2.E+08 | 2.E+07 | 7.94 | 0.08 | 1.268 | 3.E+07 | 5.E+06 | 9.08 | 0.12 | 1.677 | 2.E+08 | 2.E+07 |
| 43 | 7-keto-DCA | 2.96 | 0.07 | 1.146 | 7.E+06 | 1.E+06 | 5.78 | 0.07 | 1.088 | 9.E+07 | 2.E+07 | 2.60 | 0.06 | 1.146 | 6.E+06 | 2.E+06 | 5.76 | 0.08 | 1.154 | 1.E+08 | 2.E+07 | 3.04 | 0.09 | 1.14 | 7.E+06 | 1.E+06 | 5.97 | 0.07 | 1.308 | 8.E+07 | 2.E+07 |
| 44 | ω-MCA | 2.58 | 0.05 | 1.19 | 2.E+06 | 6.E+05 | 5.71 | 0.07 | 1.078 | 2.E+07 | 5.E+06 | 2.31 | 0.06 | 1.094 | 2.E+06 | 4.E+05 | 5.72 | 0.08 | 1.118 | 2.E+07 | 5.E+06 | 2.55 | 0.06 | 1.345 | 6.E+06 | 2.E+06 | 5.88 | 0.08 | 1.321 | 2.E+07 | 5.E+06 |
| 45 | α-MCA | 2.74 | 0.06 | 1.113 | 2.E+06 | 5.E+05 | 5.89 | 0.07 | 1.129 | 2.E+07 | 3.E+06 | 2.45 | 0.05 | 1.151 | 2.E+06 | 5.E+05 | 5.89 | 0.08 | 1.112 | 2.E+07 | 3.E+06 | 2.75 | 0.07 | 1.256 | 2.E+06 | 5.E+05 | 6.05 | 0.08 | 1.236 | 2.E+07 | 3.E+06 |
| 46 | β-MCA | 3.03 | 0.08 | 1.142 | 3.E+06 | 7.E+05 | 6.15 | 0.08 | 1.177 | 1.E+08 | 2.E+07 | 2.61 | 0.06 | 1.091 | 3.E+06 | 9.E+05 | 6.11 | 0.07 | 1.11 | 4.E+07 | 8.E+06 | 3.17 | 0.10 | 1.182 | 4.E+06 | 6.E+05 | 6.33 | 0.07 | 1.287 | 4.E+07 | 9.E+06 |
| 47 | CA | 4.99 | 0.08 | 1.11 | 6.E+06 | 1.E+06 | 7.14 | 0.10 | 1.229 | 1.E+08 | 2.E+07 | 4.32 | 0.10 | 1.066 | 6.E+06 | 1.E+06 | 7.08 | 0.08 | 1.154 | 8.E+07 | 2.E+07 | 5.45 | 0.09 | 1.162 | 8.E+06 | 1.E+06 | 7.40 | 0.07 | 1.188 | 8.E+07 | 2.E+07 |
| 48 | GLCA | 7.31 | 0.06 | 1.149 | 2.E+07 | 5.E+06 | 8.57 | 0.11 | 1.422 | 1.E+08 | 2.E+07 | 6.99 | 0.07 | 1.145 | 2.E+07 | 4.E+06 | 8.39 | 0.13 | 1.66 | 2.E+08 | 2.E+07 | 7.46 | 0.07 | 1.28 | 2.E+07 | 5.E+06 | 8.90 | 0.11 | 1.489 | 1.E+08 | 2.E+07 |
| 49 | GHDCA | 3.52 | 0.07 | 1.133 | 1.E+07 | 3.E+06 | 5.41 | 0.08 | 1.147 | 1.E+08 | 2.E+07 | 3.10 | 0.08 | 1.146 | 1.E+07 | 3.E+06 | 5.41 | 0.09 | 1.352 | 1.E+08 | 2.E+07 | 3.38 | 0.08 | 1.122 | 5.E+07 | 1.E+07 | 5.60 | 0.09 | 1.35 | 1.E+08 | 2.E+07 |
| 50 | GUDCA | 3.58 | 0.07 | 1.141 | 9.E+06 | 2.E+06 | 5.33 | 0.07 | 1.147 | 8.E+07 | 2.E+07 | 3.19 | 0.07 | 1.131 | 1.E+07 | 2.E+06 | 5.34 | 0.09 | 1.222 | 8.E+07 | 1.E+07 | 3.47 | 0.08 | 1.146 | 1.E+07 | 2.E+06 | 5.54 | 0.08 | 1.318 | 8.E+07 | 2.E+07 |
| 51 | GCDCA | 5.75 | 0.07 | 1.151 | 1.E+07 | 3.E+06 | 7.04 | 0.13 | 1.823 | 2.E+08 | 2.E+07 | 5.39 | 0.07 | 1.083 | 1.E+07 | 2.E+06 | 6.98 | 0.09 | 1.383 | 1.E+08 | 2.E+07 | 5.87 | 0.07 | 1.225 | 1.E+07 | 3.E+06 | 7.36 | 0.09 | 1.37 | 1.E+08 | 2.E+07 |
| 52 | GDCA | 6.18 | 0.07 | 1.131 | 1.E+07 | 3.E+06 | 7.42 | 0.11 | 1.581 | 1.E+08 | 2.E+07 | 5.84 | 0.07 | 1.156 | 1.E+07 | 2.E+06 | 7.34 | 0.10 | 1.405 | 1.E+08 | 2.E+07 | 6.31 | 0.07 | 1.237 | 1.E+07 | 3.E+06 | 7.76 | 0.08 | 1.224 | 1.E+08 | 2.E+07 |
| 53 | GCA | 3.83 | 0.08 | 1.093 | 9.E+06 | 2.E+06 | 5.42 | 0.11 | 1.453 | 1.E+08 | 2.E+07 | 3.41 | 0.08 | 1.161 | 1.E+07 | 2.E+06 | 5.51 | 0.12 | 1.484 | 1.E+08 | 1.E+07 | 3.87 | 0.10 | 0.982 | 1.E+07 | 2.E+06 | 5.77 | 0.09 | 1.445 | 1.E+08 | 2.E+07 |
| 54 | TLCA | 7.43 | 0.06 | 1.208 | 1.E+07 | 4.E+06 | 6.87 | 0.13 | 1.897 | 2.E+08 | 2.E+07 | 7.10 | 0.07 | 1.174 | 2.E+07 | 4.E+06 | 6.46 | 0.18 | 2.809 | 2.E+08 | 2.E+07 | 7.54 | 0.07 | 1.272 | 2.E+07 | 4.E+06 | 8.14 | 0.14 | 1.352 | 2.E+08 | 2.E+07 |
| 55 | THDCA | 4.13 | 0.07 | 1.033 | 1.E+07 | 3.E+06 | 2.77 | 0.09 | 1.335 | 1.E+08 | 2.E+07 | 3.67 | 0.07 | 1.08 | 1.E+07 | 3.E+06 | 2.61 | 0.11 | 1.65 | 1.E+08 | 2.E+07 | 4.08 | 0.08 | 1.092 | 1.E+07 | 3.E+06 | 4.20 | 0.16 | 1.302 | 1.E+08 | 1.E+07 |
| 56 | TUDCA | 4.13 | 0.07 | 1.062 | 1.E+07 | 2.E+06 | 2.87 | 0.08 | 1.337 | 9.E+07 | 2.E+07 | 3.69 | 0.07 | 1.15 | 1.E+07 | 2.E+06 | 2.68 | 0.10 | 1.55 | 9.E+07 | 1.E+07 | 4.09 | 0.08 | 1.143 | 4.E+07 | 7.E+06 | 4.32 | 0.15 | 1.252 | 1.E+08 | 1.E+07 |
| 57 | TCDCA | 5.99 | 0.06 | 1.088 | 1.E+07 | 3.E+06 | 5.05 | 0.10 | 1.294 | 1.E+08 | 2.E+07 | 5.66 | 0.07 | 1.121 | 1.E+07 | 3.E+06 | 4.82 | 0.12 | 1.37 | 1.E+08 | 2.E+07 | 6.10 | 0.07 | 1.218 | 2.E+07 | 3.E+06 | 6.50 | 0.13 | 1.295 | 2.E+08 | 2.E+07 |
| 58 | TDCA | 6.37 | 0.06 | 1.151 | 2.E+07 | 4.E+06 | 5.50 | 0.16 | 1.94 | 2.E+08 | 2.E+07 | 6.04 | 0.07 | 1.171 | 2.E+07 | 4.E+06 | 5.25 | 0.12 | 1.529 | 2.E+08 | 2.E+07 | 6.48 | 0.07 | 1.243 | 2.E+07 | 5.E+06 | 6.94 | 0.16 | 1.498 | 2.E+08 | 2.E+07 |
| 59 | TαMCA | 2.56 | 0.04 | 1.173 | 9.E+06 | 3.E+06 | 1.88 | 0.09 | 1.386 | 1.E+08 | 2.E+07 | 2.32 | 0.05 | 1.134 | 9.E+06 | 3.E+06 | 1.75 | 0.09 | 1.576 | 7.E+07 | 1.E+07 | 2.37 | 0.05 | 1.399 | 1.E+07 | 3.E+06 | 2.41 | 0.10 | 1.584 | 8.E+07 | 1.E+07 |
| 60 | TβMCA | 2.66 | 0.04 | 1.167 | 7.E+06 | 3.E+06 | 1.97 | 0.08 | 1.393 | 1.E+08 | 2.E+07 | 2.39 | 0.05 | 1.125 | 8.E+06 | 3.E+06 | 1.84 | 0.09 | 1.708 | 6.E+07 | 1.E+07 | 2.45 | 0.05 | 1.447 | 9.E+06 | 3.E+06 | 2.54 | 0.10 | 1.548 | 7.E+07 | 1.E+07 |
| 61 | TCA | 4.35 | 0.07 | 1.074 | 9.E+06 | 2.E+06 | 2.87 | 0.13 | 1.711 | 2.E+08 | 2.E+07 | 3.90 | 0.07 | 1.088 | 1.E+07 | 2.E+06 | 2.74 | 0.21 | 2.755 | 3.E+08 | 2.E+07 | 4.42 | 0.08 | 1.104 | 1.E+07 | 2.E+06 | 4.61 | 0.17 | 1.245 | 1.E+08 | 1.E+07 |
| 62 | E1 | 6.83 | 0.06 | 1.068 | 1.E+04 | 4.E+03 | 6.47 | 0.04 | 1.18 | 7.E+03 | 1.E+03 | 6.51 | 0.05 | 1.561 | 2.E+04 | 5.E+03 | ----- | ----- | ----- | ----- | ----- | 7.00 | 0.05 | 0.961 | 1.E+04 | 3.E+03 | ----- | ----- | ----- | ----- | ----- |
| 63 | 16α-OHE1 | 4.65 | 0.07 | 0.881 | 3.E+04 | 7.E+03 | 4.10 | 0.05 | 1.019 | 2.E+04 | 6.E+03 | 4.35 | 0.07 | 1.051 | 6.E+04 | 1.E+04 | 4.04 | 0.07 | 1.239 | 1.E+04 | 2.E+03 | 4.70 | 0.07 | 1.36 | 3.E+04 | 7.E+03 | 4.10 | 0.06 | 1.267 | 1.E+04 | 3.E+03 |
| 64 | 2-OHE1 | 5.82 | 0.08 | 1.868 | 3.E+06 | 5.E+05 | 5.36 | 0.07 | 1.043 | 1.E+07 | 2.E+06 | 5.55 | 0.10 | 1.091 | 2.E+06 | 2.E+05 | 5.27 | 0.07 | 1.187 | 6.E+06 | 1.E+06 | 5.94 | 0.14 | 1.819 | 2.E+06 | 2.E+05 | 5.46 | 0.08 | 1.029 | 1.E+07 | 2.E+06 |
| 65 | A4 | 6.68 | 0.07 | 1.12 | 3.E+06 | 6.E+05 | 6.34 | 0.06 | 1.049 | 8.E+06 | 2.E+06 | 6.37 | 0.07 | 1.12 | 2.E+06 | 5.E+05 | 6.17 | 0.07 | 1.104 | 2.E+07 | 4.E+06 | 6.82 | 0.07 | 1.384 | 3.E+06 | 7.E+05 | 6.49 | 0.07 | 1.22 | 1.E+07 | 3.E+06 |
| 66 | E3 | 3.40 | 0.04 | 1.028 | 5.E+03 | 2.E+03 | ----- | ----- | ----- | ----- | ----- | 3.15 | 0.05 | 1.135 | 8.E+03 | 3.E+03 | ----- | ----- | ----- | ----- | ----- | 3.38 | 0.05 | 1.163 | 5.E+03 | 2.E+03 | 2.72 | 0.05 | 1.182 | 5.E+03 | 1.E+03 |
| 67 | 2-OHE2 | 5.51 | 0.10 | 2.609 | 2.E+05 | 2.E+04 | 4.93 | 0.07 | 1.009 | 7.E+05 | 1.E+05 | 5.20 | 0.10 | 1.556 | 9.E+04 | 9.E+03 | 4.87 | 0.07 | 1.075 | 3.E+05 | 7.E+04 | 5.66 | 0.12 | 2.347 | 2.E+05 | 1.E+04 | 5.07 | 0.08 | 1.174 | 8.E+05 | 1.E+05 |
| 68 | ED | 9.79 | 0.08 | 1.061 | 6.E+06 | 1.E+06 | 9.54 | 0.08 | 1.003 | 2.E+06 | 5.E+05 | 9.61 | 0.09 | 1.215 | 7.E+06 | 1.E+06 | 9.47 | 0.09 | 1.134 | 8.E+06 | 1.E+06 | 10.24 | 0.10 | 1.249 | 2.E+07 | 4.E+06 | 10.01 | 0.08 | 1.167 | 8.E+06 | 1.E+06 |
| 69 | AD | 9.78 | 0.08 | 1.038 | 2.E+06 | 3.E+05 | 9.53 | 0.08 | 0.97 | 1.E+06 | 2.E+05 | 9.57 | 0.09 | 1.154 | 2.E+06 | 3.E+05 | 9.41 | 0.08 | 1.192 | 2.E+06 | 4.E+05 | 10.32 | 0.10 | 1.252 | 2.E+06 | 3.E+05 | 10.08 | 0.08 | 1.209 | 2.E+06 | 5.E+05 |
| 70 | T | 6.66 | 0.07 | 1.065 | 1.E+07 | 3.E+06 | 6.24 | 0.07 | 1.047 | 1.E+07 | 3.E+06 | 6.27 | 0.08 | 1.114 | 1.E+07 | 3.E+06 | 6.02 | 0.08 | 1.093 | 3.E+07 | 6.E+06 | 6.87 | 0.08 | 1.249 | 2.E+07 | 5.E+06 | 6.45 | 0.08 | 1.208 | 3.E+07 | 5.E+06 |
| 71 | 5β-DHT | 9.76 | 0.09 | 0.785 | 1.E+05 | 3.E+04 | 9.51 | 0.06 | 0.898 | 5.E+04 | 2.E+04 | 9.52 | 0.08 | 0.744 | 2.E+05 | 3.E+04 | 9.39 | 0.08 | 0.919 | 2.E+05 | 3.E+04 | 10.25 | 0.10 | 1.04 | 9.E+04 | 2.E+04 | 10.01 | 0.09 | 1.307 | 1.E+05 | 3.E+04 |
| 72 | DHT | ----- | ----- | ----- | ----- | ----- | ----- | ----- | ----- | ----- | ----- | 9.44 | 0.06 | 0.886 | 2.E+04 | 7.E+03 | 9.93 | 0.05 | 0.85 | 3.E+04 | 1.E+04 | 10.30 | 0.11 | 0.982 | 3.E+04 | 6.E+03 | 10.25 | 0.15 | 1.451 | 4.E+05 | 1.E+04 |
| 73 | An | 8.20 | 0.09 | 1.098 | 1.E+05 | 2.E+04 | 7.88 | 0.07 | 1.034 | 5.E+05 | 1.E+05 | 7.95 | 0.07 | 0.923 | 1.E+05 | 2.E+04 | 7.76 | 0.08 | 1.209 | 8.E+05 | 1.E+05 | 8.52 | 0.07 | 1.48 | 1.E+05 | 3.E+04 | 8.21 | 0.07 | 1.158 | 6.E+05 | 1.E+05 |
| 74 | 11-keto-A4 | 5.19 | 0.06 | 1.083 | 9.E+05 | 2.E+05 | 4.80 | 0.06 | 1.047 | 8.E+06 | 2.E+06 | 4.93 | 0.06 | 1.169 | 1.E+06 | 3.E+05 | 4.71 | 0.07 | 1.103 | 6.E+06 | 1.E+06 | 5.25 | 0.07 | 1.204 | 9.E+05 | 2.E+05 | 4.85 | 0.07 | 1.282 | 6.E+06 | 1.E+06 |
| 75 | 2-ME1 | ----- | ----- | ----- | ----- | ----- | ----- | ----- | ----- | ----- | ----- | 6.63 | 0.07 | 1.098 | 6.E+04 | 1.E+04 | 6.43 | 0.07 | 1.288 | 3.E+04 | 7.E+03 | 7.11 | 0.08 | 1.246 | 6.E+04 | 1.E+04 | 6.78 | 0.06 | 1.128 | 3.E+04 | 7.E+03 |
| 76 | 16α-OHA4 | 4.82 | 0.07 | 1.077 | 2.E+06 | 6.E+05 | 4.30 | 0.06 | 1.054 | 3.E+07 | 7.E+06 | 4.52 | 0.07 | 1.109 | 4.E+06 | 9.E+05 | 4.22 | 0.07 | 1.091 | 2.E+07 | 4.E+06 | 4.91 | 0.07 | 1.276 | 3.E+06 | 6.E+05 | 4.37 | 0.07 | 1.43 | 2.E+07 | 4.E+06 |
| 77 | 11β-OHA4 | 5.38 | 0.07 | 1.05 | 4.E+06 | 9.E+05 | 4.91 | 0.07 | 1.058 | 8.E+06 | 2.E+06 | 5.08 | 0.07 | 1.111 | 5.E+06 | 1.E+06 | 4.82 | 0.07 | 1.077 | 1.E+07 | 3.E+06 | 5.49 | 0.08 | 1.278 | 5.E+06 | 9.E+05 | 5.04 | 0.07 | 1.324 | 1.E+07 | 2.E+06 |
| 78 | 7α-OHA4 | 4.32 | 0.06 | 1.067 | 3.E+06 | 8.E+05 | 3.75 | 0.07 | 1.04 | 2.E+07 | 4.E+06 | 4.06 | 0.07 | 1.076 | 3.E+06 | 7.E+05 | 3.73 | 0.07 | 1.076 | 3.E+07 | 7.E+06 | 4.41 | 0.07 | 1.249 | 4.E+06 | 8.E+05 | 3.81 | 0.07 | 1.478 | 2.E+07 | 4.E+06 |
| 79 | 19-OHA4 | 4.21 | 0.07 | 1.052 | 3.E+06 | 7.E+05 | 3.66 | 0.06 | 1.05 | 1.E+07 | 3.E+06 | 3.92 | 0.08 | 1.075 | 3.E+06 | 7.E+05 | 3.61 | 0.07 | 1.071 | 9.E+06 | 2.E+06 | 4.27 | 0.08 | 1.28 | 3.E+06 | 6.E+05 | 3.69 | 0.07 | 1.527 | 1.E+07 | 2.E+06 |
| 80 | 17α-MeT | 7.10 | 0.07 | 1.079 | 1.E+07 | 3.E+06 | 6.70 | 0.07 | 1.065 | 1.E+07 | 3.E+06 | 6.70 | 0.08 | 1.155 | 2.E+07 | 3.E+06 | 6.47 | 0.08 | 1.119 | 3.E+07 | 6.E+06 | 7.33 | 0.09 | 1.259 | 5.E+07 | 8.E+06 | 6.96 | 0.08 | 1.22 | 3.E+07 | 6.E+06 |
| 81 | 7α-OHDHEA | 4.21 | 0.07 | 1.132 | 2.E+06 | 4.E+05 | 3.52 | 0.07 | 1.081 | 5.E+06 | 1.E+06 | 3.91 | 0.07 | 1.071 | 2.E+06 | 5.E+05 | 3.51 | 0.07 | 1.077 | 6.E+06 | 1.E+06 | 4.32 | 0.08 | 1.219 | 6.E+06 | 1.E+06 | 3.57 | 0.08 | 1.482 | 6.E+06 | 1.E+06 |
| 82 | 19-OHT | 3.66 | 0.08 | 1.129 | 8.E+06 | 2.E+06 | 3.07 | 0.07 | 1.071 | 1.E+07 | 3.E+06 | 3.34 | 0.08 | 1.087 | 1.E+07 | 2.E+06 | 3.01 | 0.07 | 1.09 | 2.E+07 | 4.E+06 | 3.73 | 0.09 | 1.251 | 1.E+07 | 2.E+06 | 3.07 | 0.08 | 1.777 | 2.E+07 | 3.E+06 |
| 83 | 11β-OHT | 4.93 | 0.08 | 1.082 | 9.E+06 | 2.E+06 | 4.29 | 0.07 | 1.046 | 1.E+07 | 2.E+06 | 4.56 | 0.08 | 1.092 | 1.E+07 | 2.E+06 | 4.20 | 0.08 | 1.11 | 2.E+07 | 3.E+06 | 5.10 | 0.09 | 1.254 | 2.E+07 | 3.E+06 | 4.45 | 0.09 | 1.46 | 2.E+07 | 3.E+06 |
| 84 | 7α-OHT | 3.45 | 0.07 | 1.055 | 5.E+06 | 1.E+06 | 2.87 | 0.07 | 1.078 | 2.E+07 | 4.E+06 | 3.18 | 0.07 | 1.093 | 5.E+06 | 1.E+06 | 2.85 | 0.07 | 1.068 | 1.E+07 | 3.E+06 | 3.49 | 0.08 | 1.218 | 5.E+06 | 1.E+06 | 2.84 | 0.08 | 1.58 | 1.E+07 | 3.E+06 |
| 85 | Prog | 8.18 | 0.07 | 1.056 | 2.E+07 | 5.E+06 | 7.89 | 0.07 | 1.054 | 2.E+07 | 4.E+06 | 8.33 | 0.10 | 1.301 | 5.E+07 | 7.E+06 | 8.11 | 0.12 | 1.47 | 1.E+08 | 2.E+07 | 8.43 | 0.08 | 1.249 | 6.E+07 | 1.E+07 | 8.13 | 0.07 | 1.2 | 6.E+07 | 1.E+07 |
| 86 | 5β-DiHProg | 10.98 | 0.10 | 1.103 | 1.E+06 | 2.E+05 | ----- | ----- | ----- | ----- | ----- | 10.83 | 0.09 | 1.147 | 2.E+06 | 4.E+05 | 10.71 | 0.10 | 1.312 | 3.E+06 | 4.E+05 | 11.57 | 0.10 | 1.19 | 1.E+06 | 2.E+05 | 11.37 | 0.08 | 1.205 | 3.E+06 | 4.E+05 |
| 87 | 5α-DiHProg | 9.61 | 0.15 | 1.289 | 4.E+04 | 3.E+03 | 9.38 | 0.08 | 0.952 | 2.E+04 | 5.E+03 | 9.46 | 0.09 | 0.707 | 1.E+04 | 3.E+03 | 9.30 | 0.13 | 1.035 | 2.E+05 | 2.E+04 | 10.00 | 0.09 | 1.011 | 1.E+05 | 2.E+04 | 9.69 | 0.12 | 1.089 | 7.E+04 | 1.E+04 |
| 88 | Preg | 11.56 | 0.09 | 1.361 | 4.E+05 | 7.E+04 | 11.34 | 0.07 | 1.171 | 1.E+05 | 3.E+04 | 11.51 | 0.10 | 1.11 | 8.E+05 | 1.E+05 | 11.39 | 0.13 | 1.336 | 2.E+06 | 3.E+05 | 11.94 | 0.10 | 1.369 | 7.E+05 | 1.E+05 | 11.73 | 0.10 | 1.687 | 1.E+06 | 1.E+05 |
| 89 | 20α-OHProg | 7.83 | 0.07 | 1.065 | 2.E+07 | 4.E+06 | 7.50 | 0.07 | 1.039 | 1.E+07 | 3.E+06 | 7.42 | 0.08 | 1.099 | 2.E+07 | 4.E+06 | 7.23 | 0.08 | 1.108 | 4.E+07 | 8.E+06 | 8.10 | 0.08 | 1.209 | 4.E+07 | 8.E+06 | 7.77 | 0.07 | 1.178 | 4.E+07 | 8.E+06 |
| 90 | 11β-OHProg | 6.73 | 0.07 | 1.084 | 2.E+07 | 4.E+06 | 6.36 | 0.07 | 1.056 | 3.E+07 | 8.E+06 | 6.39 | 0.08 | 1.153 | 2.E+07 | 5.E+06 | 6.17 | 0.07 | 1.117 | 4.E+07 | 8.E+06 | 6.86 | 0.08 | 1.262 | 2.E+07 | 4.E+06 | 6.50 | 0.08 | 1.22 | 4.E+07 | 7.E+06 |
| 91 | 17α-OHProg | 6.95 | 0.07 | 1.066 | 1.E+07 | 3.E+06 | 6.60 | 0.07 | 1.066 | 1.E+07 | 2.E+06 | 6.64 | 0.07 | 1.128 | 1.E+07 | 2.E+06 | 6.43 | 0.07 | 1.12 | 2.E+07 | 5.E+06 | 7.09 | 0.07 | 1.302 | 1.E+07 | 2.E+06 | 6.75 | 0.07 | 1.19 | 2.E+07 | 5.E+06 |
| 92 | DOC | 6.51 | 0.07 | 1.075 | 1.E+07 | 2.E+06 | 6.14 | 0.06 | 1.062 | 2.E+07 | 5.E+06 | 6.17 | 0.08 | 1.113 | 1.E+07 | 2.E+06 | 5.96 | 0.07 | 1.083 | 5.E+07 | 1.E+07 | 6.64 | 0.08 | 1.295 | 1.E+07 | 2.E+06 | 6.28 | 0.07 | 1.259 | 4.E+07 | 8.E+06 |
| 93 | 21-OHPreg | 6.66 | 0.07 | 1.08 | 1.E+05 | 3.E+04 | 6.25 | 0.07 | 1.151 | 1.E+06 | 2.E+05 | 6.30 | 0.07 | 1.177 | 2.E+05 | 5.E+04 | 6.06 | 0.07 | 1.368 | 5.E+05 | 9.E+04 | 6.92 | 0.07 | 1.094 | 1.E+05 | 3.E+04 | 6.52 | 0.08 | 1.1 | 4.E+05 | 9.E+04 |
| 94 | 17,20α-(OH)2Prog | 8.66 | 0.08 | 0.987 | 5.E+05 | 8.E+04 | 8.46 | 0.07 | 1.031 | 3.E+06 | 6.E+05 | 8.43 | 0.09 | 1.163 | 6.E+05 | 1.E+05 | 8.32 | 0.08 | 1.003 | 2.E+06 | 3.E+05 | 8.59 | 0.08 | 1.982 | 5.E+05 | 9.E+04 | 8.40 | 0.08 | 1.35 | 2.E+06 | 4.E+05 |
| 95 | 5α-DiHDOC | 9.46 | 0.09 | 1.014 | 5.E+05 | 1.E+05 | 9.21 | 0.07 | 1.122 | 2.E+05 | 5.E+04 | 9.19 | 0.09 | 1.197 | 6.E+05 | 1.E+05 | 9.07 | 0.09 | 1.102 | 1.E+06 | 2.E+05 | 9.95 | 0.08 | 1.258 | 7.E+05 | 1.E+05 | 9.74 | 0.08 | 0.985 | 7.E+05 | 1.E+05 |
| 96 | 7α-OHPreg | 5.67 | 0.07 | 1.036 | 2.E+06 | 5.E+05 | 5.09 | 0.07 | 1.078 | 1.E+07 | 2.E+06 | 5.36 | 0.08 | 1.175 | 2.E+06 | 5.E+05 | 5.03 | 0.08 | 1.105 | 6.E+06 | 1.E+06 | 5.81 | 0.08 | 1.282 | 2.E+06 | 4.E+05 | 5.25 | 0.08 | 1.334 | 6.E+06 | 1.E+06 |
| 97 | 17α-OHPreg | 6.95 | 0.07 | 0.889 | 7.E+04 | 2.E+04 | 6.54 | 0.07 | 1.026 | 4.E+05 | 9.E+04 | 6.63 | 0.09 | 1.15 | 4.E+04 | 1.E+04 | 6.37 | 0.08 | 1.127 | 4.E+05 | 8.E+04 | 7.17 | 0.07 | 1.014 | 2.E+05 | 5.E+04 | 6.79 | 0.08 | 1.304 | 4.E+05 | 8.E+04 |
| 98 | 11-DHB | 4.84 | 0.07 | 1.065 | 7.E+05 | 2.E+05 | 4.34 | 0.06 | 1.044 | 5.E+06 | 1.E+06 | 4.56 | 0.07 | 1.118 | 1.E+06 | 2.E+05 | 4.28 | 0.07 | 1.029 | 4.E+06 | 8.E+05 | 4.90 | 0.07 | 1.356 | 8.E+05 | 2.E+05 | 4.39 | 0.07 | 1.371 | 4.E+06 | 7.E+05 |
| 99 | B | 5.32 | 0.07 | 1.082 | 4.E+06 | 9.E+05 | 4.79 | 0.07 | 1.04 | 1.E+07 | 2.E+06 | 4.99 | 0.08 | 1.089 | 6.E+06 | 1.E+06 | 4.69 | 0.07 | 1.107 | 8.E+06 | 2.E+06 | 5.45 | 0.08 | 1.294 | 5.E+06 | 9.E+05 | 4.92 | 0.08 | 1.412 | 1.E+07 | 2.E+06 |
| 100 | 11-DOF | 5.52 | 0.07 | 1.045 | 3.E+06 | 6.E+05 | 5.00 | 0.07 | 1.045 | 5.E+06 | 1.E+06 | 5.19 | 0.07 | 1.077 | 3.E+06 | 7.E+05 | 4.90 | 0.07 | 1.135 | 9.E+06 | 2.E+06 | 5.61 | 0.07 | 1.32 | 3.E+06 | 6.E+05 | 5.12 | 0.07 | 1.316 | 9.E+06 | 2.E+06 |
| 101 | 21-DOF | 5.34 | 0.07 | 1.076 | 7.E+06 | 2.E+06 | 4.84 | 0.07 | 1.056 | 1.E+07 | 2.E+06 | 5.04 | 0.07 | 1.07 | 7.E+06 | 1.E+06 | 4.76 | 0.07 | 1.083 | 2.E+07 | 5.E+06 | 5.41 | 0.08 | 1.285 | 8.E+06 | 2.E+06 | 4.92 | 0.08 | 1.334 | 1.E+07 | 3.E+06 |
| 102 | 17,21-(OH)2Preg | ----- | ----- | ----- | ----- | ----- | 1.94 | 0.05 | 1.673 | 5.E+04 | 1.E+04 | ----- | ----- | ----- | ----- | ----- | 1.89 | 0.06 | 2.017 | 4.E+04 | 7.E+03 | ----- | ----- | ----- | ----- | ----- | 1.83 | 0.10 | 2.13 | 6.E+04 | 8.E+03 |
| 103 | THA | 5.83 | 0.07 | 1.045 | 3.E+06 | 7.E+05 | 5.27 | 0.07 | 1.042 | 9.E+06 | 2.E+06 | 5.57 | 0.07 | 1.132 | 3.E+06 | 7.E+05 | 5.25 | 0.08 | 1.121 | 1.E+07 | 2.E+06 | 5.98 | 0.07 | 1.236 | 3.E+06 | 7.E+05 | 5.44 | 0.08 | 1.322 | 1.E+07 | 2.E+06 |
| 104 | 5β-DiHB | 5.34 | 0.07 | 0.975 | 5.E+04 | 1.E+04 | 5.75 | 0.23 | 10.489 | 4.E+06 | 2.E+05 | ----- | ----- | ----- | ----- | ----- | 5.66 | 0.24 | 10.251 | 8.E+06 | 3.E+05 | ----- | ----- | ----- | ----- | ----- | 5.92 | 0.15 | 9.916 | 6.E+06 | 3.E+05 |
| 105 | E1-S | 4.06 | 0.06 | 1.079 | 1.E+07 | 3.E+06 | 3.32 | 0.07 | 1.256 | 6.E+07 | 1.E+07 | 3.74 | 0.07 | 1.082 | 1.E+07 | 3.E+06 | 2.91 | 0.09 | 1.388 | 5.E+07 | 7.E+06 | 3.94 | 0.07 | 1.189 | 1.E+07 | 2.E+06 | 4.43 | 0.12 | 1.303 | 6.E+07 | 8.E+06 |
| 106 | THB | 5.84 | 0.07 | 1.046 | 5.E+05 | 1.E+05 | 5.27 | 0.07 | 1.099 | 2.E+06 | 4.E+05 | 5.56 | 0.07 | 1.084 | 6.E+05 | 1.E+05 | 5.23 | 0.08 | 1.149 | 2.E+06 | 4.E+05 | 6.00 | 0.08 | 1.301 | 6.E+05 | 1.E+05 | 5.47 | 0.08 | 1.225 | 3.E+06 | 5.E+05 |
| 107 | A | 3.39 | 0.07 | 1.169 | 5.E+05 | 1.E+05 | 2.81 | 0.14 | 0.97 | 1.E+06 | 1.E+05 | 3.10 | 0.09 | 1.084 | 7.E+05 | 1.E+05 | 2.77 | 0.13 | 1.133 | 2.E+06 | 2.E+05 | 3.40 | 0.09 | 1.27 | 7.E+05 | 1.E+05 | 2.74 | 0.15 | 1.327 | 2.E+06 | 2.E+05 |
| 108 | E | 4.13 | 0.07 | 1.072 | 1.E+06 | 3.E+05 | 3.53 | 0.07 | 1.081 | 5.E+06 | 1.E+06 | 3.85 | 0.07 | 1.094 | 1.E+06 | 3.E+05 | 3.50 | 0.07 | 1.077 | 9.E+06 | 2.E+06 | 4.16 | 0.08 | 1.289 | 1.E+06 | 3.E+05 | 3.52 | 0.07 | 1.496 | 7.E+06 | 1.E+06 |
| 109 | 5β-DiHE | 5.27 | 0.06 | 1.016 | 8.E+05 | 2.E+05 | 4.73 | 0.12 | 13.942 | 2.E+07 | 9.E+05 | 5.02 | 0.08 | 1.026 | 1.E+06 | 2.E+05 | 4.71 | 0.15 | 13.098 | 3.E+07 | 1.E+06 | 5.38 | 0.07 | 1.211 | 9.E+05 | 2.E+05 | 4.86 | 0.14 | 13.155 | 3.E+07 | 1.E+06 |
| 110 | 18-OHB | 3.14 | 0.08 | 1.703 | 2.E+06 | 3.E+05 | 2.59 | 0.09 | 3.458 | 3.E+06 | 4.E+05 | 2.84 | 0.09 | 1.403 | 2.E+06 | 4.E+05 | 2.56 | 0.09 | 3.395 | 4.E+06 | 5.E+05 | 3.15 | 0.11 | 1.388 | 2.E+06 | 2.E+05 | 2.52 | 0.10 | 4.841 | 5.E+06 | 4.E+05 |
| 111 | F | 4.10 | 0.07 | 1.083 | 4.E+06 | 8.E+05 | 3.45 | 0.07 | 1.058 | 7.E+06 | 2.E+06 | 3.80 | 0.07 | 1.059 | 4.E+06 | 9.E+05 | 3.42 | 0.07 | 1.091 | 1.E+07 | 3.E+06 | 4.18 | 0.08 | 1.197 | 5.E+06 | 8.E+05 | 3.45 | 0.08 | 1.543 | 1.E+07 | 2.E+06 |
| 112 | 11β,17α,21-(OH)3Preg | 3.46 | 0.06 | 1.033 | 7.E+04 | 2.E+04 | 2.84 | 0.07 | 1.079 | 4.E+05 | 1.E+05 | 3.17 | 0.07 | 1.081 | 9.E+04 | 2.E+04 | 2.82 | 0.07 | 1.085 | 4.E+05 | 1.E+05 | 3.46 | 0.07 | 1.118 | 9.E+04 | 2.E+04 | 2.79 | 0.07 | 1.476 | 5.E+05 | 1.E+05 |
| 113 | 5β-DiHF | 5.22 | 0.07 | 0.987 | 3.E+05 | 7.E+04 | 4.63 | 0.12 | 10.091 | 2.E+07 | 8.E+05 | 4.95 | 0.07 | 1.022 | 4.E+05 | 8.E+04 | 4.62 | 0.14 | 11.976 | 2.E+07 | 7.E+05 | 5.37 | 0.07 | 1.027 | 3.E+05 | 7.E+04 | 4.79 | 0.14 | 10.584 | 2.E+07 | 8.E+05 |
| 114 | DHEA-S | 4.49 | 0.06 | 1.09 | 2.E+06 | 4.E+05 | 3.60 | 0.07 | 1.221 | 2.E+07 | 3.E+06 | 4.14 | 0.07 | 1.095 | 2.E+06 | 5.E+05 | 3.21 | 0.09 | 1.317 | 1.E+07 | 2.E+06 | 4.44 | 0.07 | 1.246 | 2.E+06 | 4.E+05 | 4.91 | 0.13 | 1.252 | 2.E+07 | 2.E+06 |
| 115 | 6β-OHF | 13.22 | 0.11 | 1.152 | 2.E+06 | 3.E+05 | 13.07 | 0.09 | 1.096 | 1.E+06 | 2.E+05 | 13.11 | 0.11 | 1.277 | 2.E+06 | 2.E+05 | 13.01 | 0.12 | 1.365 | 4.E+06 | 6.E+05 | 13.88 | 0.10 | 1.279 | 3.E+06 | 4.E+05 | 13.74 | 0.11 | 1.445 | 1.E+07 | 1.E+06 |
| 116 | 18-OHF | 13.21 | 0.10 | 1.142 | 3.E+06 | 4.E+05 | 1.25 | 0.21 | 1.506 | 6.E+05 | 5.E+04 | 13.11 | 0.11 | 1.402 | 1.E+06 | 1.E+05 | 13.01 | 0.12 | 1.276 | 4.E+06 | 5.E+05 | 13.88 | 0.11 | 1.169 | 4.E+06 | 5.E+05 | 13.73 | 0.10 | 1.452 | 8.E+06 | 1.E+06 |
| 117 | Chol | 11.84 | 0.11 | 1.029 | 1.E+06 | 2.E+05 | 11.34 | 0.08 | 0.916 | 4.E+05 | 8.E+04 | 11.63 | 0.10 | 1.041 | 1.E+06 | 2.E+05 | 10.70 | 0.12 | 1.264 | 4.E+06 | 5.E+05 | 12.40 | 0.10 | 1.052 | 2.E+06 | 3.E+05 | 13.41 | 0.17 | 1.113 | 5.E+06 | 5.E+05 |
| 118 | Preg-S | 5.88 | 0.07 | 1.077 | 3.E+06 | 7.E+05 | 5.21 | 0.08 | 1.246 | 3.E+07 | 6.E+06 | 5.51 | 0.07 | 1.105 | 3.E+06 | 7.E+05 | 4.78 | 0.08 | 1.322 | 2.E+07 | 4.E+06 | 5.90 | 0.07 | 1.293 | 7.E+06 | 1.E+06 | 6.45 | 0.11 | 1.201 | 3.E+07 | 4.E+06 |
| 119 | 20α-OHChol | 11.72 | 0.10 | 1.101 | 2.E+06 | 3.E+05 | 11.54 | 0.09 | 1.159 | 6.E+05 | 1.E+05 | 11.49 | 0.11 | 1.215 | 2.E+06 | 2.E+05 | 11.38 | 0.13 | 1.236 | 3.E+06 | 4.E+05 | 12.30 | 0.10 | 1.31 | 2.E+06 | 3.E+05 | 12.13 | 0.10 | 1.251 | 4.E+06 | 6.E+05 |
| 120 | 22-OHChol | 11.62 | 0.10 | 0.967 | 4.E+05 | 5.E+04 | 11.44 | 0.09 | 1.077 | 2.E+06 | 3.E+05 | 11.39 | 0.09 | 1.155 | 4.E+05 | 7.E+04 | 11.28 | 0.09 | 1.206 | 1.E+06 | 3.E+05 | ----- | ----- | ----- | ----- | ----- | 11.91 | 0.09 | 1.137 | 2.E+06 | 3.E+05 |
| 121 | E1-G | 2.14 | 0.07 | 1.103 | 7.E+05 | 2.E+05 | 2.88 | 0.08 | 1.376 | 5.E+06 | 1.E+06 | 1.90 | 0.07 | 1.193 | 9.E+05 | 2.E+05 | 2.94 | 0.31 | 4.791 | 5.E+06 | 2.E+05 | 1.93 | 0.07 | 1.229 | 9.E+05 | 2.E+05 | 2.96 | 0.11 | 1.988 | 6.E+06 | 7.E+05 |
| 122 | E2-3-G | 1.67 | 0.06 | 1.089 | 1.E+06 | 3.E+05 | 2.31 | 0.07 | 1.495 | 7.E+06 | 1.E+06 | 1.50 | 0.06 | 1.2 | 1.E+06 | 3.E+05 | 2.35 | 0.25 | 4.589 | 6.E+06 | 3.E+05 | 1.49 | 0.06 | 1.362 | 1.E+06 | 3.E+05 | 2.30 | 0.10 | 2.115 | 8.E+06 | 1.E+06 |
| 123 | E3-16-G | 1.52 | 0.05 | 1.516 | 7.E+03 | 2.E+03 | 1.63 | 0.07 | 1.415 | 9.E+04 | 2.E+04 | 1.44 | 0.05 | 1.079 | 1.E+04 | 4.E+03 | 1.66 | 0.06 | 3.406 | 4.E+04 | 2.E+03 | 1.38 | 0.05 | 1.18 | 1.E+04 | 4.E+03 | 1.68 | 0.11 | 1.283 | 8.E+04 | 9.E+03 |
| 124 | T-G | 2.13 | 0.07 | 1.184 | 4.E+06 | 8.E+05 | 3.03 | 0.08 | 1.371 | 1.E+07 | 2.E+06 | 1.87 | 0.07 | 1.267 | 5.E+06 | 1.E+06 | 3.08 | 0.30 | 4.375 | 1.E+07 | 5.E+05 | 1.92 | 0.08 | 1.301 | 4.E+06 | 7.E+05 | 3.10 | 0.11 | 1.897 | 1.E+07 | 1.E+06 |
| 125 | Et-G | 3.39 | 0.07 | 1.102 | 3.E+05 | 6.E+04 | 5.14 | 0.09 | 1.251 | 2.E+06 | 4.E+05 | 3.04 | 0.08 | 1.144 | 3.E+05 | 5.E+04 | 5.17 | 0.26 | 4.322 | 2.E+06 | 1.E+05 | 3.26 | 0.08 | 1.169 | 3.E+05 | 5.E+04 | 5.46 | 0.10 | 1.713 | 3.E+06 | 3.E+05 |
| 126 | An-G | 3.44 | 0.07 | 1.096 | 3.E+05 | 7.E+04 | 5.36 | 0.09 | 1.398 | 3.E+06 | 4.E+05 | 3.10 | 0.08 | 1.131 | 4.E+05 | 7.E+04 | 5.39 | 0.22 | 3.476 | 2.E+06 | 1.E+05 | 3.32 | 0.08 | 1.134 | 8.E+05 | 1.E+05 | 5.65 | 0.10 | 1.711 | 3.E+06 | 4.E+05 |
| 127 | Chol-S | 11.85 | 0.11 | 0.972 | 5.E+06 | 8.E+05 | 11.34 | 0.09 | 1.092 | 4.E+07 | 8.E+06 | 11.62 | 0.10 | 1.089 | 5.E+06 | 8.E+05 | 10.70 | 0.11 | 1.233 | 4.E+07 | 5.E+06 | 12.40 | 0.11 | 1.063 | 7.E+06 | 1.E+06 | 13.43 | 0.19 | 1.04 | 6.E+07 | 5.E+06 |
| 128 | E2-3-G-17-S | 0.94 | 0.06 | 1.259 | 1.E+06 | 3.E+05 | 1.46 | 0.05 | 2.115 | 4.E+05 | 9.E+04 | 0.90 | 0.06 | 1.479 | 2.E+06 | 4.E+05 | ----- | ----- | ----- | ----- | ----- | 0.84 | 0.07 | 1.391 | 2.E+06 | 4.E+05 | 1.71 | 0.48 | 17.271 | 3.E+06 | 1.E+04 |

^a^The peak width was defined as 50% peak height (W_0.5_).

^b^Symmetry factors (S) were calculated by vendor software, LabSolutions Version 5.99 SP2.

^c^Intensity threshold has been set to 1500.

**Supplemental Table S8.** Compounds contributing to the separation of PCA and their typical chemical properties.

|  | Compound name | Loadings 1 | Loadings 2 | log *P_ow_* | p*K*_a_ | p*K*_b_ |
| --- | --- | --- | --- | --- | --- | --- |
| 1 | E2-3-G-17-S | 0.40038 | -0.72584 | 1.331 | -1.38 | -3.69 |
| 2 | PGK2 | 0.13146 | 0.033767 | 2.876 | 4.35 | -1.6 |
| 3 | 5β-DiHB | 0.10517 | 0.069361 | 2.343 | 13.86 | -0.29 |
| 4 | α-MCA | 0.099605 | 0.02995 | 3.273 | 4.6 | -2.69 |
| 5 | ω-MCA | 0.099355 | 0.029599 | 3.273 | 4.6 | -2.69 |
| 6 | β-MCA | 0.098672 | 0.032957 | 3.273 | 4.6 | -2.69 |
| 7 | 7-keto-DCA | 0.089754 | 0.02919 | 3.178 | 4.44 | -0.33 |
| 8 | 22-OHChol | 0.087327 | 0.010729 | 6.026 | 18.2 | -0.73 |
| 9 | PGF2α | 0.084715 | 0.023466 | 2.736 | 4.36 | -1.63 |
| 10 | PGE2 | 0.083796 | 0.024568 | 2.801 | 4.3 | -1.63 |
| 11 | PGH2 | 0.083687 | 0.024649 | 3.917 | 4.36 | -1.63 |
| 12 | PGE1 | 0.08269 | 0.026357 | 3.054 | 4.35 | -1.63 |
| 13 | PGD2 | 0.079032 | 0.025852 | 2.801 | 4.4 | -1.6 |
| 14 | TXB2 | 0.076197 | 0.019896 | 2.537 | 4.27 | -2.85 |
| 15 | RvD2 | 0.070463 | 0.023199 | 3.143 | 4.64 | -1.61 |
| 16 | PGG2 | 0.070408 | 0.024911 | 4.235 | 4.36 | -4.24 |
| 17 | CA | 0.069218 | 0.030349 | 3.205 | 4.48 | -0.16 |
| 18 | LXB4 | 0.06813 | 0.022105 | 2.932 | 4.65 | -1.55 |
| 19 | An-G | 0.065336 | 0.021339 | 1.764 | 3.47 | -3.69 |
| 20 | GHDCA | 0.064866 | 0.022378 | 3.358 | 3.77 | -0.66 |
| 21 | Et-G | 0.0607 | 0.020542 | 1.764 | 3.47 | -3.69 |
| 22 | UDCA | 0.060179 | 0.028839 | 4.178 | 4.6 | -0.54 |
| 23 | GUDCA | 0.059965 | 0.020591 | 3.358 | 3.77 | -0.29 |
| 24 | HDCA | 0.059611 | 0.029079 | 4.178 | 4.79 | -2.69 |
| 25 | RvD3 | 0.059335 | 0.019143 | 3.098 | 4.61 | -1.29 |
| 26 | PGA2 | 0.059053 | 0.024469 | 3.64 | 4.4 | -1.6 |
| 27 | GCA | 0.056202 | 0.018875 | 2.395 | 3.77 | -0.04 |
| 28 | PGB2 | 0.055321 | 0.023839 | 3.796 | 4.25 | -1.59 |
| 29 | RvD1 | 0.05263 | 0.019783 | 3.143 | 4.47 | -1.61 |
| 30 | 12-keto-LCA | 0.051952 | 0.026515 | 4.221 | 4.56 | -1.36 |
| 31 | RvE1 | 0.051119 | 0.012713 | 2.636 | 4.65 | -1.11 |
| 32 | 7-keto-LCA | 0.050927 | 0.026098 | 4.151 | 4.56 | -1.33 |
| 33 | 15-epi-LXA4 | 0.049997 | 0.01887 | 2.932 | 4.48 | -1.58 |
| 34 | LXA4 | 0.048806 | 0.018434 | 2.932 | 4.48 | -1.58 |
| 35 | Allo-LCA | 0.048253 | 0.048295 | 5.18 | 4.79 | -1.36 |
| 36 | Protectin D1 | 0.047793 | 0.02032 | 4.071 | 4.82 | -1.31 |
| 37 | 17,21-(OH)2Preg | 0.046357 | 0.013005 | 2.465 | 12.59 | -1.4 |
| 38 | Maresin-1 | 0.045099 | 0.019074 | 4.071 | 4.64 | -1.31 |
| 39 | GCDCA | 0.042853 | 0.019579 | 3.358 | 3.77 | -0.29 |
| 40 | GDCA | 0.040989 | 0.019668 | 3.381 | 3.77 | -0.18 |
| 41 | Allo-CDCA | 0.040224 | 0.02355 | 4.178 | 4.6 | -0.54 |
| 42 | CDCA | 0.039714 | 0.023426 | 4.178 | 4.6 | -0.54 |
| 43 | GLCA | 0.039573 | 0.022433 | 4.348 | 3.77 | -0.58 |
| 44 | RvD4 | 0.039551 | 0.01596 | 3.143 | 4.45 | -1.61 |
| 45 | LTB4 | 0.039439 | 0.01787 | 3.86 | 4.65 | -1.27 |
| 46 | LTD4 | 0.038745 | -0.00090385 | 3.114 | 3.29 | 8.05 |
| 47 | DCA | 0.038665 | 0.023141 | 4.201 | 4.65 | -0.35 |
| 48 | 17-HDoHE | 0.036253 | 0.018549 | 5.051 | 4.89 | -1.61 |
| 49 | T-G | 0.033503 | 0.010063 | 1.667 | 3.63 | -3.69 |
| 50 | 12-HETE | 0.03292 | 0.018053 | 4.839 | 4.89 | -1.61 |
| 51 | 5-HETE | 0.031829 | 0.017136 | 5.35 | 0 | -7.05 |
| 52 | 15-HpETE | 0.031644 | 0.01756 | 5.145 | 4.82 | -4.24 |
| 53 | 5-HpETE | 0.030853 | 0.017009 | 5.145 | 4.39 | -4.24 |
| 54 | 12-HpETE | 0.030428 | 0.01683 | 5.145 | 4.89 | -4.24 |
| 55 | DHA | 0.029194 | 0.015237 | 6.04 | 4.89 | 0 |
| 56 | E1-G | 0.028746 | 0.0082537 | 1.585 | 3.3 | -3.69 |
| 57 | EPA | 0.028186 | 0.014423 | 5.578 | 4.82 | 0 |
| 58 | LCA | 0.028028 | 0.02226 | 5.18 | 4.79 | -1.36 |
| 59 | AA | 0.025256 | 0.013885 | 5.828 | 4.82 | 0 |
| 60 | E2-3-G | 0.023622 | 0.0062445 | 1.557 | 3.3 | -0.88 |
| 61 | E3-16-G | 0.0062801 | 0.0016638 | 0.8148 | 3.46 | -3.35 |
| 62 | Prog | 0.002637 | -0.15052 | 4.118 | 18.92 | -4.82 |
| 63 | 17,20α-(OH)2Prog | -0.005164 | 0.0054657 | 3.323 | 13.42 | -3.13 |
| 64 | DHT | -0.0065194 | -0.27784 | 3.641 | 19.38 | -0.88 |
| 65 | 6β-OHF | -0.006851 | 0.0074888 | 0.5712 | 12.58 | -2.85 |
| 66 | Preg | -0.006945 | 0.00075079 | 4.049 | 18.2 | -1.4 |
| 67 | 20α-OHChol | -0.0075809 | 0.011283 | 6.058 | 18.2 | -0.26 |
| 68 | ED | -0.0083848 | 0.0065617 | 3.662 | 19.78 | -7.11 |
| 69 | 5α-DiHDOC | -0.0083938 | 0.010269 | 3.346 | 13.86 | -3.3 |
| 70 | 5β-DHT | -0.008609 | 0.0090119 | 3.641 | 19.38 | -0.88 |
| 71 | AD | -0.0091013 | 0.008819 | 3.662 | 19.78 | -7.11 |
| 72 | A4 | -0.0096148 | 0.0078939 | 3.66 | 19.03 | -4.82 |
| 73 | An | -0.0098577 | 0.0073973 | 3.737 | 18.3 | -1.36 |
| 74 | 17α-OHProg | -0.01002 | 0.0081083 | 3.299 | 12.7 | -3.8 |
| 75 | 11-keto-A4 | -0.010279 | 0.0037796 | 2.627 | 17.99 | -4.87 |
| 76 | 11β-OHProg | -0.01041 | 0.0087029 | 3.106 | 18.88 | -0.26 |
| 77 | 20α-OHProg | -0.010429 | 0.013259 | 4.189 | 19 | -1.05 |
| 78 | DOC | -0.010432 | 0.0083818 | 3.344 | 13.86 | -3.3 |
| 79 | 17α-MeT | -0.011557 | 0.011671 | 3.91 | 19.09 | -0.53 |
| 80 | 17α-OHPreg | -0.011901 | 0.0084549 | 3.23 | 12.7 | -1.4 |
| 81 | 21-OHPreg | -0.01196 | 0.0098068 | 3.275 | 13.86 | -1.4 |
| 82 | T | -0.012173 | 0.010336 | 3.636 | 19.09 | -0.88 |
| 83 | 11β-OHA4 | -0.0124 | 0.0044673 | 2.649 | 14.88 | -2.85 |
| 84 | 2-OHE1 | -0.012736 | 0.0035832 | 3.335 | 9.67 | -6.25 |
| 85 | 11-DHB | -0.012927 | 0.0025263 | 2.32 | 13.86 | -3.3 |
| 86 | 21-DOF | -0.013052 | 0.003443 | 2.297 | 12.69 | -2.85 |
| 87 | 11-DOF | -0.013554 | 0.0045508 | 2.534 | 12.59 | -3.33 |
| 88 | 16α-OHA4 | -0.013719 | 0.0030204 | 2.812 | 13.38 | -3.51 |
| 89 | TLCA | -0.013767 | 0.022466 | 3.917 | -0.84 | 0.1 |
| 90 | 2-ME1 | -0.013838 | -0.18389 | 3.597 | 10.29 | -4.88 |
| 91 | 18-OHB | -0.013842 | 0.00035478 | 1.373 | 13.8 | -2.77 |
| 92 | 5β-DiHE | -0.014073 | 0.0011758 | 1.517 | 12.58 | -3.33 |
| 93 | B | -0.014107 | 0.004429 | 2.342 | 13.86 | -0.26 |
| 94 | 19-OHA4 | -0.014315 | 0.0017207 | 2.675 | 18.71 | -0.97 |
| 95 | 16α-OHE1 | -0.014422 | 0.0017609 | 2.863 | 10.33 | -3.51 |
| 96 | TβMCA | -0.014542 | 0.0067002 | 2.039 | -0.98 | 0.08 |
| 97 | THA | -0.014604 | 0.0014905 | 2.391 | 13.86 | -1.36 |
| 98 | THB | -0.014855 | 0.002456 | 2.418 | 13.86 | -0.25 |
| 99 | 7α-OHA4 | -0.014953 | 0.0003449 | 2.649 | 17.14 | -0.61 |
| 100 | TαMCA | -0.015131 | 0.0061436 | 2.039 | -0.98 | 0.08 |
| 101 | 7α-OHT | -0.015164 | -0.00015979 | 2.625 | 17.14 | -0.44 |
| 102 | 7α-OHPreg | -0.015185 | 0.0031287 | 3.23 | 12.7 | -1.4 |
| 103 | A | -0.015277 | 0.00016571 | 1.497 | 13.82 | -2.85 |
| 104 | 5β-DiHF | -0.015339 | 0.00092247 | 1.541 | 12.58 | -2.84 |
| 105 | E | -0.015499 | 3.75E-05 | 1.517 | 12.58 | -3.33 |
| 106 | 19-OHT | -0.015539 | 0.0018 | 2.651 | 18.74 | -0.62 |
| 107 | 2-OHE2 | -0.01554 | 0.0027762 | 3.309 | 9.67 | -0.88 |
| 108 | 11β,17α,21-(OH)3Preg | -0.015887 | -0.00025667 | 1.47 | 12.58 | -1.4 |
| 109 | Preg-S | -0.016087 | 0.022663 | 3.737 | -1.36 | -7.36 |
| 110 | Chol-S | -0.016675 | 0.034718 | 6.704 | -1.36 | 0 |
| 111 | Chol | -0.016751 | 0.034387 | 7.052 | 18.2 | -1.4 |
| 112 | 11β-OHT | -0.017063 | 0.0041476 | 2.625 | 14.48 | -2.85 |
| 113 | F | -0.017199 | 0.00016967 | 1.539 | 12.58 | -2.85 |
| 114 | E1-S | -0.017863 | 0.019904 | 3.237 | -1.75 | -7.48 |
| 115 | 7α-OHDHEA | -0.018036 | -0.00045493 | 2.625 | 18.2 | -0.81 |
| 116 | TDCA | -0.019731 | 0.016677 | 2.954 | -0.94 | 0.22 |
| 117 | DHEA-S | -0.021271 | 0.020029 | 3.28 | -1.36 | -7.48 |
| 118 | TCDCA | -0.021513 | 0.015868 | 2.931 | -0.99 | 0.18 |
| 119 | 5α-DiHProg | -0.025815 | 0.0051735 | 4.12 | 19.34 | -7.05 |
| 120 | TUDCA | -0.027666 | 0.013737 | 2.931 | -0.99 | 0.18 |
| 121 | THDCA | -0.029999 | 0.012082 | 2.931 | -0.98 | 0.08 |
| 122 | TCA | -0.032795 | 0.01264 | 1.971 | -1.06 | 0.28 |
| 123 | E3 | -0.071836 | -0.0012643 | 2.742 | 10.33 | -3.16 |
| 124 | 5β-DiHProg | -0.092506 | -0.24324 | 4.12 | 19.34 | -7.05 |
| 125 | E1 | -0.11465 | 0.11181 | 3.712 | 10.33 | -5.45 |
| 126 | 18-OHF | -0.12266 | -0.33561 | 0.5755 | 12.51 | -2.8 |
| 127 | LTE4 | -0.20433 | -0.30062 | 3.167 | 2.39 | 9.13 |
| 128 | LTC4 | -0.72144 | -0.14263 | 1.65 | 1.8 | 9.31 |

**Supplemental Table S9.** MRM transitions and RTs of amino acids, LPCs, LPEs, PCs, and TGs in LC/MRM analysis.

| Compound name | Class | Formula | Exact mass | Precursor ion | Product ion | MRM transitions | Q1 Pre-bias (V) | Collision energy (V) | Q3 Pre-bias (V) | RT (min) |
| --- | --- | --- | --- | --- | --- | --- | --- | --- | --- | --- |
|  |  |  |  |  |  |  |  |  |  |  |
| Serine | Amino acid | C3H7NO3 | 105.0426 | [M + H]+ | - | 106.1 >60.151 | -40 | -13 | -40 | 1.04±0.07 |
| Glutamic acid | Amino acid | C5H9NO4 | 147.0532 | [M + H]+ | - | 148.1 >84.11 | -40 | -17 | -40 | 1.03±0.13 |
| Phenylalanine | Amino acid | C9H11NO2 | 165.0790 | [M + H]+ | - | 166.1 >120.11 | -40 | -15 | -40 | 1.51±0.01 |
| LPC 16:0 | LPC | C24H50N1O7P1 | 495.3325 | [M + H]+ | [C5H15O4NP]+ | 496.35 >184.051 | -40 | -28 | -40 | 20.56±0.01 |
| LPC 18:0 | LPC | C26H54N1O7P1 | 523.3638 | [M + H]+ | [C5H15O4NP]+ | 524.35 >184.051 | -40 | -28 | -40 | 21.62±0.01 |
| LPC 18:1 | LPC | C26H52N1O7P1 | 521.3481 | [M + H]+ | [C5H15O4NP]+ | 522.35 >184.051 | -40 | -28 | -40 | 20.90±0.00 |
| LPC 18:2 | LPC | C26H50N1O7P1 | 519.3325 | [M + H]+ | [C5H15O4NP]+ | 520.35 >184.051 | -40 | -28 | -40 | 20.05±0.01 |
| LPC 20:4 | LPC | C28H50N1O7P1 | 543.3325 | [M + H]+ | [C5H15O4NP]+ | 544.35 >184.051 | -40 | -28 | -40 | 20.09±0.01 |
| LPC 20:5 | LPC | C28H48N1O7P1 | 541.3168 | [M + H]+ | [C5H15O4NP]+ | 542.3 >184.051 | -40 | -28 | -40 | 19.48±0.01 |
| LPC 22:5 | LPC | C30H52N1O7P1 | 569.3481 | [M + H]+ | [C5H15O4NP]+ | 570.35 >184.051 | -40 | -28 | -40 | 20.69±0.01 |
| LPC 22:6 | LPC | C30H50N1O7P1 | 567.3325 | [M + H]+ | [C5H15O4NP]+ | 568.35 >184.051 | -40 | -28 | -40 | 20.29±0.01 |
| LPE 16:0 | LPE | C21H44N1O7P1 | 453.2855 | [M + H]+ | [M + H - C2H8O4NP]+ | 454.3 >313.251 | -40 | -19 | -40 | 21.04±0.01 |
| LPE 18:0 | LPE | C23H48N1O7P1 | 481.3168 | [M + H]+ | [M + H - C2H8O4NP]+ | 482.3 >341.31 | -40 | -19 | -40 | 22.00±0.01 |
| LPE 18:1 | LPE | C23H46N1O7P1 | 479.3012 | [M + H]+ | [M + H - C2H8O4NP]+ | 480.3 >339.31 | -40 | -19 | -40 | 21.29±0.00 |
| LPE 18:2 | LPE | C23H44N1O7P1 | 477.2855 | [M + H]+ | [M + H - C2H8O4NP]+ | 478.3 >337.251 | -40 | -19 | -40 | 20.57±0.01 |
| LPE 20:4 | LPE | C25H44N1O7P1 | 501.2855 | [M + H]+ | [M + H - C2H8O4NP]+ | 502.3 >361.251 | -40 | -19 | -40 | 20.56±0.00 |
| LPE 20:5 | LPE | C25H42N1O7P1 | 499.2699 | [M + H]+ | [M + H - C2H8O4NP]+ | 500.3 >359.251 | -40 | -19 | -40 | 19.74±0.00 |
| LPE 22:5 | LPE | C27H46N1O7P1 | 527.3012 | [M + H]+ | [M + H - C2H8O4NP]+ | 528.3 >387.31 | -40 | -19 | -40 | 21.10±0.08 |
| LPE 22:6 | LPE | C27H44N1O7P1 | 525.2855 | [M + H]+ | [M + H - C2H8O4NP]+ | 526.3 >385.251 | -40 | -19 | -40 | 20.51±0.00 |
| PC 16:0_16:0 | PC | C40H80N1O8P1 | 733.5622 | [M + CH3COO]- | [Acyl FA - H]- | 792.6 >255.251 | 40 | 39 | 40 | 24.49±0.01 |
| PC 16:0_18:0 | PC | C42H84N1O8P1 | 761.5935 | [M + CH3COO]- | [Acyl FA - H]- | 820.6 >255.251, 820.6 >283.251 | 40 | 39 | 40 | 24.65±0.13 |
| PC 18:0_18:0 | PC | C44H88N1O8P1 | 789.6248 | [M + CH3COO]- | [Acyl FA - H]- | 848.65 >283.251 | 40 | 39 | 40 | 24.90±0.02 |
| PC 16:0_18:1 | PC | C42H82N1O8P1 | 759.5778 | [M + CH3COO]- | [Acyl FA - H]- | 818.6 >255.251, 818.6 >281.251 | 40 | 39 | 40 | 24.58±0.00 |
| PC 18:0_18:1 | PC | C44H86N1O8P1 | 787.6091 | [M + CH3COO]- | [Acyl FA - H]- | 846.6 >283.251, 846.6 >281.251 | 40 | 39 | 40 | 24.90±0.01 |
| PC 18:1_18:1 | PC | C44H84N1O8P1 | 785.5935 | [M + CH3COO]- | [Acyl FA - H]- | 844.6 >281.251 | 40 | 39 | 40 | 24.63±0.01 |
| PC 16:0_18:2 | PC | C42H80N1O8P1 | 757.5622 | [M + CH3COO]- | [Acyl FA - H]- | 816.6 >255.251, 816.6 >279.251 | 40 | 39 | 40 | 24.33±0.02 |
| PC 18:0_18:2 | PC | C44H84N1O8P1 | 785.5935 | [M + CH3COO]- | [Acyl FA - H]- | 844.6 >283.251, 844.6 >279.251 | 40 | 39 | 40 | 24.69±0.01 |
| PC 18:1_18:2 | PC | C44H82N1O8P1 | 783.5778 | [M + CH3COO]- | [Acyl FA - H]- | 842.6 >281.251, 842.6 >279.251 | 40 | 39 | 40 | 24.44±0.01 |
| PC 18:2_18:2 | PC | C44H80N1O8P1 | 781.5622 | [M + CH3COO]- | [Acyl FA - H]- | 840.6 >279.251 | 40 | 39 | 40 | 24.19±0.01 |
| PC 16:0_20:4 | PC | C44H80N1O8P1 | 781.5622 | [M + CH3COO]- | [Acyl FA - H]- | 840.6 >255.251, 840.6 >303.251 | 40 | 39 | 40 | 24.35±0.01 |
| PC 18:0_20:4 | PC | C46H84N1O8P1 | 809.5935 | [M + CH3COO]- | [Acyl FA - H]- | 868.6 >283.251, 868.6 >303.251 | 40 | 39 | 40 | 24.70±0.01 |
| PC 18:1_20:4 | PC | C46H82N1O8P1 | 807.5778 | [M + CH3COO]- | [Acyl FA - H]- | 866.6 >281.251, 866.6 >303.251 | 40 | 39 | 40 | 24.44±0.01 |
| PC 18:2_20:4 | PC | C46H80N1O8P1 | 805.5622 | [M + CH3COO]- | [Acyl FA - H]- | 864.6 >279.251, 864.6 >303.251 | 40 | 39 | 40 | 24.18±0.01 |
| PC 20:4_20:4 | PC | C48H80N1O8P1 | 829.5622 | [M + CH3COO]- | [Acyl FA - H]- | 888.6 >303.251 | 40 | 39 | 40 | 24.12±0.10 |
| PC 16:0_20:5 | PC | C44H78N1O8P1 | 779.5465 | [M + CH3COO]- | [Acyl FA - H]- | 838.55 >255.251, 838.55 >301.21 | 40 | 39 | 40 | 24.15±0.01 |
| PC 18:0_20:5 | PC | C46H82N1O8P1 | 807.5778 | [M + CH3COO]- | [Acyl FA - H]- | 866.6 >283.251, 866.6 >301.21 | 40 | 39 | 40 | 24.51±0.01 |
| PC 18:1_20:5 | PC | C46H80N1O8P1 | 805.5622 | [M + CH3COO]- | [Acyl FA - H]- | 864.6 >281.251, 864.6 >301.21 | 40 | 39 | 40 | 24.16±0.05 |
| PC 18:2_20:5 | PC | C46H78N1O8P1 | 803.5465 | [M + CH3COO]- | [Acyl FA - H]- | 862.55 >279.251, 862.55 >301.21 | 40 | 39 | 40 | 23.90±0.02 |
| PC 20:4_20:5 | PC | C48H78N1O8P1 | 827.5465 | [M + CH3COO]- | [Acyl FA - H]- | 886.55 >303.251, 886.55 >301.21 | 40 | 39 | 40 | 23.91±0.03 |
| PC 20:5_20:5 | PC | C48H76N1O8P1 | 825.5309 | [M + CH3COO]- | [Acyl FA - H]- | 884.55 >301.21 | 40 | 39 | 40 | 23.74±0.03 |
| PC 16:0_22:5 | PC | C46H82N1O8P1 | 807.5778 | [M + CH3COO]- | [Acyl FA - H]- | 866.6 >255.251, 866.6 >329.251 | 40 | 39 | 40 | 24.43±0.01 |
| PC 18:0_22:5 | PC | C48H86N1O8P1 | 835.6091 | [M + CH3COO]- | [Acyl FA - H]- | 894.6 >283.251, 894.6 >329.251 | 40 | 39 | 40 | 24.80±0.02 |
| PC 18:1_22:5 | PC | C48H84N1O8P1 | 833.5935 | [M + CH3COO]- | [Acyl FA - H]- | 892.6 >281.251, 892.6 >329.251 | 40 | 39 | 40 | 24.50±0.03 |
| PC 18:2_22:5 | PC | C48H82N1O8P1 | 831.5778 | [M + CH3COO]- | [Acyl FA - H]- | 890.6 >279.251, 890.6 >329.251 | 40 | 39 | 40 | 24.22±0.03 |
| PC 20:4_22:5 | PC | C50H82N1O8P1 | 855.5778 | [M + CH3COO]- | [Acyl FA - H]- | 914.6 >303.251, 914.6 >329.251 | 40 | 39 | 40 | 24.22±0.03 |
| PC 20:5_22:5 | PC | C50H80N1O8P1 | 853.5622 | [M + CH3COO]- | [Acyl FA - H]- | 912.6 >301.21, 912.6 >329.251 | 40 | 39 | 40 | 24.07±0.00 |
| PC 22:5_22:5 | PC | C52H84N1O8P1 | 881.5935 | [M + CH3COO]- | [Acyl FA - H]- | 940.6 >329.251 | 40 | 39 | 40 | 24.95±0.12 |
| PC 16:0_22:6 | PC | C46H80N1O8P1 | 805.5622 | [M + CH3COO]- | [Acyl FA - H]- | 864.6 >255.251, 864.6 >327.251 | 40 | 39 | 40 | 24.29±0.01 |
| PC 18:0_22:6 | PC | C48H84N1O8P1 | 833.5935 | [M + CH3COO]- | [Acyl FA - H]- | 892.6 >283.251, 892.6 >327.251 | 40 | 39 | 40 | 24.66±0.01 |
| PC 18:1_22:6 | PC | C48H82N1O8P1 | 831.5778 | [M + CH3COO]- | [Acyl FA - H]- | 890.6 >281.251, 890.6 >327.251 | 40 | 39 | 40 | 24.41±0.01 |
| PC 18:2_22:6 | PC | C48H80N1O8P1 | 829.5622 | [M + CH3COO]- | [Acyl FA - H]- | 888.6 >279.251, 888.6 >327.251 | 40 | 39 | 40 | 24.15±0.02 |
| PC 20:4_22:6 | PC | C50H80N1O8P1 | 853.5622 | [M + CH3COO]- | [Acyl FA - H]- | 912.6 >303.251, 912.6 >327.251 | 40 | 39 | 40 | 24.32±0.29 |
| PC 20:5_22:6 | PC | C50H78N1O8P1 | 851.5465 | [M + CH3COO]- | [Acyl FA - H]- | 910.55 >301.21, 910.55 >327.251 | 40 | 39 | 40 | 23.67±0.04 |
| PC 22:5_22:6 | PC | C52H82N1O8P1 | 879.5778 | [M + CH3COO]- | [Acyl FA - H]- | 938.6 >329.251, 938.6 >327.251 | 40 | 39 | 40 | 24.86±0.04 |
| PC 22:6_22:6 | PC | C52H80N1O8P1 | 877.5622 | [M + CH3COO]- | [Acyl FA - H]- | 936.6 >327.251 | 40 | 39 | 40 | 24.79±0.18 |
| TG 16:0_16:0_16:0 | TG | C51H98O6 | 806.7363 | [M + NH4]+ | [M + H - Acyl FA]+ | 824.75 >551.5 | -40 | -26 | -40 | 26.97±0.01 |
| TG 16:0_16:0_18:0 | TG | C53H102O6 | 834.7676 | [M + NH4]+ | [M + H - Acyl FA]+ | 852.8 >579.55 , 852.8 >551.5 | -40 | -26 | -40 | 27.09±0.04 |
| TG 16:0_18:0_18:0 | TG | C55H106O6 | 862.7989 | [M + NH4]+ | [M + H - Acyl FA]+ | 880.85 >607.55 , 880.85 >579.55 | -40 | -26 | -40 | 27.17±0.06 |
| TG 18:0_18:0_18:0 | TG | C57H110O6 | 890.8302 | [M + NH4]+ | [M + H - Acyl FA]+ | 908.85 >607.55 | -40 | -26 | -40 | 27.30±0.06 |
| TG 16:0_16:0_18:1 | TG | C53H100O6 | 832.7520 | [M + NH4]+ | [M + H - Acyl FA]+ | 850.8 >577.5 , 850.8 >551.5 | -40 | -26 | -40 | 26.98±0.01 |
| TG 16:0_18:0_18:1 | TG | C55H104O6 | 860.7833 | [M + NH4]+ | [M + H - Acyl FA]+ | 878.8 >605.55 , 878.8 >577.5 | -40 | -26 | -40 | 27.11±0.01 |
| TG 18:0_18:0_18:1 | TG | C57H108O6 | 888.8146 | [M + NH4]+ | [M + H - Acyl FA]+ | 906.85 >605.55 , 906.85 >607.55 | -40 | -26 | -40 | 27.24±0.04 |
| TG 16:0_18:1_18:1 | TG | C55H102O6 | 858.7676 | [M + NH4]+ | [M + H - Acyl FA]+ | 876.8 >603.55 , 876.8 >577.5 | -40 | -26 | -40 | 27.00±0.01 |
| TG 18:0_18:1_18:1 | TG | C57H106O6 | 886.7989 | [M + NH4]+ | [M + H - Acyl FA]+ | 904.85 >603.55 , 904.85 >605.55 | -40 | -26 | -40 | 27.14±0.02 |
| TG 18:1_18:1_18:1 | TG | C57H104O6 | 884.7833 | [M + NH4]+ | [M + H - Acyl FA]+ | 902.8 >603.55 | -40 | -26 | -40 | 27.02±0.01 |
| TG 16:0_16:0_18:2 | TG | C53H98O6 | 830.7363 | [M + NH4]+ | [M + H - Acyl FA]+ | 848.75 >575.5 , 848.75 >551.5 | -40 | -26 | -40 | 26.88±0.01 |
| TG 16:0_18:0_18:2 | TG | C55H102O6 | 858.7676 | [M + NH4]+ | [M + H - Acyl FA]+ | 876.8 >603.55 , 876.8 >575.5 | -40 | -26 | -40 | 27.00±0.00 |
| TG 18:0_18:0_18:2 | TG | C57H106O6 | 886.7989 | [M + NH4]+ | [M + H - Acyl FA]+ | 904.85 >603.55 , 904.85 >607.55 | -40 | -26 | -40 | 27.14±0.01 |
| TG 16:0_18:1_18:2 | TG | C55H100O6 | 856.7520 | [M + NH4]+ | [M + H - Acyl FA]+ | 874.8 >601.5 , 874.8 >575.5 | -40 | -26 | -40 | 26.90±0.01 |
| TG 18:0_18:1_18:2 | TG | C57H104O6 | 884.7833 | [M + NH4]+ | [M + H - Acyl FA]+ | 902.8 >601.5 , 902.8 >603.55 | -40 | -26 | -40 | 27.04±0.01 |
| TG 18:1_18:1_18:2 | TG | C57H102O6 | 882.7676 | [M + NH4]+ | [M + H - Acyl FA]+ | 900.8 >601.5 , 900.8 >603.55 | -40 | -26 | -40 | 26.92±0.01 |
| TG 16:0_18:2_18:2 | TG | C55H98O6 | 854.7363 | [M + NH4]+ | [M + H - Acyl FA]+ | 872.75 >599.5 , 872.75 >575.5 | -40 | -26 | -40 | 26.81±0.01 |
| TG 18:0_18:2_18:2 | TG | C57H102O6 | 882.7676 | [M + NH4]+ | [M + H - Acyl FA]+ | 900.8 >599.5 , 900.8 >603.55 | -40 | -26 | -40 | 26.95±0.02 |
| TG 18:1_18:2_18:2 | TG | C57H100O6 | 880.7520 | [M + NH4]+ | [M + H - Acyl FA]+ | 898.8 >599.5 , 898.8 >601.5 | -40 | -26 | -40 | 26.83±0.01 |
| TG 18:2_18:2_18:2 | TG | C57H98O6 | 878.7363 | [M + NH4]+ | [M + H - Acyl FA]+ | 896.75 >599.5 | -40 | -26 | -40 | 26.73±0.01 |
| TG 16:0_16:0_20:4 | TG | C55H98O6 | 854.7363 | [M + NH4]+ | [M + H - Acyl FA]+ | 872.75 >599.5 , 872.75 >551.5 | -40 | -26 | -40 | 26.81±0.01 |
| TG 16:0_18:0_20:4 | TG | C57H102O6 | 882.7676 | [M + NH4]+ | [M + H - Acyl FA]+ | 900.8 >627.55 , 900.8 >599.5 | -40 | -26 | -40 | 26.95±0.02 |
| TG 18:0_18:0_20:4 | TG | C59H106O6 | 910.7989 | [M + NH4]+ | [M + H - Acyl FA]+ | 928.85 >627.55 , 928.85 >607.55 | -40 | -26 | -40 | 27.08±0.02 |
| TG 16:0_18:1_20:4 | TG | C57H100O6 | 880.7520 | [M + NH4]+ | [M + H - Acyl FA]+ | 898.8 >625.5 , 898.8 >599.5 | -40 | -26 | -40 | 26.87±0.02 |
| TG 18:0_18:1_20:4 | TG | C59H104O6 | 908.7833 | [M + NH4]+ | [M + H - Acyl FA]+ | 926.8 >625.5 , 926.8 >627.55 | -40 | -26 | -40 | 27.02±0.02 |
| TG 18:1_18:1_20:4 | TG | C59H102O6 | 906.7676 | [M + NH4]+ | [M + H - Acyl FA]+ | 924.8 >625.5 , 924.8 >603.55 | -40 | -26 | -40 | 26.91±0.01 |
| TG 16:0_18:2_20:4 | TG | C57H98O6 | 878.7363 | [M + NH4]+ | [M + H - Acyl FA]+ | 896.75 >623.5 , 896.75 >599.5 | -40 | -26 | -40 | 26.85±0.15 |
| TG 18:0_18:2_20:4 | TG | C59H102O6 | 906.7676 | [M + NH4]+ | [M + H - Acyl FA]+ | 924.8 >623.5 , 924.8 >627.55 | -40 | -26 | -40 | 26.94±0.01 |
| TG 18:1_18:2_20:4 | TG | C59H100O6 | 904.7520 | [M + NH4]+ | [M + H - Acyl FA]+ | 922.8 >623.5 , 922.8 >625.5 | -40 | -26 | -40 | 26.82±0.02 |
| TG 18:2_18:2_20:4 | TG | C59H98O6 | 902.7363 | [M + NH4]+ | [M + H - Acyl FA]+ | 920.75 >623.5 , 920.75 >599.5 | -40 | -26 | -40 | 26.72±0.01 |
| TG 16:0_20:4_20:4 | TG | C59H98O6 | 902.7363 | [M + NH4]+ | [M + H - Acyl FA]+ | 920.75 >647.5 , 920.75 >599.5 | -40 | -26 | -40 | 27.03±0.27 |
| TG 18:0_20:4_20:4 | TG | C61H102O6 | 930.7676 | [M + NH4]+ | [M + H - Acyl FA]+ | 948.8 >647.5 , 948.8 >627.55 | -40 | -26 | -40 | 26.91±0.02 |
| TG 18:1_20:4_20:4 | TG | C61H100O6 | 928.7520 | [M + NH4]+ | [M + H - Acyl FA]+ | 946.8 >647.5 , 946.8 >625.5 | -40 | -26 | -40 | 26.99±0.26 |
| TG 18:2_20:4_20:4 | TG | C61H98O6 | 926.7363 | [M + NH4]+ | [M + H - Acyl FA]+ | 944.75 >647.5 , 944.75 >623.5 | -40 | -26 | -40 | 26.76±0.15 |
| TG 20:4_20:4_20:4 | TG | C63H98O6 | 950.7363 | [M + NH4]+ | [M + H - Acyl FA]+ | 968.75 >647.5 | -40 | -26 | -40 | 26.68±0.00 |
| TG 16:0_16:0_22:6 | TG | C57H98O6 | 878.7363 | [M + NH4]+ | [M + H - Acyl FA]+ | 896.75 >623.5 , 896.75 >551.5 | -40 | -26 | -40 | 26.78±0.06 |
| TG 16:0_18:0_22:6 | TG | C59H102O6 | 906.7676 | [M + NH4]+ | [M + H - Acyl FA]+ | 924.8 >651.55 , 924.8 >623.5 | -40 | -26 | -40 | 26.91±0.03 |
| TG 18:0_18:0_22:6 | TG | C61H106O6 | 934.7989 | [M + NH4]+ | [M + H - Acyl FA]+ | 952.85 >651.55 , 952.85 >607.55 | -40 | -26 | -40 | 27.04±0.02 |
| TG 16:0_18:1_22:6 | TG | C59H100O6 | 904.7520 | [M + NH4]+ | [M + H - Acyl FA]+ | 922.8 >649.5 , 922.8 >623.5 | -40 | -26 | -40 | 27.04±0.27 |
| TG 18:0_18:1_22:6 | TG | C61H104O6 | 932.7833 | [M + NH4]+ | [M + H - Acyl FA]+ | 950.8 >649.5 , 950.8 >651.55 | -40 | -26 | -40 | 26.95±0.01 |
| TG 18:1_18:1_22:6 | TG | C61H102O6 | 930.7676 | [M + NH4]+ | [M + H - Acyl FA]+ | 948.8 >649.5 , 948.8 >603.55 | -40 | -26 | -40 | 27.00±0.23 |
| TG 16:0_18:2_22:6 | TG | C61H102O6 | 930.7676 | [M + NH4]+ | [M + H - Acyl FA]+ | 920.75 >647.5 , 920.75 >623.5 | -40 | -26 | -40 | 27.02±0.28 |
| TG 18:0_18:2_22:6 | TG | C61H102O6 | 930.7676 | [M + NH4]+ | [M + H - Acyl FA]+ | 948.8 >647.5 , 948.8 >651.55 | -40 | -26 | -40 | 26.91±0.01 |
| TG 18:1_18:2_22:6 | TG | C61H100O6 | 928.7520 | [M + NH4]+ | [M + H - Acyl FA]+ | 946.8 >647.5 , 946.8 >649.5 | -40 | -26 | -40 | 27.01±0.27 |
| TG 18:2_18:2_22:6 | TG | C61H98O6 | 926.7363 | [M + NH4]+ | [M + H - Acyl FA]+ | 944.75 >647.5 , 944.75 >599.5 | -40 | -26 | -40 | 26.70±0.01 |
| TG 16:0_20:4_22:6 | TG | C61H98O6 | 926.7363 | [M + NH4]+ | [M + H - Acyl FA]+ | 944.75 >671.5 , 944.75 >623.5 | -40 | -26 | -40 | 26.76±0.02 |
| TG 18:0_20:4_22:6 | TG | C63H102O6 | 954.7676 | [M + NH4]+ | [M + H - Acyl FA]+ | 972.8 >671.5 , 972.8 >651.55 | -40 | -26 | -40 | 26.90±0.02 |
| TG 18:1_20:4_22:6 | TG | C63H100O6 | 952.7520 | [M + NH4]+ | [M + H - Acyl FA]+ | 970.8 >671.5 , 970.8 >649.5 | -40 | -26 | -40 | 27.05±0.21 |
| TG 18:2_20:4_22:6 | TG | C63H98O6 | 950.7363 | [M + NH4]+ | [M + H - Acyl FA]+ | 968.75 >671.5 , 968.75 >647.5 | -40 | -26 | -40 | 26.69±0.02 |
| TG 20:4_20:4_22:6 | TG | C65H98O6 | 974.7363 | [M + NH4]+ | [M + H - Acyl FA]+ | 992.75 >671.5 , 992.75 >647.5 | -40 | -26 | -40 | 26.68±0.02 |
| TG 16:0_22:6_22:6 | TG | C63H98O6 | 950.7363 | [M + NH4]+ | [M + H - Acyl FA]+ | 968.75 >695.5 , 968.75 >623.5 | -40 | -26 | -40 | 26.75±0.01 |
| TG 18:0_22:6_22:6 | TG | C65H102O6 | 978.7676 | [M + NH4]+ | [M + H - Acyl FA]+ | 996.8 >695.5 , 996.8 >651.55 | -40 | -26 | -40 | ND |
| TG 18:1_22:6_22:6 | TG | C65H100O6 | 976.7520 | [M + NH4]+ | [M + H - Acyl FA]+ | 994.8 >695.5 , 994.8 >649.5 | -40 | -26 | -40 | ND |
| TG 18:2_22:6_22:6 | TG | C65H98O6 | 974.7363 | [M + NH4]+ | [M + H - Acyl FA]+ | 992.75 >695.5 , 992.75 >647.5 | -40 | -26 | -40 | ND |
| TG 20:4_22:6_22:6 | TG | C67H98O6 | 998.7363 | [M + NH4]+ | [M + H - Acyl FA]+ | 1016.75 >695.5 , 1016.75 >671.5 | -40 | -26 | -40 | ND |
| TG 22:6_22:6_22:6 | TG | C69H98O6 | 1022.7363 | [M + NH4]+ | [M + H - Acyl FA]+ | 1040.75 >695.5 | -40 | -26 | -40 | ND |

**Supplemental Table S10.** Quantitative analysis of bioactive lipids in the plasma of healthy volunteers.

| Compound name | RT (min) | ISTD | Quantification (nM in plasma) | | | | | | | | | | | | | | | | | | | | | | | |
| --- | --- | --- | --- | --- | --- | --- | --- | --- | --- | --- | --- | --- | --- | --- | --- | --- | --- | --- | --- | --- | --- | --- | --- | --- | --- | --- |
|  |  |  | P1 | P2 | P3 | P4 | P5 | P6 | P7 | P8 | P9 | P10 | P11 | P12 | P13 | P14 | P15 | P16 | P17 | P18 | P19 | P20 | P21 | P22 | P23 | P24 |
| EPA | 20.71±0.11 | EPA-d5 (IS) | 1806.0 | 1290.4 | 461.1 | 398.7 | 347.8 | 632.9 | 1520.7 | 2060.0 | 1321.2 | 869.6 | 1525.2 | 1623.3 | 2239.4 | 443.2 | 869.3 | 885.1 | 1703.8 | 2080.6 | 1308.5 | 2433.0 | 2321.3 | 1005.6 | 726.9 | 1930.1 |
| AA | 21.52±0.09 | AA-d8 (IS) | 8237.1 | 7432.3 | 2944.6 | 2236.7 | 1878.5 | 3293.3 | 4785.7 | 7008.1 | 4196.7 | 3367.9 | 6310.5 | 5512.1 | 8213.1 | 2130.3 | 3955.9 | 4908.9 | 3272.3 | 5089.0 | 2735.0 | 6561.0 | 7997.1 | 4701.5 | 5727.4 | 6729.7 |
| DHA | 21.30±0.08 | DHA-d5 (IS) | 6564.8 | 5571.4 | 1789.9 | 1569.1 | 956.3 | 3494.6 | 7707.7 | 11597.8 | 5588.3 | 3641.0 | 8422.0 | 7881.3 | 10681.6 | 1847.5 | 4334.8 | 4335.2 | 3704.9 | 6197.3 | 3077.4 | 7574.8 | 8085.0 | 4206.6 | 5807.2 | 8881.2 |
| 5-HETE | 18.14±0.05 | 15-HETE-d8 (IS) | 2.3 | 1.4 | ND | ND | ND | ND | ND | 1.1 | ND | ND | ND | 1.6 | 1.1 | ND | ND | ND | ND | ND | ND | 2.8 | 2.9 | ND | ND | 0.9 |
| 12-HETE | 16.90±0.00 | 15-HETE-d8 (IS) | 2.7 | 7.8 | 7.7 | 3.1 | 3.7 | 4.5 | 3.1 | 5.6 | 7.8 | 8.1 | 8.0 | 8.1 | 9.8 | 1.7 | 7.1 | 2.4 | 7.9 | 6.3 | 4.6 | 10.2 | 9.2 | 2.4 | 9.8 | 4.8 |
| 15-HETE | 16.17±0.05 | 15-HETE-d8 (IS) | 2.5 | 4.6 | 2.4 | 2.3 | 2.6 | ND | 2.3 | 3.0 | 2.9 | ND | 3.8 | 2.3 | 3.0 | ND | 1.7 | 2.4 | 1.5 | 1.7 | ND | 3.6 | 4.6 | ND | 2.6 | ND |
| 17-HDoHE | 16.63±0.08 | 15-HETE-d8 (IS) | ND | 24.1 | 30.9 | ND | ND | ND | 17.2 | 38.3 | 30.7 | 24.8 | 18.7 | 26.4 | 41.7 | ND | ND | ND | 26.0 | 28.7 | ND | 28.7 | 39.9 | ND | 21.9 | ND |
| E2-3-G-17-S | 1.53±0.03 | 18-OHB-d4 (IS) | 6.2 | 2.4 | 4.9 | 6.5 | 5.1 | 3.3 | 4.5 | 4.0 | 6.0 | 3.3 | 1.7 | 5.0 | 2.1 | 3.6 | 4.6 | 9.7 | 3.4 | 4.0 | 3.9 | 1.7 | 2.7 | 1.8 | 3.7 | 1.5 |
| An-G (and Et-G) | 5.85±0.05 | E-d8 (IS) | 64.3 | 79.8 | 55.0 | 46.7 | 45.4 | 56.7 | 41.6 | 39.0 | 38.1 | 42.0 | 45.5 | 66.6 | 91.6 | 70.9 | 67.3 | ND | 65.3 | 59.5 | 45.6 | 93.4 | 63.2 | 36.5 | 27.3 | 15.3 |
| T | 11.01±0.05 | T-d3 (IS) | 15.3 | 14.2 | 19.9 | 21.9 | 19.0 | 28.1 | 15.1 | 10.2 | 8.9 | 8.4 | 16.5 | 20.0 | 10.5 | 14.3 | 17.8 | 12.0 | 18.3 | 16.9 | 13.1 | 19.9 | 21.2 | 0.8 | 1.3 | 0.6 |
| 7α-OHT | 4.67±0.06 | T-d3 (IS) | ND | 4.0 | ND | ND | ND | ND | ND | ND | ND | ND | ND | ND | ND | ND | ND | ND | ND | ND | ND | ND | ND | ND | ND | ND |
| T-G | 2.70±0.15 | T-d3 (IS) | 32.3 | 96.2 | 150.6 | 142.1 | 143.2 | 144.4 | 324.0 | 382.0 | 473.2 | 450.7 | 118.1 | 46.7 | 40.0 | 37.1 | 84.2 | 13.5 | 9.3 | 8.9 | 19.4 | 29.7 | 84.7 | 190.7 | 5.2 | 25.9 |
| Preg-S | 9.98±0.03 | Chol-S-d7 (IS) | ND | ND | ND | ND | ND | 43.4 | 117.9 | 55.6 | 161.2 | 84.7 | 146.4 | 45.0 | ND | 52.4 | 46.9 | 42.2 | 110.0 | 162.1 | 104.7 | 74.2 | 111.9 | 53.4 | 38.3 | 92.5 |
| Prog | 15.22±0.09 | T-d3 (IS) | 0.3 | 0.3 | 0.4 | 0.3 | ND | 0.3 | 0.3 | ND | 0.3 | 0.3 | 0.3 | 0.3 | ND | 0.3 | 0.3 | ND | 0.3 | 0.2 | 0.2 | 0.3 | 0.3 | 29.8 | 0.2 | 15.3 |
| 20α-OHProg | 14.17±0.08 | T-d3 (IS) | 0.3 | 0.3 | 0.2 | ND | 0.2 | 0.3 | 0.2 | ND | 0.1 | 0.2 | 0.2 | 0.2 | 0.2 | 0.2 | ND | ND | 0.3 | 0.2 | 0.2 | 0.3 | 0.2 | 1.8 | 0.2 | 2.2 |
| 17α-OHProg | 11.02±0.04 | T-d3 (IS) | 29.4 | 34.7 | 37.2 | 41.1 | 36.3 | 46.6 | 26.7 | 21.4 | 22.8 | 20.8 | 28.5 | 35.7 | 27.9 | 29.1 | 35.4 | 23.2 | 32.1 | 32.4 | 27.7 | 35.8 | 37.8 | ND | ND | ND |
| B (and 21-DOF) | 8.14±0.07 | B-d8 (IS) | 6.9 | 11.6 | 7.1 | 8.4 | 11.0 | 15.7 | 7.8 | 10.9 | 9.5 | 6.2 | 5.5 | 8.9 | 13.6 | 5.9 | 11.3 | 93.2 | 12.6 | 10.5 | 7.9 | 20.9 | 7.3 | 7.4 | 6.3 | 20.3 |
| DOC | 11.02±0.04 | DOC-d8 (IS) | 3.0 | 3.1 | 2.7 | 3.8 | 0.9 | 4.5 | 3.0 | 2.5 | 1.9 | 2.6 | 2.5 | 3.5 | 2.8 | 2.4 | 3.3 | 2.5 | 3.7 | 3.2 | 2.7 | 3.6 | 3.6 | 0.8 | 1.3 | 1.4 |
| F | 5.83±0.08 | F-d4 (IS) | 1487.3 | 1063.3 | 1784.6 | 1177.6 | 1046.7 | 2992.5 | 608.6 | 1864.6 | 986.7 | 1361.3 | 1280.7 | 1606.4 | 328.4 | 1114.0 | 1913.9 | 196.0 | 2329.0 | 1829.4 | 530.2 | 2390.9 | 2001.6 | 535.6 | 590.0 | 2528.5 |
| E | 5.80±0.09 | E-d8 (IS) | 123.6 | 69.2 | 137.7 | 166.2 | 84.3 | 153.8 | 102.8 | 157.7 | 115.4 | 154.0 | 96.2 | 156.1 | ND | 48.8 | 170.9 | ND | 153.7 | 153.0 | 111.8 | 44.6 | 95.0 | 23.3 | 105.8 | 179.0 |
| A | 4.48±0.32 | B-d8 (IS) | 12.8 | 40.1 | 28.7 | 17.3 | 39.7 | 36.2 | 32.8 | 24.8 | 30.9 | 35.0 | 42.6 | 10.1 | ND | 12.4 | ND | ND | 11.7 | 12.3 | ND | 17.8 | 22.4 | ND | 187.1 | ND |
| 5β-DiHE | 7.65±0.07 | B-d8 (IS) | 5.4 | 5.3 | 16.3 | 7.6 | 7.5 | 17.4 | 3.7 | 7.3 | 5.6 | 6.7 | 5.9 | 10.5 | ND | 3.9 | 13.2 | ND | 7.0 | 8.4 | 8.9 | 6.5 | 4.0 | 4.4 | 5.4 | 8.6 |
| DHEA-S | 6.84±0.02 | T-d3 (IS) | 3.2 | 2.6 | 0.6 | 0.8 | 7.5 | 5.8 | 58.7 | 11.0 | 75.7 | 46.7 | 128.2 | 11.5 | 8.1 | 8.7 | 11.5 | 2.0 | 25.6 | 35.2 | 22.7 | 7.8 | 55.7 | 38.7 | 15.9 | 45.1 |
| 7-keto-Chol | 23.05±0.09 | Chol-S-d7 (IS) | 1302.3 | 1466.9 | 2925.9 | 2830.1 | 2440.2 | 1232.9 | 2441.0 | 3264.8 | 2242.5 | 2920.2 | 1874.9 | 2202.0 | 6300.8 | 3480.3 | 1788.1 | 2575.9 | 2872.2 | 2050.8 | 2797.9 | 2408.3 | 1126.5 | 1091.6 | 1615.6 | 703.6 |
| Chol-S | 22.34±0.11 | Chol-S-d7 (IS) | 10442.4 | 12430.3 | 9613.2 | 8752.5 | 7755.4 | 7890.7 | 10658.3 | 12390.2 | 11018.5 | 9788.6 | 9374.0 | 8247.2 | 18238.6 | 8109.3 | 8504.3 | 6714.0 | 7123.6 | 7352.8 | 8750.2 | 10504.2 | 8505.2 | 6842.6 | 7675.3 | 7266.7 |
| 7α-OHChol | 23.05±0.00 | 24-OHChol-d7 (IS) | 156.3 | 143.8 | 158.5 | 155.9 | 159.4 | 170.6 | 124.2 | 122.5 | 122.2 | 181.4 | 132.7 | 168.3 | 170.6 | 187.0 | 146.3 | 154.8 | 197.5 | 152.9 | 171.6 | 156.7 | 134.0 | 129.3 | 153.5 | 103.3 |
| TCA | 7.66±0.07 | TCA-d5 (IS) | 12.2 | 34.8 | 55.1 | 86.4 | 79.1 | 89.3 | 24.9 | 7.4 | 63.1 | 24.5 | 12.3 | 10.2 | 11.5 | 36.1 | 8.1 | 42.7 | 161.6 | 42.0 | 167.0 | 293.3 | 121.3 | 57.3 | 65.2 | 47.1 |
| TαMCA | 4.26±0.09 | CA-d4 (IS) | ND | ND | 0.8 | 0.7 | 6.2 | 3.3 | 4.7 | 1.2 | 7.3 | 3.1 | 0.8 | 1.3 | ND | 0.8 | 2.9 | 1.2 | 2.4 | 1.1 | 6.6 | 0.7 | ND | ND | 21.8 | 12.6 |
| TDCA | 11.49±0.05 | DCA-d4 (IS) | 4.7 | 7.2 | 3.1 | 3.9 | 17.7 | 57.2 | 46.4 | 5.6 | 147.7 | 80.4 | 10.1 | 14.1 | 11.8 | 37.7 | 10.9 | 29.8 | 104.6 | 29.1 | 151.1 | 72.5 | 24.7 | 415.2 | 118.5 | 233.2 |
| TUDCA | 7.19±0.12 | DCA-d4 (IS) | 7.5 | 7.1 | ND | ND | 14.8 | 9.1 | 89.5 | 13.1 | 257.6 | 109.8 | 34.3 | 12.0 | 14.6 | 44.5 | 10.8 | 44.5 | 500.6 | 155.8 | 825.0 | 500.7 | 219.7 | 126.8 | 38.9 | 85.5 |
| GCA | 7.16±0.04 | GCA-d4 (IS) | 89.4 | 252.8 | 156.7 | 150.5 | 197.1 | 193.1 | 184.6 | 48.7 | 348.4 | 160.4 | 83.1 | 73.5 | 66.9 | 269.0 | 60.8 | 2143.7 | 351.5 | 93.0 | 454.8 | 540.1 | 296.6 | 85.4 | 440.2 | 232.3 |
| GUDCA | 6.47±0.08 | DCA-d4 (IS) | 253.2 | 412.6 | 212.8 | 238.5 | 299.0 | 263.1 | 1255.8 | 654.0 | 2276.2 | 1636.2 | 549.9 | 770.9 | 868.0 | 1779.2 | 680.5 | 1179.7 | 2624.5 | 603.1 | 6171.7 | 4775.1 | 767.9 | 331.0 | 841.7 | 684.3 |
| GCDCA | 10.19±0.03 | DCA-d4 (IS) | 1205.7 | 2047.1 | 796.9 | 967.4 | 1409.3 | 1074.1 | 1971.7 | 1035.8 | 4597.4 | 3127.6 | 964.8 | 1653.2 | 2042.3 | 3920.2 | 1096.2 | 2589.6 | 2519.0 | 443.7 | 6198.8 | 5610.9 | 1239.6 | 1352.8 | 2245.0 | 3819.6 |
| GDCA | 11.06±0.04 | DCA-d4 (IS) | 511.2 | 808.5 | 1292.7 | 1659.3 | 1338.9 | 1975.7 | 420.4 | 231.4 | 1245.8 | 848.5 | 260.8 | 709.7 | 798.5 | 2131.6 | 569.0 | 508.8 | 861.3 | 176.0 | 1694.5 | 1222.1 | 116.5 | 875.4 | 3549.7 | 1811.2 |
| GLCA | 13.79±0.11 | LCA-d4 (IS) | 14.4 | 9.5 | 25.1 | 48.0 | 48.1 | 22.1 | 7.6 | 16.0 | 24.7 | 14.3 | 7.9 | 20.5 | 55.2 | 42.0 | 15.3 | 9.9 | 15.1 | 13.9 | 35.3 | 10.0 | 8.0 | 12.0 | 55.5 | 11.3 |
| CA | 8.44±0.09 | CA-d4 (IS) | 79.1 | 97.5 | 56.0 | 48.0 | 21.6 | 67.3 | 80.6 | 19.5 | 70.5 | 33.0 | 21.7 | 48.1 | 58.4 | 58.6 | 46.2 | 78.2 | 48.0 | 35.4 | 61.9 | 54.1 | 153.5 | 69.5 | 79.1 | 67.3 |
| ω-MCA | 4.01±0.43 | CA-d4 (IS) | 15.0 | 16.6 | 20.9 | 14.0 | 27.1 | 22.1 | 37.9 | 9.8 | 27.4 | 15.8 | 14.4 | 27.2 | 32.3 | 32.8 | 34.9 | 17.3 | 54.4 | 32.6 | 79.8 | 51.0 | 79.6 | 21.3 | 29.9 | 44.8 |
| α-MCA | 4.69±0.14 | CA-d4 (IS) | 27.1 | 23.6 | 30.3 | 21.0 | 31.7 | 31.3 | 41.5 | 14.3 | 49.6 | 26.0 | 17.8 | 37.5 | 51.6 | 46.9 | 40.2 | 81.5 | 54.5 | 51.5 | 65.2 | 48.0 | 89.2 | 33.6 | 25.2 | 43.8 |
| β-MCA | 4.99±0.14 | CA-d4 (IS) | ND | 6.5 | 14.6 | 12.1 | 16.2 | 15.7 | 9.2 | 8.0 | 12.2 | 8.4 | 8.2 | 12.7 | 15.2 | 11.5 | 13.3 | 12.4 | 56.7 | 55.8 | 64.6 | 41.5 | 36.4 | 11.9 | 29.3 | ND |
| DCA | 13.27±0.09 | DCA-d4 (IS) | 634.3 | 253.7 | 657.2 | 508.8 | 766.8 | 716.0 | 328.8 | 445.5 | 627.0 | 296.6 | 277.8 | 722.1 | 751.8 | 604.2 | 902.8 | 530.8 | 616.1 | 295.1 | 210.9 | 240.0 | 131.4 | 785.0 | 530.1 | 570.4 |
| UDCA | 8.50±0.13 | DCA-d4 (IS) | 342.8 | 160.1 | 297.4 | 259.7 | 303.0 | 339.2 | 649.2 | 440.0 | 685.2 | 411.3 | 444.8 | 464.6 | 655.7 | 387.2 | 719.0 | 773.9 | 1384.3 | 1150.3 | 868.2 | 951.2 | 1012.6 | 516.9 | 299.2 | 254.2 |
| HDCA | 9.54±0.18 | DCA-d4 (IS) | 2642.8 | 1042.1 | 1920.9 | 1366.6 | 1137.5 | 1961.2 | 742.0 | 983.9 | 1373.5 | 734.5 | 661.5 | 1088.3 | 1764.2 | 1053.9 | 1820.1 | 1488.8 | 2273.9 | 1964.6 | 1341.2 | 1113.2 | 1416.9 | 1454.5 | 764.2 | 1081.4 |
| CDCA | 12.67±0.10 | DCA-d4 (IS) | 674.3 | 593.3 | 95.6 | 79.0 | 109.7 | 121.4 | 1060.7 | 305.6 | 869.6 | 328.0 | 452.9 | 845.0 | 1035.4 | 475.8 | 1261.2 | 1946.9 | 933.1 | 483.4 | 349.9 | 456.6 | 1691.8 | 833.5 | 151.1 | 1233.4 |
| 7-keto-LCA | 9.45±0.13 | LCA-d4 (IS) | 12.3 | 6.6 | 5.8 | 4.6 | 6.9 | 5.0 | 18.8 | 13.4 | 21.3 | 16.8 | 18.5 | 26.5 | 45.7 | 27.2 | 24.0 | 29.8 | 39.9 | 49.7 | 27.9 | 37.8 | 29.5 | 13.2 | 7.9 | 10.2 |
| 12-keto-LCA | 9.85±0.13 | LCA-d4 (IS) | 11.0 | 7.8 | 30.4 | 27.7 | 6.5 | 6.6 | 4.2 | 10.9 | 13.4 | 10.5 | 6.0 | 28.8 | 67.2 | 32.3 | 24.7 | 16.0 | 19.7 | 14.1 | 8.7 | 9.9 | 5.0 | 12.2 | 27.0 | 6.9 |
| LCA | 17.18±0.15 | LCA-d4 (IS) | 29.4 | 9.1 | 18.7 | 23.1 | 40.3 | 33.8 | 5.2 | 23.4 | 38.7 | 18.5 | 8.9 | 33.9 | 24.8 | 25.8 | 25.9 | 9.6 | 32.7 | 24.7 | 14.6 | 7.5 | 6.2 | 8.1 | 27.9 | 6.3 |
